# Correction: Transcription Factors Encoded on Core and Accessory Chromosomes of *Fusarium oxysporum* Induce Expression of Effector Genes

**DOI:** 10.1371/journal.pgen.1006527

**Published:** 2016-12-29

**Authors:** The PLOS Genetics Staff

## Notice of Republication

This article was republished on December 6, 2016, to correct errors caused by the incorrect resizing of Fig 5 and Fig 7 during the typesetting process, and a correction has also been made in the Results section, under the sub-heading “Induction of effector gene expression mediated by *aTF1* overexpression is Sge1 dependent”. The sentence “Also, deletion of *SGE1* resulted in loss of pathogenicity in both WT and in *aTF1* overexpressing strains (S12 Fig).” has been moved from the end of the Results section to the end of the penultimate paragraph of the Results section.

The publisher apologizes for the errors. Please download this article again to view the correct version. The originally published, uncorrected article and the republished, corrected articles are provided here for reference.

## Supporting Information

S1 File Originally published, uncorrected article. (PDF)

S2 File Republished, corrected article. (PDF)

## References

[1] van der Does HC, Fokkens L, Yang A, Schmidt SM, Langereis L, Lukasiewicz JM, et al (2016) Transcription Factors Encoded on Core and Accessory Chromosomes of *Fusarium oxysporum* Induce Expression of Effector Genes. PLoS Genet 12(11): e1006401 doi:10.1371/journal.pgen.1006401 27855160 10.1371/journal.pgen.1006401 PMC5140021

