# Supplementary figures and images for: Transcription Factors Encoded on Core and Accessory Chromosomes of Fusarium oxysporum Induce Expression of Effector Genes
Source: PLoS Genet. 2016 Nov 17;12(11):e1006401. doi: 10.1371/journal.pgen.1006401 (PMC5140021; doi:10.1371/journal.pgen.1006401)

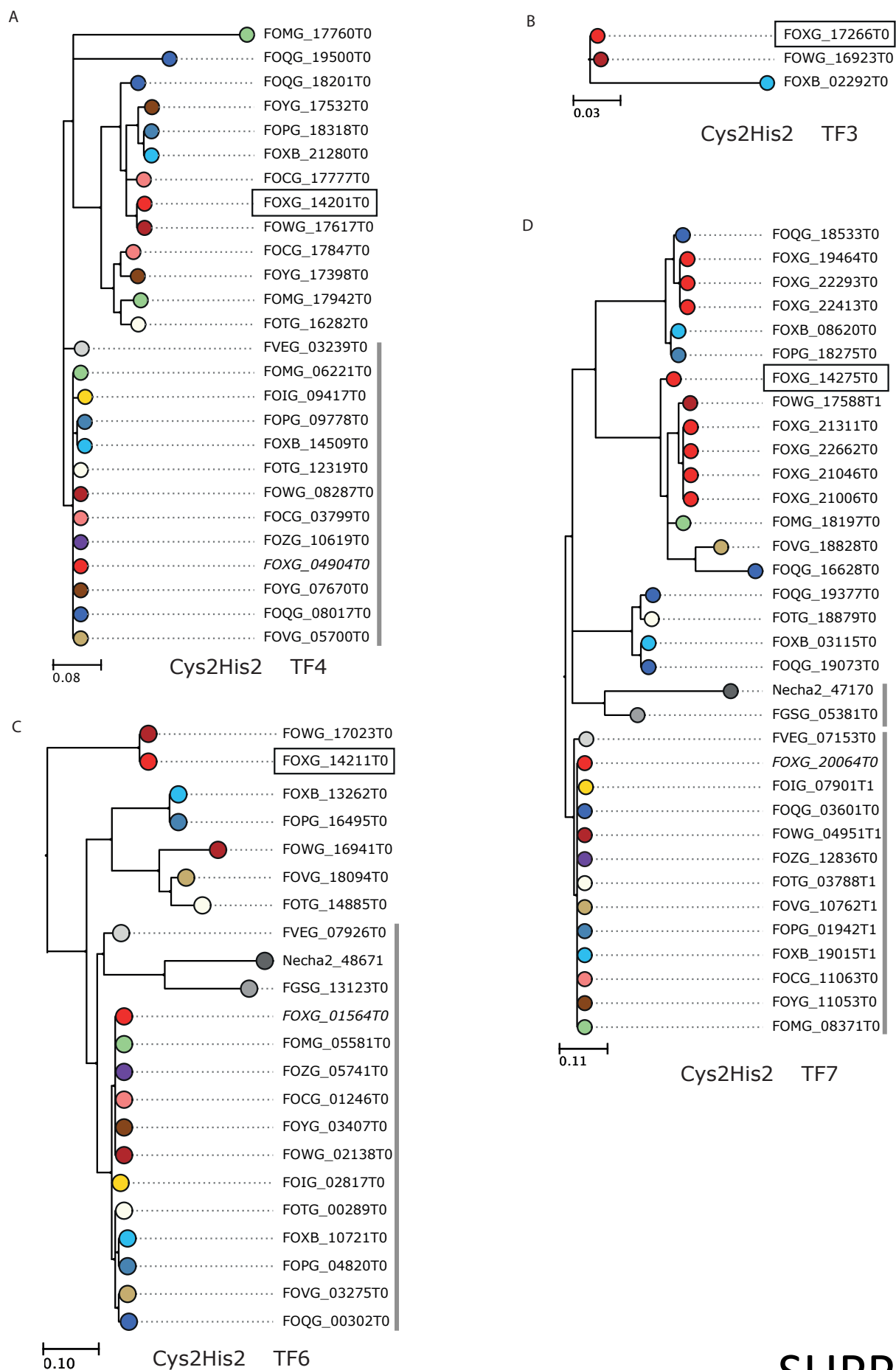

SUPP FIG 1

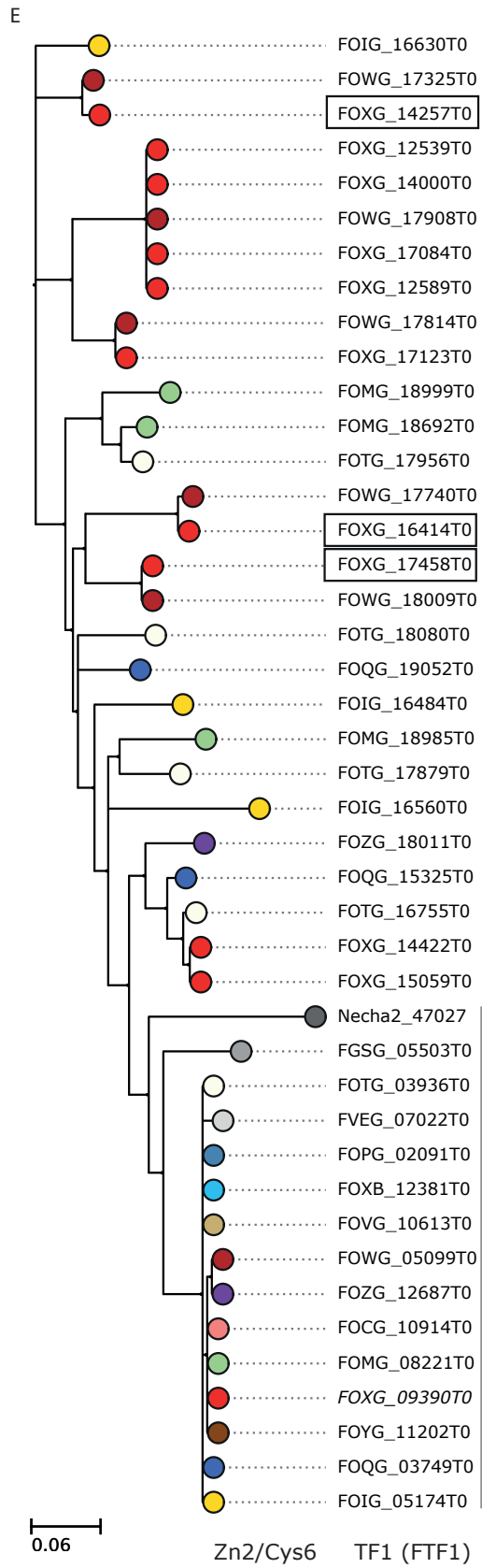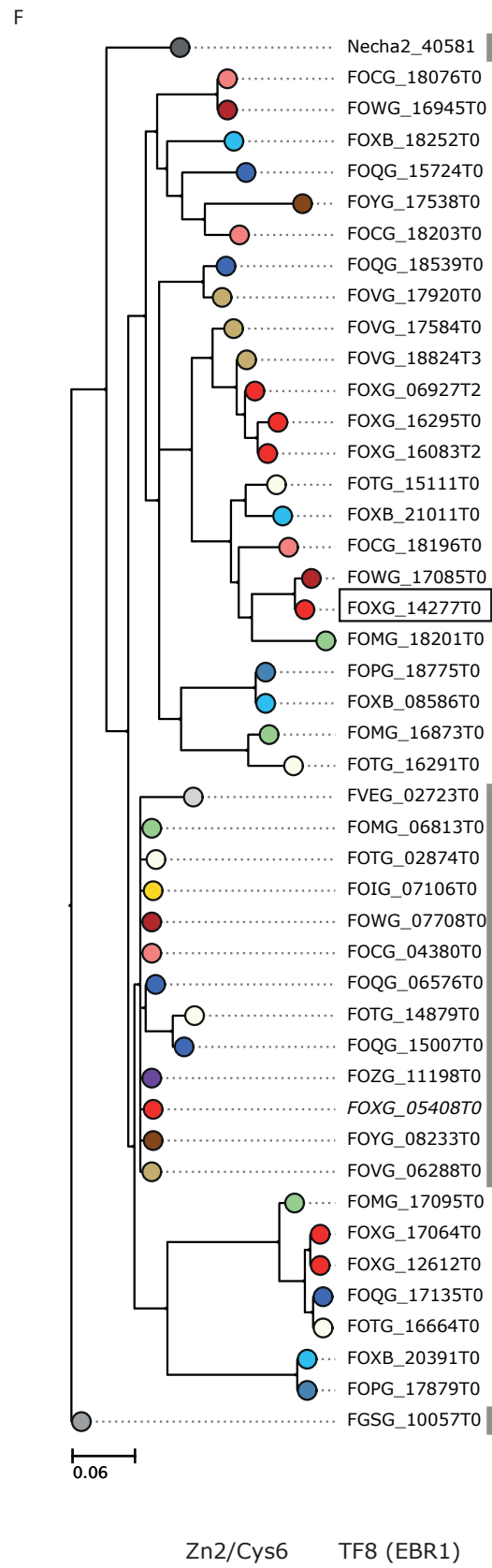

SUPP FIG 1

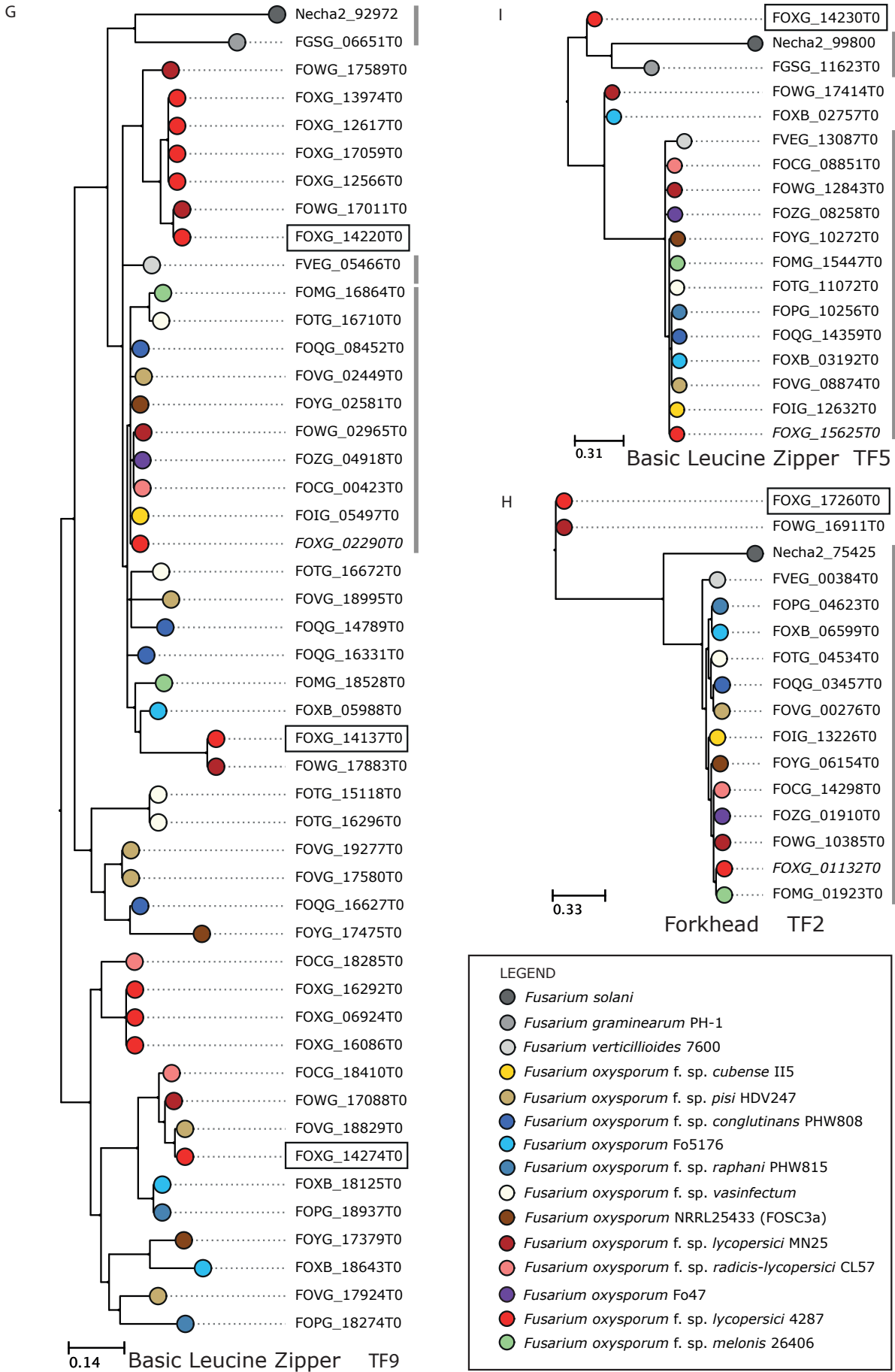

Supplement: S1 Fig — Phylogenetic trees of the protein sequences of the different transcription factor families, including homologs in Fol, other F. oxysporum ff. spp. and other Fusarium species. A) Family of aTf4 (FOXG_14201), a C2H2 zinc finger transcription factor—this transcription factor has no additional accessory homologs in Fol. B) aTf3 (FOXG_17266), a C2H2 zinc finger transcription factor with no homologs on the core genome, and only two homologs in accessory regions in other formae speciales. C) Family of aTf6 (FOXG_14211), a C2H2 zinc finger transcription factor—this transcription factor has no additional accessory homologs in Fol. D) Family of aTf7 (FOXG_14275), a C2H2 zinc finger transcription factor. This transcription factor has seven additional accessory homologs in Fol4287. E) Protein family of aTf1 (Ftf1; FOXG_14257, FOXG_17458, FOXG_16414) of Zn(2)Cys(6) zinc finger transcription factors. This transcription factor has seven additional accessory homologs in Fol4287. F) Family of aTf8 (FOXG_14277), a Zn(2)Cys(6) zinc finger transcription factor. This transcription factor has five additional accessory homologs in Fol4287. G) Family of aTf9 (FOXG_14274, FOXG_14137, FOXG_14220) of bZIP leucine zipper transcription factors. This family has seven additional accessory homologs in Fol4287. H) Family of aTf2 (FOXG_17260), a forkhead transcription factor with one homolog on the core genome and one homolog on the accessory chromosomes. Only three other forkhead transcription factor genes are present in the Fol4287 genome. I) Family of aTf5 (FOXG_14230), a bZIP leucine zipper transcription factor—this transcription factor has no additional accessory homologs in Fol. (PDF) [file pgen.1006401.s001.pdf]

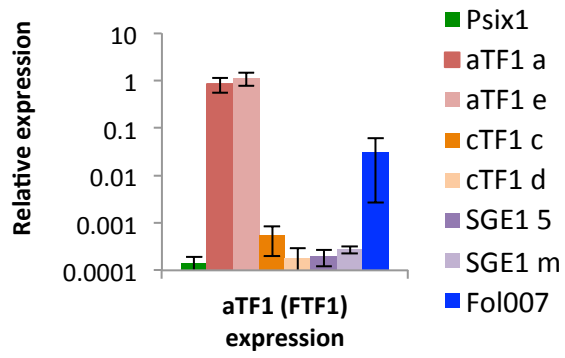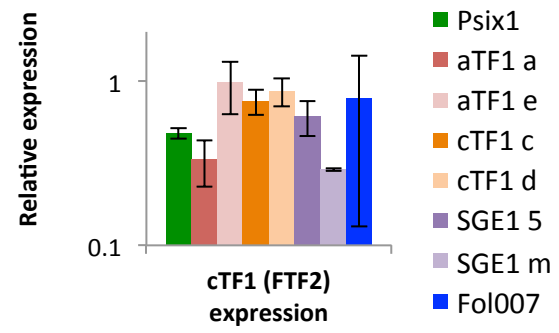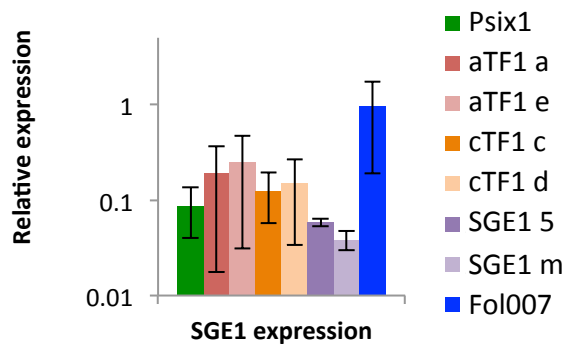

SUPP FIGURE 7

Supplement: S7 Fig — Relative expression was determined by Q-RT-PCR, and calculated as TF expression /expression of EF1α. Two independent transformants were tested per transcription factor. Plants were infected with strain Fol007 and roots were harvested for RNA extraction 9 dpi. Error bars indicate the standard error of two (during infection) or three (all other samples) independent biological replicates. (PDF) [file pgen.1006401.s007.pdf]

A

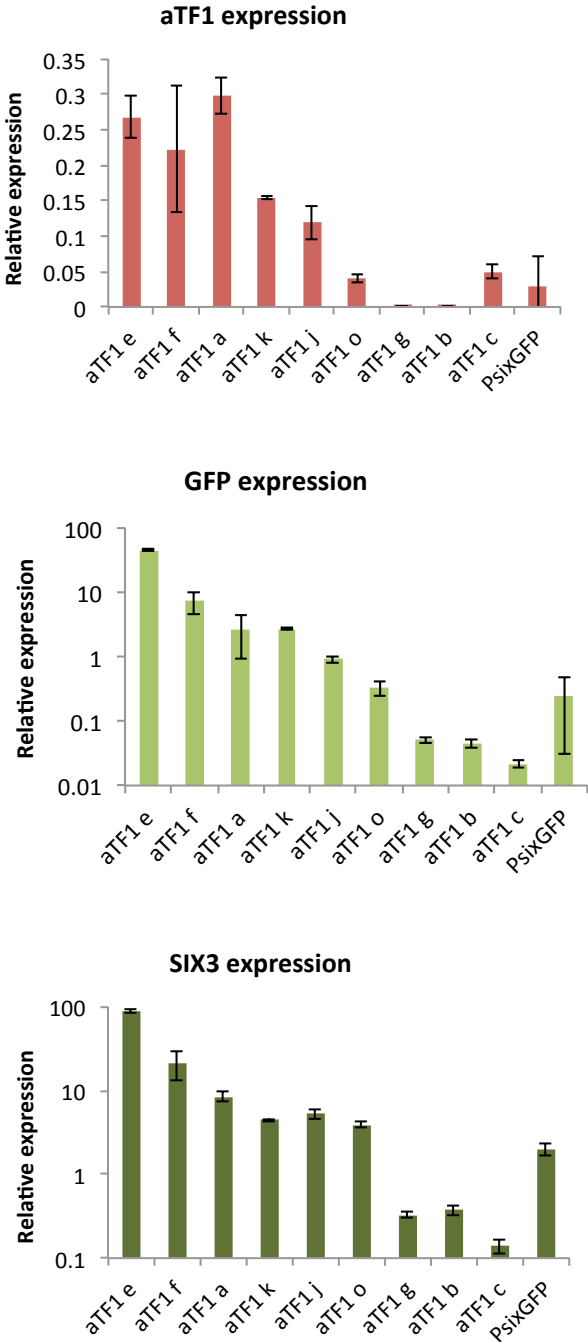

B

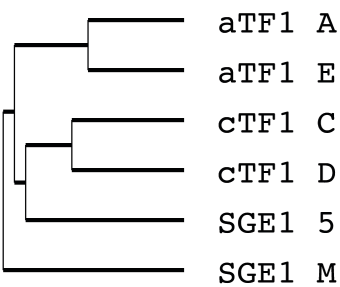

SUPP FIGURE 8

Supplement: S8 Fig — A) Relative expression of aTF1, GFP and SIX3 compared to expression of the reference gene EF1α determined by Q-RT-PCR. Expression was measured in nine different independent aTF1 overexpressors (a, b, c, e, f, g, j, k and o) and in the background strain (Psix1GFP). aTF1 is expressed from the endogenous locus and–in the overexpressors–from an additional overexpression construct. GFP is expressed by the SIX1 promoter, from the original locus. SIX1 and SIX3 are representative effector genes. Relative expression is calculated as gene expression/expression of EF1α. Error bars indicate standard deviation. B) clustering of differentially expressed genes (Padjusted < 0.01) after expression of aTF1, cTF1 or SGE1 from the FEM1 promoter, by log2 fold change values for each differentially expressed gene. The log2 fold change values of genes that are not significantly different from the control are set to zero. Two independent transformants were studied per transcription factor, resulting in six independent conditions. The tree reflects the similarity between the transcriptional changes in each sample. (PDF) [file pgen.1006401.s008.pdf]

SUPP FIG 8

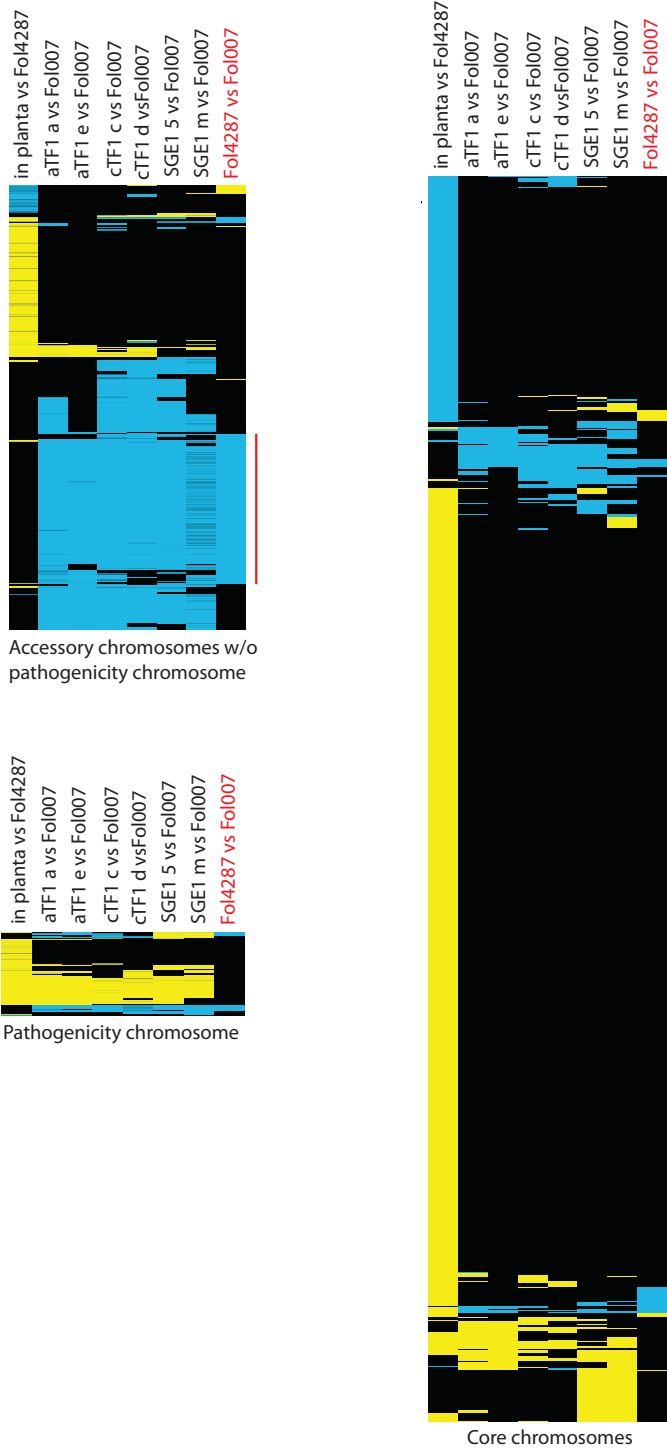

Supplement: S9 Fig — Heatmap of differentially expressed genes during infection or after in aTF1, cTF1 or SGE1 overexpressors, divided per subgenome (the pathogenicity chromosome, other accessory regions and core genome). Displayed is the log2 fold change for each differentially expressed gene (rows), with yellow indicating up-regulation compared to the control (hues cover the log2 fold range from 0 to 5). Blue indicates down-regulation compared to the control (hues cover the log2 fold range from 0 to -5). The log2 fold change values of genes that are not significantly different from the control are set to zero. Each condition is a separate column, with two independent transformants (thus 2 columns) per transcription factor. The order of the rows reflects clustering of similar expression patterns. The first seven columns are the same as in Fig 8A, the eighth column is additional (column header highlighted in red). Wild type strain Fol4287 was used for in planta samples, and for Fol4287 samples from flask (compared to each other in the first column). Fol007 is a wild type strain very similar to Fol4287 and was used to create the background strain (Fol007 + Psix1GFP reporter construct) for the overexpressors. Overexpressors and their background strain were compared in column two to seven. The eighth column compares the two wild type strains to each other (Fol4287 and Fol007+Psix1GFP). The red bar highlights the group of genes that is expressed lower in Fol 4287 WT and in the overexpressors in Fol 007 + Psix1GFP background, compared to Fol007 + Psix1GFP. (PDF) [file pgen.1006401.s009.pdf]

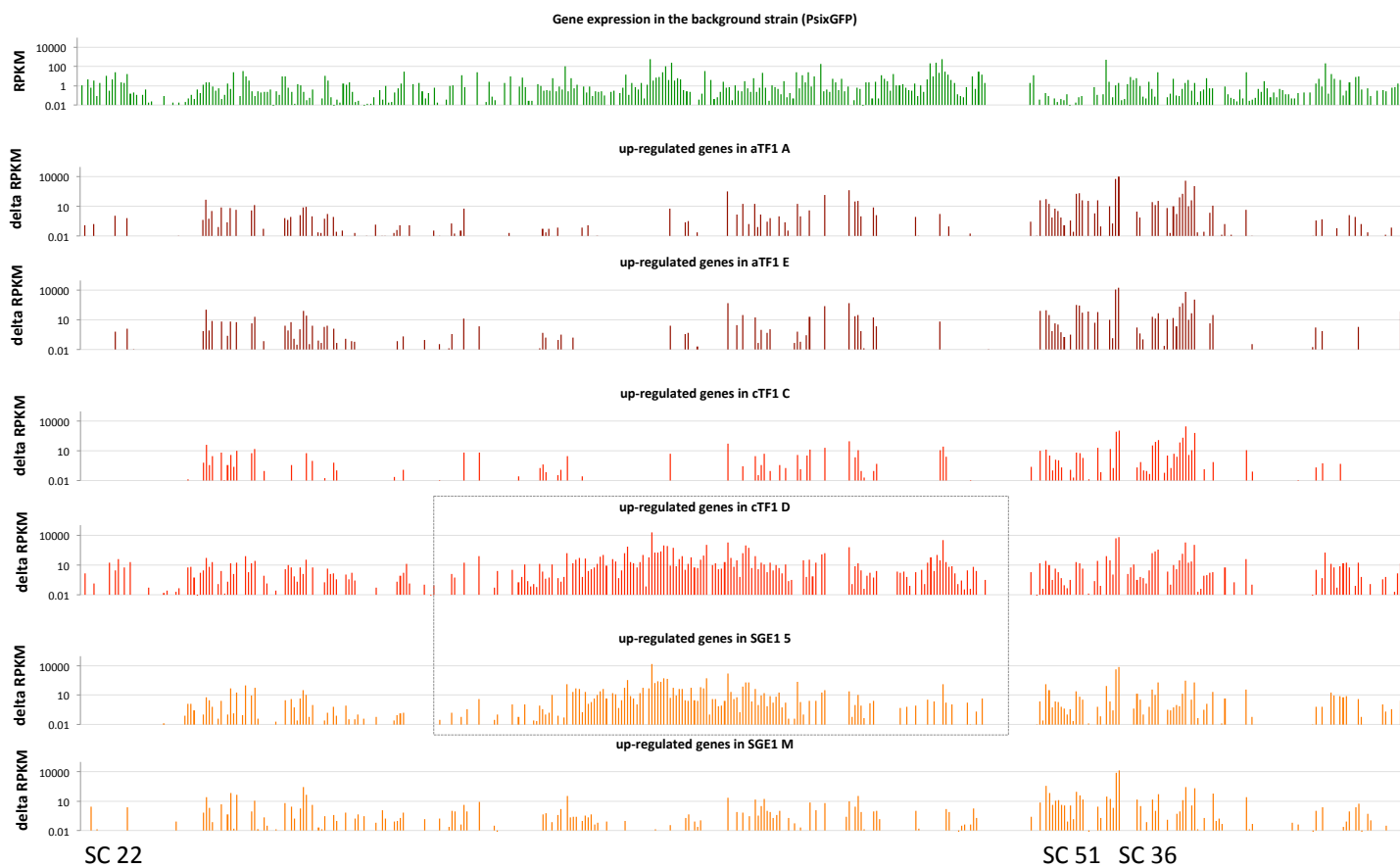

SUPP FIG 9

Supplement: S10 Fig — Expression (of the background strain, top row, green) or the difference in expression with the background strain (of the overexpressors, six bottom rows, different shades of red) in RPKM or ΔRPKM (log10 scale). On the x-axis, the genes of SC 22, SC 36 and SC 51 are plotted, in the same order as they appear on the supercontig (SC). Only positive numbers are shown for the overexpressors (ΔRPKM)–down-regulated genes are not visible. No distinction has been made between significant and non-significant up-regulation—all differences are shown. The second and third rows show two independent aTF1 overexpressors (dark red), the fourth and fifth rows show two independent cTF1 overexpressors (bright red), the sixth and seventh rows show two independent SGE1 overexpressors (orange). The dashed box indicates the region that is up-regulated in one cTF1 overexpressor and one SGE1 overexpressor. (PDF) [file pgen.1006401.s010.pdf]

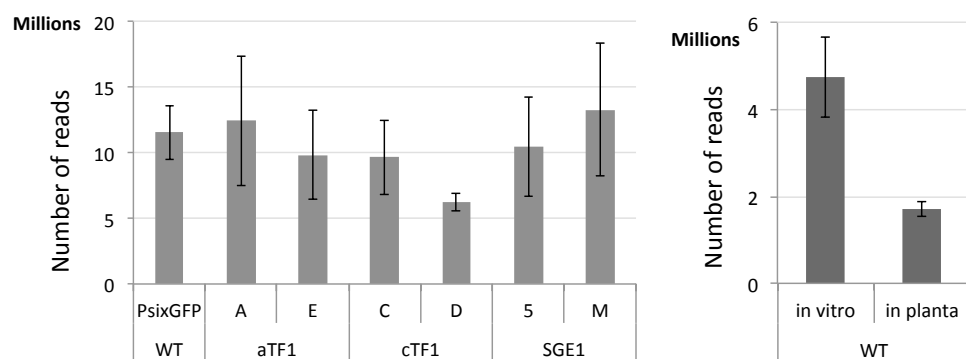

Total TE reads per 20 million reads uniquely mapped to the reference genome

Supplement: S11 Fig — All sequencing reads from the overexpressors and reads from infected plant material were mapped to a fasta file where the sequence of each repetitive element, plus all each previously annotated transposable element, is present once. The number of reads was normalized to the number of reads mapped uniquely to the whole genome for each sample. In the graph, the total number of reads assigned to transposable elements per 20 million reads uniquely mapped to the reference genome are shown. Of each transcription factor overexpressor (aTF1, cTF1 or SGE1) two independent transformants are shown. No consistent significant effects on overall transposon expression were observed in any of the overexpressors. However, the total amount of transposon-derived reads during infection was reduced. (PDF) [file pgen.1006401.s011.pdf]

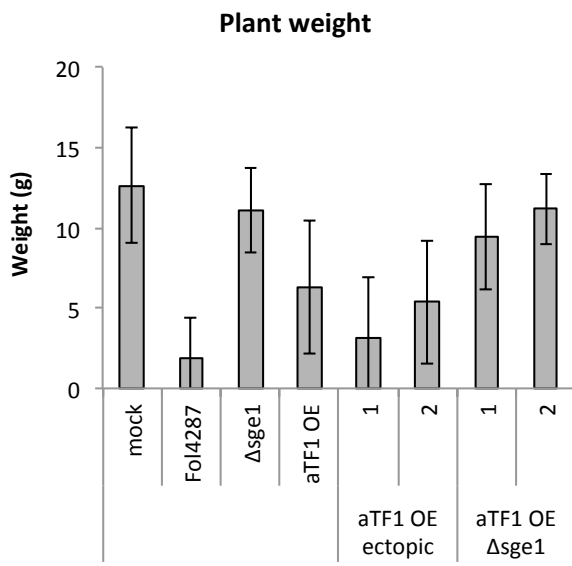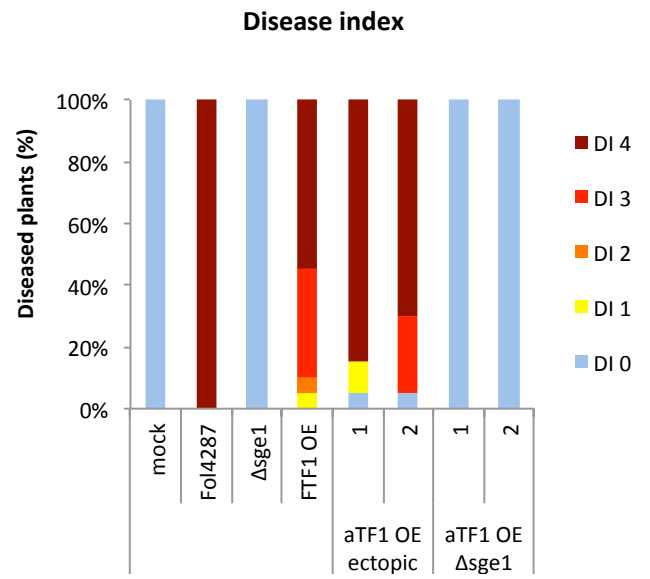

SUPP FIGURE 11

Supplement: S12 Fig — Bioassay on susceptible tomato plants. Two independent transformants were tested for both ectopic and in locus integration of the SGE1 deletion construct in the aTF1 overexpressor (aTF1 OE ectopic and aTF1 Δsge1, respectively). Control treatments were: mock, Fol4287 (WT), Δsge1 (SGE1 deletion mutant in Fol4287), aTF1 OE (aTF1 overexpressor in Fol4287). Left panel: average plant weight (gram) 21 dpi. Error bars indicate standard deviation. Right panel: disease index (0–4 arbitrary units) 21 dpi. (PDF) [file pgen.1006401.s012.pdf]
