# Supplementary material for: Transcription Factors Encoded on Core and Accessory Chromosomes of Fusarium oxysporum Induce Expression of Effector Genes
Source: PLoS Genet. 2016 Nov 17;12(11):e1006401. doi: 10.1371/journal.pgen.1006401 (PMC5140021; doi:10.1371/journal.pgen.1006401)
Supplement: S2 Fig — In-frame potential startcodons (ATG) are indicated with red arrows and are bold and underlined, other ATG codons are bold. Red bases indicate single basepair indels. The following genes have a ‘long’ predicted ORF, according to the Broad annotation (the number refers to the FOXG number of the Broad annotation): 09390, cTF1, core genome; 17458, pathogenicity chromosome, used for overexpression & RNAseq; 14257, pathogenicity chromosome, 14422, accessory chromosome 15; 15059, accessory region on chromosome 1. The remaining genes have a shorter predicted ORF according to the Broad annotation, namely: 16414, pathogenicity chromosome; 17123, accessory chromosome 6, 17084, accessory chromosome 3/6, used for overexpression; 14000, accessory chromosome 3/6; 12589, accessory chromosome 3/6, 12539, accessory chromosome 3/6. The genes with a long predicted ORF start at the ATG indicated with red arrow number 1. All remaining genes have a 17 base pair deletion (compared to the core homolog cTF1/>09390), removing this ATG. Their predicted startcodon is the next in-frame ATG (red arrow 2). In addition, all homologs with the 17 bp deletion also have a more upstream stop codon– 300 bp upstream of the stopcodon in cTF1. This means that all ORFs starting at the position indicated by red arrow 2, start 117 bp later and stop 300 bp earlier than the long TF1 homologs, rendering ORFs of around 2800 bp. These genes we refer to as having a short ORF. The ORFs starting at the position indicated by red arrow 1 are around 3200 bp. These genes we refer to as having a long ORF. (PDF) [file pgen.1006401.s002.pdf]

```
                                                    1
         1                                                                                                                                                         138
>09390 TTCTTGGCCTCGGAAGGCCCGCGTCGCAACTCTGTCTCTGGCAGAGCCATGTCCGGTCGTGCCGTTTC-GAATTCTCAACATGCCCAGCAGTCCTTCGGTTCCGGTCCTCAACTCTATCGCGACGTCGCTGAATTGCA
>17458 TTCTCGGCCTCGGGTGGCC-ACGTCGTAACTCTGTCTCTGGCAGAGCCATGTCCGGCCGTGCCGTTTT-GAGCCCTCAACATGCCCAAGCGTCCTTTGATTCTGGTCTTCAACTCTATGGCGACGTGCCTGAATTGAA
>14257 TTCTCGGCCTCGGGTGGCC-GCGTCGTAACTTTGTCTCTGGCAGAGCCATGTCCGGCCATGCCGTTTTTGAACCCTCAACATGCCCAATAGTCCTTCGATTCCGGTCC-CAACTGTATCGCGACGTTCCTGAATTGCA
>16414 TTCCCGGCCTCGGATGGCC-GCGTCGTAACTC-----------------CGTCCGACAGTGTCGTTTT-GAACCCTCGACATGCCCAAGAGTCCTTCGATTCCGGTAC-CAACTCTATCGCGACGTACCTGAATCTCA
>17084 TTCTCGGCCTCGGGTGGCC-GCGTCGTAACTC-----------------TGTCCGGCCGTGCCGTTTT-GAATCCTCAATATGCCCAACAGTCCTTCGATTCCGGTCCTCAACTCTATCGCGACGTTCCTGAATTGCA
>14000 TTCTCGGCCTCGGGTGGCC-GCGTCGTAACTC-----------------TGTCCGGCCGTGCCGTTTT-GAATCCTCAATATGCCCAACAGTCCTTCGATTCCGGTCCTCAACTCTATCGCGACGTTCCTGAATTGCA
>12589 TTCTCGGCCTCGGGTGGCC-GCGTCGTAACTC-----------------TGTCCGGCCGTGCCGTTTT-GAATCCTCAATATGCCCAACAGTCCTTCGATTCCGGTCCTCAACTCTATCGCGACGTTCCTGAATTGCA
>12539 TTCTCGGCCTCGGGTGGCC-GCGTCGTAACTC-----------------TGTCCGGCCGTGCCGTTTT-GAATCCTCAATATGCCCAACAGTCCTTCGATTCCGGTCCTCAACTCTATCGCGACGTTCCTGAATTGCA
>17123 TTCTCGGCCTCGGGTGGCC-GGGTCGTAACTC-----------------TGTCCGGCCGTGCCGTTTT-GAACCCTCAACATGCCCAACAGTTCTTCGATTCCGGTTCCCAACTCTATCGCGACGTTCCTGAATTGCA
>14422 TTCTCGGCCTCGGGTGGCCCGCGTCGCAACTCTGTCTCTGGCAGAGCCATGTCCGGCCGTGCCGTTTC-GAACCCTCAACATGCCCAACAGTCCTTCGATTCCGGTCCCCAACTCTATCGCGACGTTCCTGAATTGCA
>15059 TTCTCGGCCTCGGGTGGCCCGCGTCGCAACTCTGTCTCTGGCAGAGCCATGTCCGGCCGTGCCGTTTC-GAACCCTCAACATGCCCAACAGTCCTTCGATTCCGGTCCCCAACTCTATCGCGACGTTCCTGAATTGCA

                                        2
       139                                                                                                                                                         277
>09390 CGCTGTGCCATCTCCCTCTCACG-GCGCCTTA-ATGGATCCCTCACATTTTGACGATTTCGCGTTTGCTTACCAAGGTCTTCCTGACCAACCTTCTCTCGTTTCTCTGGCCGATCACGCCCACGCCCACGCCTCACAA
>17458 CGCTGTGCCTCCTCCCTCTCACA-GCGCCTTA-ATGGATTTCACACGCTTCGACGATTTTGCGTTTGCTTACTATGGTCTTCCTGACCAGTCTTCTCTCGTTTCCCTAGTAGATCACACCCACACG------TTCCAA
>14257 CGCTGTGCCTTCTCCCTCTCACA-GCGCCTTA-ATGGATTTCACACACTTCGACGATGTTGCGTTTGCCTACTATGGTCTTCCTGACCAGTCTTCTCTCGTTTCCCTACTAGATCACACCCACACG------TTCCAA
>16414 GGCTGTGCCTTTTCACTCTCACA-GCGCCTTA-ATGGATTTCACACAGTTGGACGATTTTGCGTTTGCTTACTATGGTCTTCCTGACCAGTCTTCTCTCGTTTCCCTAGTGGATCAAACCCACACG------TTCCAA
>17084 CGCTGTACCTCCTCCCTCTCACA-GCGTCCTA-ATGGATTTCACAAACTTCGACGATTTTGCGTTTGCTTACTATGGTCTTCCTGTCCGGGCTTCTCTCGTTTCCCTAGTAGATCACACCCACACG------TTCCAA
>14000 CGCTGTACCTCCTCCCTCTCACA-GCGTCCTA-ATGGATTTCACAAACTTCGACGATTTTGCGTTTGCTTACTATGGTCTTCCTGTCCGGGCTTCTCTCGTTTCCCTAGTAGATCACACCCACACG------TTCCAA
>12589 CGCTGTACCTCCTCCCTCTCACAAGCGTCCTAAATGGATTTCACAAACTTCGACGATTTTGCGTTTGCTTACTATGGTCTTCCTGTCCGGGCTTCTCTCGTTTCCCTAGTAGATCACACCCACACG------TTCCAA
>12539 CGCTGTACCTCCTCCCTCTCACA-GCGTCCTA-ATGGATTTCACAAACTTCGACGATTTTGCGTTTGCTTACTATGGTCTTCCTGTCCGGGCTTCTCTCGTTTCCCTAGTAGATCACACCCACACG------TTCCAA
>17123 CGCCGTGCCTTCTCCCCCTCACA-GCGCCTTA-ATGGATTTCACACACTTCGACGATTTTGCGTTTGCTTACTATGGTCTTCCTGACCAGGCTTCTCTCGCTTCCCTAGTAGATCACACCCACATG------TTCCAA
>14422 CGCTGTGCCTTCTCCCTCTCACA-GCGCCTTA-ATGGACTCCACACACTTCGACGATTTTGCGTTTGCTTACCATGGTCTTCCTGACCAGTCTTCTCTAGTTTCCCTAGCAGATCACACCCACACG------TCACAA
>15059 CGCTGTGCCTTCTCCCTCTCACA-GCGCCTTA-ATGGACTCCACACACTTCGACGATTTTGCGTTTGCTTACCATGGTCTTCCTGACCAGTCTTCTCTAGTTTCCCTAGCAGATCACACCCACACG------TCACAA
```

SUPP FIG 2
