# Supplementary material for: Transcription Factors Encoded on Core and Accessory Chromosomes of Fusarium oxysporum Induce Expression of Effector Genes
Source: PLoS Genet. 2016 Nov 17;12(11):e1006401. doi: 10.1371/journal.pgen.1006401 (PMC5140021; doi:10.1371/journal.pgen.1006401)
Supplement: S3 Fig — For each family we predicted domain architecture for its members. We here show for each family, from the multiple sequence alignment used to generate its phylogeny (S1 Fig), those regions that correspond to a DNA binding domain. The background color of a residue corresponds to its conservation in the alignment, where darker colors indicate more conservation. Residues that are conserved in the seed alignment from Pfam and are present in at least two sequence are indicated in bold on top of the alignment. Residues that are conserved in the Pfam seed alignment but are not present in at least two sequences are in normal font. Gene names correspond to names and species or ff. spp. mentioned in Fig 2and S1 Fig. Core-encoded transcription factors of F. oxysporum (Fol4287), F. verticillioides, F. graminearum and F. solani are indicated in red. Accessory transcription factors from Fol tested for DNA binding specificity are indicated in bold. (PDF) [file pgen.1006401.s003.pdf]

TF1: Zn2/Cys6 (Pfam: Zn_clus)

```
                      C  Cr kK  C     P C  C      C
FOIG_16630T0   175  LACIACRRKKVRCSGEKPACKHCLHSHTPCVYKVTTRKAAPRTDYMAMLDKRPKCMEERI
FOWG_17325T0   175  LACIACRRKKVRCSGEKPACKHCLHSHIPCVYKVTTRKAAPRTNYMVMLDKRPKCMEERI
FOXG_14257T0   135  LACIACRRKKVRCSGEKPACKHCLHSHIPCVYKVTTRKAAPRTNYMVMLDKRPKCMEERI
FOXG_12539T0   135  LACVACRRKKSRCSGEKPACEHCLRSYIPCVYKVTTRKAAPRTNCMAMLDKRPKRMEERV
FOXG_17084T0   135  LACVACRRKKSRCSGEKPACEHCLRSYIPCVYKVTTRKAAPRTNCMAMLDKRPKRMEERV
FOXG_12589T0   135  LACVACRRKKSRCSGEKPACEHCLRSYIPCVYKVTTRKAAPRTNCMAMLDKRPKRMEERV
FOXG_14000T0   135  LACVACRRKKSRCSGEKPACEHCLRSYIPCVYKVTTRKAAPRTNCMAMLDKRPKRMEERV
FOWG_17908T0   135  LACVACRRKKSRCSGEKPACEHCLRSYIPCVYKVTTRKAAPRTNCMAMLDKRPKRMEERV
FOWG_17814T0   135  LACIPCRRKKIRCSGEKPACEHCLRSYIPCVYKITTRKAAPRTDYMAMLDKRPKCMEERV
FOXG_17123T0   135  LACIPCRRKKIRCSGEKPACEHCLRSYIPCVYKITTRKAAPRTDYMAMLDKRPKCMEERV
FOMG_18999T0   135  LACIACRQKKTRCSGEKPACKHCLRSYIPCVYKVTTWKAAPRTNYMAMFDKRLKRMEERI
FOMG_18692T0   175  LACIACRRMKIRCSGEKPACKHCLHSYIPCVYKVTIRKAAPRTNHMTMLDKRPKRMEERI
FOTG_17956T0   175  LACIACRRMKIRCSGEKPACKHCLHSYIPCVYKVTIRKAAPRTNYMTMLDKRPKRMEERI
FOWG_17740T0   135  LACIACRRKKIRCSGEKPACEHCLCSYIPCVYKVTTRKAGPRTDYMAMLDKRPKRMEERV
FOXG_16414T0   135  LACIACRRKKIRCSGEKPACEHCLCSYIPCVYKVTTRKAGPRTDYMAMLDKRPKRMEERV
FOXG_17458T0   175  LACIACRRKKIRCSGEKPACKQCLHSCIPCVYKVATRKAAPRTNCMAMLDKRPKRMEERA
FOWG_18009T0   175  LACIACRRKKIRCSGEKPACKQCLHSCIPCVYKVATRKAAPRTNCMAMLDKRPKRMEERA
FOTG_18080T0   175  LACIACRQKKIRCSGEKPACKQCLLSCIPCVYKVATRKAAPRTNCMAMLDKRPKRMEER-
FOQG_19052T0   175  LACIACRRKKIRCSGDKPACKHCLHSYIPCVYKVTTRKAAPRTNHMTMPNKLPKRMEERI
FOIG_16484T0   175  LACIAYRRKKIRCSGEKPACKDCLRSCIPCVYKVTTRKAAPRTDCMAMLDKRPKRMEERV
FOMG_18985T0   175  LACIACRRKKIRCSGEKPACEDCLRSYIPCVYKVTTRKAAPRADCMAMLDKRPKRMEERI
FOTG_17879T0   175  LACIACRRKKIRCSGEQPACKQCLRSCIPCVYKVATRKAAPQTDCMALLDKRPKRMEERI
FOIG_16560T0   175  LACIACRRMKIGCSGEKPTCKHCLRSCIPCVYKGTTRKAAPRTDYMAMLDKRPKRMEERV
FOZG_18011T0   135  LACMACRRKKIRCSGEKPACKHCLHSRIPCVYKVTTRKAAPQTGYMAMLDKPLKRMEERI
FOQG_15325T0   175  LACMACRRKKIRCSGEKPACKHCLQSRIPCVYKVTTRKAAPRTGYMAMLDKPLKRMEERI
FOTG_16755T0   176  LACMACRRKKIRCSGEKPACKHCLRSRIPCVYKVTTRKAAPRTGYMAMLDKPLKRMEERI
FOXG_14422T0   176  LACMACRRKKIRCSGEKPACKHCLRSRILCVYKVTTRKAAPRTGYIAMLDKPLKRMEERI
FOXG_15059T0   176  LACMACRRKKIRCSGEKPACKHCLRSRILCVYKVTTRKAAPRTGYIAMLDKPLKRMEERI
Necha2_47027   174  LACIACRRKKIRCSGEKPACKHCLRSRIPCVYKVTTRKAAPRTDYMAMLDKRLKRMEERI
FGSG_05503T0   175  LACIACRRKKIRCSGEKPACKHCLRSRIPCVYKVTTRKAAPRTDYMAMLDKRLKRMEERI
FOTG_03936T0   179  LACIACRRKKIRCSGEKPACKHCLRSRIPCVYKVTTRKAAPRTDYMAMLDKRLKRMEERI
FVEG_07022T0   177  LACIACRRKKIRCSGEKPACKHCLRSRIPCVYKVTTRKAAPRTDYMAMLDKRLKRMEERI
FOPG_02091T0   177  LACIACRRKKIRCSGEKPACKHCLRSRIPCVYKVTTRKAAPRTDYMAMLDKRLKRMEERI
FOVG_10613T0   177  LACIACRRKKIRCSGEKPACKHCLRSRIPCVYKVTTRKAAPRTDYMAMLDKRLKRMEERI
FOWG_05099T0   177  LACIACRRKKIRCSGEKPACKHCLRSRIPCVYKVTTRKAAPRTDYMAMLDKRLKRMEERI
FOZG_12687T0   177  LACIACRRKKIRCSGEKPACKHCLRSRIPCVYKVTTRKAAPRTDYMAMLDKRLKRMEERI
FOCG_10914T0   177  LACIACRRKKIRCSGEKPACKHCLRSRIPCVYKVTTRKAAPRTDYMAMLDKRLKRMEERI
FOMG_08221T0   177  LACIACRRKKIRCSGEKPACKHCLRSRIPCVYKVTTRKAAPRTDYMAMLDKRLKRMEERI
FOXG_09390T0   177  LACIACRRKKIRCSGEKPACKHCLRSRIPCVYKVTTRKAAPRTDYMAMLDKRLKRMEERI
FOYG_11202T0   177  LACIACRRKKIRCSGEKPACKHCLRSRIPCVYKVTTRKAAPRTDYMAMLDKRLKRMEERI
FOXB_12381T0   177  LACIACRRKKIRCSGEKPACKHCLRSRIPCVYKVTTRKAAPRTDYMAMLDKRLKRMEERI
FOQG_03749T0   177  LACIACRRKKIRCSGEKPACKHCLRSRIPCVYKVTTRKAAPRTDYMAMLDKRLKRMEERI
FOIG_05174T0   177  LACIACRRKKIRCSGEKPACKHCLRSRIPCVYKVTTRKAAPRTDYMAMLDKRLKRMEERI
```

SUPP FIG 3

TF2: Forkhead domain (Pfam: Forkhead)

```
                                                      PK    Y  LI  AI   P    T
FOXG_17260T0  123 NMKTW-QEPPTGEPEYCANSRVKSEPLELKS-TAVAPKAKEPYAKLIYRALMSAPDQAMT
FOWG_16911T0  123 NMKTW-QEPPTGEPEYCANSRVKSEPLELKS-TAVAPKAKEPYAKLIYRALMSAPDQAMT
Necha2_75425  239 YMTTKKEESPAVEPENRASPDKQPETSESQSSSAVGAKPEEPYAQLIYRALMGAPGHAMT
FVEG_00384T0  233 LMKAR-HESPAGVPEYRASSEVVQEGKDLQATAAGGAKTDEPYAKLLYRALMSVPEHAMT
FOPG_04623T0  233 LMKAR-HQSPAGVPEYRANSEVVQEGKDLQAAAAGGAKTDEPYAKLLYRALMSVPDHAMT
FOXB_06599T0  233 LMKAR-HQSPAGVPEYRANSEVVQEGKDLQAAAAGGAKTDEPYAKLLYRALMSVPDHAMT
FOTG_04534T0  233 LMKAR-HESPAGVPEYRANSEVVQEGKDLQAAAAGGAKTDEPYAKLLYRALMSVPDHAMT
FOQG_03457T0  234 LMKAR-HESPAGVPEYRANSEVVQEGKDLQAAAAGGAKTDEPYAKLLYRALMSVPDHAMT
FOVG_00276T0  233 LMKAR-HESPAGVPEYRANSEVVQEGKDLQAAAAGGAKTDEPYAKLLYRALMSVPDHAMT
FOIG_13226T0  233 LMKAR-HKSPAGVPEYRANSEVVQEGKDLQAAAAGGAKTDEPYAKLLYRALMSVPDHAMT
FOYG_06154T0  233 LMKAR-HKSPAGVPEYRANSEVVQEGKDLQAAAAGGAKTDEPYAKLLYRALMSVPDHAMT
FOCG_14298T0  233 LMKAR-HKSPAGVPEYRANSEVVQEGKDLQAAAAGGAKTDEPYAKLLYRALMSVPDHAMT
FOZG_01910T0  233 LMKAR-HKSPAGVPEYRANSEVVQEGKDLQAAAAGGAKTDEPYAKLLYRALMSVPDHAMT
FOWG_10385T0  233 LMKAR-HKSPAGVPEYRANSEVVQEGKDLQAAAAGGAKTDEPYAKLLYRALMSVPDHAMT
FOXG_01132T0  233 LMKAR-HKSPAGVPEYRANSEVVQEGKDLQAAAAGGAKTDEPYAKLLYRALMSVPDHAMT
FOMG_01923T0  233 LMKAR-HKSPAGVPEYRANSEVVQEGKDLQAAAAGGAKTDEPYAKLLYRALMSVPDHAMT

                  L                             WQNSIRHNLS  N  F K
FOXG_17260T0  181 LHEIYQWFRDNTDKHIKKDSTRRPGKNDNGWQNSIRHNLSMNEAFVKREYKQLSDPASN
FOWG_16911T0  181 LHEIYQWFRDNTDKHIKKDSTRRPGKNDNGWQNSIRHNLSMNEAFVKREYKQLSDPASN
Necha2_75425  299 LQEIYQWFRDNTDKESKNENTGKKSGKNAEGWQNSIRHNLSMNKAFTKDPPKKEPGSGPS
FVEG_00384T0  292 LQEIYQWFRDNTDKDIKKDKSEKRPGKNAEGWQNSIRHNLSMNDAFVKREHKLGSDSASR
FOPG_04623T0  292 LQEIYQWFRENTDKDIKKDKSEKRPGKNAEGWQNSIRHNLSMNDAFVKREHKLASDSASR
FOXB_06599T0  292 LQEIYQWFRENTDKDIKKDKSEKRPGKNAEGWQNSIRHNLSMNDAFVKREHKLASDSASR
FOTG_04534T0  292 LQEIYQWFRENTDKDIKKDKSEKRPGKNAEGWQNSIRHNLSMNDAFVKREHKLASDSASR
FOQG_03457T0  293 LQEIYQWFRENTDKDIKKDKSEKRPGKNAEGWQNSIRHNLSMNDAFVKREHKLASDSASR
FOVG_00276T0  292 LQEIYQWFRENTDKDIKKDKSEKRPGKNAEGWQNSIRHNLSMNDAFVKREHKLASDSASR
FOIG_13226T0  292 LQEIYQWFRENTDKDIKKDKSEKRPGKNAEGWQNSIRHNLSMNDAFVKREHKLASDSASR
FOYG_06154T0  292 LQEIYQWFRENTDKDIKKDKSEKRPGKNAEGWQNSIRHNLSMNDAFVKREHKLASDSASR
FOCG_14298T0  292 LQEIYQWFRENTDKDIKKDKSEKRPGKNAEGWQNSIRHNLSMNDAFVKREHKLASDSASR
FOZG_01910T0  292 LQEIYQWFRENTDKDIKKDKSEKRPGKNAEGWQNSIRHNLSMNDAFVKREHKLASDSASR
FOWG_10385T0  292 LQEIYQWFRENTDKDIKKDKSEKRPGKNAEGWQNSIRHNLSMNDAFVKREHKLASDSASR
FOXG_01132T0  292 LQEIYQWFRENTDKDIKKDKSEKRPGKNAEGWQNSIRHNLSMNDAFVKREHKLASDSASR
FOMG_01923T0  292 LQEIYQWFRENTDKDIKKDKSEKRPGKNAEGWQNSIRHNLSMNDAFVKREHKLASDSASR

FOXG_17260T0  241 GG--DHGSTKDDESKKPTKWTLESWAVQDGVKSTRRYRHNKYGVLR--------------
FOWG_16911T0  241 GG--DHGSTKDDESKKPTKWTLESWAVQDGVKSTRRYRHNKYGVLR--------------
Necha2_75425  359 MT----ESTAGGDPKRSTEWRLVDWAIRDGVQSTTRYRPGNSARRAIGTKVQHHPYNHHG
FVEG_00384T0  352 ATEQGSGSTKTGEAKKATEWVLEDWAVLNGVESTTKYRGKNTSRRGMGRKYHHHPYDY--
FOPG_04623T0  352 ATEQGSGSTKTGEAKKATEWVLEDWAVLNGVESTTKYRGKNTSRRGMGRKYHHHPYDD--
FOXB_06599T0  352 ATEQGSGSTKTGEAKKATEWVLEDWAVLNGVESTTKYRGKNTSRRGMGRKYHHHPYDD--
FOTG_04534T0  352 ATEQGSGSTKTGEAKKATEWVLEDWAVLNGVESTTKYRGKNTSRRGMGRKYHHHPYDD--
FOQG_03457T0  353 ATEQGSGSTKTGEAKKATEWVLEDWAVLNGVESTTKYRGKNTSRRGMGRKYHHHPYDD--
FOVG_00276T0  352 ATEQGSGSTKTGEAKKATEWVLEDWAVLNGVESTTKYRGKNTSRRGMGRKYHHHPYDD--
FOIG_13226T0  352 ATEQGSGSTKTGEAKKATEWVLEDWAVLNGVESTTKYRGKNTSRRGMGRKYHHHPYDD--
FOYG_06154T0  352 GTEQGSGSTKTGEAKKATEWVLEDWAVLNGVESTTKYRGKNTSRRGMGRKYHHHPYDD--
FOCG_14298T0  352 ATEQGSGSTKTGEAKKATEWVLEDWAVLNGVESTTKYRGKNTSRRGMGRKYHHHPYDD--
FOZG_01910T0  352 ATEQGSGSTKTGEAKKATEWVLEDWAVLNGVESTTKYRGKNTSRRGMGRKYHHHPYDD--
FOWG_10385T0  352 ATEQGSGSTKTGEAKKATEWVLEDWAVLNGVESTTKYRGKNTSRRGMGRKYHHHPYDD--
FOXG_01132T0  352 ATEQGSGSTKTGEAKKATEWVLEDWAVLNGVESTTKYRGKNTSRRGMGRKYHHHPYDD--
FOMG_01923T0  352 ATEQGSGSTKTGEAKKATEWVLEDWAVLNGVESTTKYRGKNTSRRGMGRKYHHHPYDD--
```

TF3: Cys2His2 domain (Pfam: zf-C2H2)

```
                    C    C   F     L  H   H
FOXG_17266T0 181 YKCEWPGCEKTFTQKKSLRVHAKTCRFQFPIFVPSDTISGLGVEGWHDSATGRDEVPSPA
FOWG_16923T0  95 YKCEWPGCEKTFTQKKSLRVHAKTCRFQFPIFVPSDTISGLGVEGWHDSATGRDEVPSPA
FOXB_02292T0 181 YKCKWPDCEKTFTQKKSLRVHAKTCRFQFPIFVPSDTISELGAEVWHDSATGRDGVPSPA

                                           C    C   F     L  H   H
FOXG_17266T0 481 CPLEAKKEAQYKTHLRTHTGERPFKCRREGCGKSFARPDSLKRHKKTCRHRKQEVLSLPQ
FOWG_16923T0 395 CPLEAKKEAQYKTHLRTHTGERPFKCRREGCGKSFARPDSLKRHKKTCRHRKQEVLSLPQ
FOXB_02292T0 481 CSLEAK---------------------------TFARQDSLKRHEKTCRHR---------
```

SUPP FIG 3

TF4: Cys2His2 domain (Pfam: zf-C2H2_4)

```
                                                         C  C               H    H
FOMG_17760T0 357 KPGRTKVLGFFMCECCLKKPKRFETPEELSAHEAEKQYECSFCGNRFKKKNEVERHRNSLH
FOQG_19500T0 343 KPGGTKVQGFFMCECCPKKPRRFETLEELK------------------------------
FOQG_18201T0 388 KPGGTTVQRFFMCECCPKRPKRFETVEELNAHEAEKRYECSFCGNRFKNKNEAERHKNSLH
FOYG_17532T0 388 KPGGTTVQRFFMCECCPKRPKRFETLEELNAHEAKKRYECSFCGSRFKNKNEAERHKNSLH
FOPG_18318T0 383 KPGGTTVQRFFMCECCPKRPKRFETLEELNAHEAERRYECSFCGNRFKNKNEAERHKNSLH
FOXB_21280T0 383 KPGGTTVQRFFMCECCPKRPKRFETLEELNAHEAERRYECSFCGNRFKNKNEAERHKNSLH
FOCG_17777T0 388 KPGGTTVQRFFMCECCPKRPKRFETLEELNAHEAKKRYECSFCGSRFKNKNEAERHKNSLH
FOXG_14201T0 388 KPGGTTVQRFFMCECCPKRPKRFETLEELNAHEAERRYECSFCGNRFKNKNEAERHKNSLH
FOWG_17617T0 388 KPGGTTVQRFFMCECCPKRPKRFETLEELNAHEAERRYECSFCGNRFKNKNEAERHKNSLH
FOCG_17847T0 388 KPGGTKVQRFFMCECCPKRPKRFETLEELNAHEAEKRYECSFCGNRFKNKNEAERHQNSLH
FOYG_17398T0 388 KPGGTKVQRFFMCECCPKRPKRFETLEELNAHEAEKRYECSFCGNRFKNKNEAERYQNSLH
FOMG_17942T0 388 KPGGTKVQRFFMCECCPKRPKRFETLEELNAHEAEKRYECSFCESRFKNKNEAERHQNSLH
FOTG_16282T0 388 KPGGTKVQRFFMCECCPKRPKRFETLEELNAHEAEKKYVCSFCGNRFKNKNEAERHQNSLH
FVEG_03239T0 436 KPGGTKVQGFFMCECCPKKPKRFETIEELNAHEAEKQYECSFCGNRFKNKNEAERHQNSLH
FOMG_06221T0 436 KPGGTKVQGFFMCECCPKKPKRFETIEELNAHEAEKQYECSFCGNRFKNKNEAERHQNSLH
FOIG_09417T0 436 KPGGTKVQGFFMCECCPKKPKRFETIEELNAHEAEKQYECSFCGNRFKNKNEAERHQNSLH
FOPG_09778T0 436 KPGGTKVQGFFMCECCPKKPKRFETIEELNAHEAEKQYECSFCGNRFKNKNEAERHQNSLH
FOXB_14509T0 436 KPGGTKVQGFFMCECCPKKPKRFETIEELNAHEAEKQYECSFCGNRFKNKNEAERHQNSLH
FOTG_12319T0 436 KPGGTKVQGFFMCECCPKKPKRFETIEELNAHEAEKQYECSFCGNRFKNKNEAERHQNSLH
FOWG_08287T0 436 KPGGTKVQGFFMCECCPKKPKRFETIEELNAHEAEKQYECSFCGNRFKNKNEAERHQNSLH
FOCG_03799T0 436 KPGGTKVQGFFMCECCPKKPKRFETIEELNAHEAEKQYECSFCGNRFKNKNEAERHQNSLH
FOZG_10619T0 436 KPGGTKVQGFFMCECCPKKPKRFETIEELNAHEAEKQYECSFCGNRFKNKNEAERHQNSLH
FOXG_04904T0 436 KPGGTKVQGFFMCECCPKKPKRFETIEELNAHEAEKQYECSFCGNRFKNKNEAERHQNSLH
FOYG_07670T0 436 KPGGTKVQGFFMCECCPKKPKRFETIEELNAHEAEKQYECSFCGNRFKNKNEAERHQNSLH
FOQG_08017T0 436 KPGGTKVQGFFMCECCPKKPKRFETIEELNAHEAEKQYECSFCGNRFKNKNEAERHQNSLH
FOVG_05700T0 436 KPGGTKVQGFFMCECCPKKPKRFETIEELNAHEAEKQYECSFCGNRFKNKNEAERHQNSLH
```

TF5: Basic Leucine Zipper domain (Pfam: bZIP_1, bZIP_2 and bZIP_Maf)

```
                                             R    NR AA       RK        L      L
FOXG_14230T0  108 --------KRAKTEDEKEQRRVERVLRNRRAAQSSRERKRQEVEALEKRNQELKAAFKQA
Necha2_99800   97 HGPHTASRKRAKTEDEKEQRRVERVLRNRRAAQSSRERKRLESEALMKRNKELENQLGEL
FGSG_11623T0  117 ESTDTLNRKRAKTEDEKEQRRVERVLRNRRAAQSSRERKRQEVEALEKRNQELEEAFMAA
FOWG_17414T0  108 --------KRAKTEDEKEQRRVERVLRNRRAAQSSRERKRQEVEALEKRNQELKAAFKQA
FOXB_02757T0  108 --------KRAKTEDEKEQRRVERVLRNRRAAQSSRERKRQEVEALEKRNQELEAAFKQA
FVEG_13087T0  105 --------KRAKTADEKEQRRVERVLRNRRAAQSSRERKRLEVEQLEQKNKDLEAAIQQA
FOCG_08851T0  104 --------KRAKTADEKEQRRVERVLRNRRAAQSSRERKRQEVEQLEKRNKDLEAAIQQA
FOWG_12843T0  104 --------KRAKTADEKEQRRVERVLRNRRAAQSSRERKRQEVEQLEKRNKDLEAAIQQA
FOZG_08258T0  104 --------KRAKTADEKEQRRVERVLRNRRAAQSSRERKRQEVEQLEKRNKDLEAAIQQA
FOYG_10272T0  104 --------KRAKTADEKEQRRVERVLRNRRAAQSSRERKRQEVEQLEKRNKDLEAAIQQA
FOMG_15447T0  104 --------KRAKTADEKEQRRVERVLRNRRAAQSSRERKRQEVEQLEKRNKDLEAAIQQA
FOTG_11072T0  104 --------KRAKTADEKEQRRVERVLRNRRAAQSSRERKRQEVEQLEKRNKDLEAAIQQA
FOPG_10256T0  104 --------KRAKTADEKEQRRVERVLRNRRAAQSSRERKRQEVEQLEKRNKDLEAAIQQA
FOQG_14359T0  104 --------KRAKTADEKEQRRVERVLRNRRAAQSSRERKRQEVEQLEKRNKDLEAAIQQA
FOXB_03192T0  104 --------KRAKTADEKEQRRVERVLRNRRAAQSSRERKRQEVEQLEKRNKDLEAAIQQA
FOVG_08874T0  104 --------KRAKTADEKEQRRVERVLRNRRAAQSSRERKRQEVEQLEKRNKDLEAAIQQA
FOIG_12632T0  104 --------KRAKTADEKEQRRVERVLRNRRAAQSSRERKRQEVEQLEKRNKDLEAAIQQA
FOXG_15625T0  104 --------KRAKTADEKEQRRVERVLRNRRAAQSSRERKRQEVEQLEKRNKDLEAAIQQA

FOXG_14230T0  160 QEANARLMMELEKMRRSSGVA-AHSSPLESFRENP-SLSQELFSSQTTHKAMEELISPNA
Necha2_99800  157 QRTNMLLANELAQYRRTYGIVTRSSSPLDSLRENAVTLSPELFGSQNATGMVDVMTPPHA
FGSG_11623T0  177 QEANAKLMSELEQIRRSGAVS-YSPSVLDSFRVNP-SLSQELFGSKHTHEPIDGLVSSNT
FOWG_17414T0  160 QEANARLMMELEKMRRSSGVA-AHSSPLESFRENP-SLSQELFSSQTTHKAMEELISPNA
FOXB_02757T0  160 QEANARLMMELEKMRRSSGVA-AHSSPLESFRENP-SLSQELFSSQTTHKAMEELISPNA
FVEG_13087T0  157 EQMNARLMDELAQMRKANGL--------------P-PLSQELFTPQTTHQPAETMVSPNA
FOCG_08851T0  156 EQMNARLMDELAQMRKANGL--------------P-PLSQELFSPQTTHQPTETTVSPNA
FOWG_12843T0  156 EQMNARLMDELAQMRKANGL--------------P-PLSQELFSPQTTHQPTETTVSPNA
FOZG_08258T0  156 EQMNARLMDELAQMRKANGL--------------P-PLSQELFSPQTTHQPTETTVSPNA
FOYG_10272T0  156 EQMNARLMDELAQMRKANGL--------------P-PLSQELFTPQTTHQPTETTVSPNA
FOMG_15447T0  156 EQMNARLMDELAQMRKANGL--------------P-PLSQELFSPQTTHQPTETTVSPNA
FOTG_11072T0  156 EQMNARLMDELAQMRKANGL--------------P-PLSQELFSPQTTHQPTETTVSPNA
FOPG_10256T0  156 EQMNARLMDELAQMRKANGL--------------P-PLSQELFSPQTTHQPTETTVSPNA
FOQG_14359T0  156 EQMNARLMDELAQMRKANGL--------------P-PLSQELFSPQTTHQPTETTVSPNA
FOXB_03192T0  156 EQMNARLMDELAQMRKANGL--------------P-PLSQELFSPQTTHQPTETTVSPNA
FOVG_08874T0  156 EQMNARLMDELAQMRKANGL--------------P-PLSQELFSPQTTHQPTETTVSPNA
FOIG_12632T0  156 EQMNARLMDELAQMRKANGL--------------P-PLSQELFSPQTTHQPTETTVSPNA
FOXG_15625T0  156 EQMNARLMDELAQMRKANGL--------------P-PLSQELFSPQTTHQPTETTVSPNA
```

SUPP FIG 3

TF6: Cys2His2 domain (Pfam: zf-C2H2)

```
                                                                    C    C   F
FOWG_17023T0   149 KVIQRKADLQVGAGQQPLAPVMSSVS---VISQGTPGSIDNKTARKRYRCDGPSCQKSFT
FOXG_14211T0   149 KVIQRKADLQVGAGQQPLAPVMSSVS---VISQGTPGSIDNKTARKRYRCDGPSCQKSFT
FOXB_13262T0   170 KTIQRKADMQVNTVQQPLTPGMSPVSEASFKSQGTPGPMERKAPRKRYRCDGPNCQKSFT
FOPG_16495T0   170 KTIQRKADMQVNTVQQPLTPGMSPVSEASFKSQGTPGPMERKAPRKRYRCDGPNCQKSFT
FOWG_16941T0   179 KTIQRKADMQVHTIQQPLTPGMSPVPEASFESQGIPGPMNNKTGRKRYSCNGPDCQKSFD
FOVG_18094T0   180 KTIQRKADMQVNTVQQPLTPGMSPVSEASFESQGTPGPMDNKASRKRYRCDGPNCQKSFT
FOTG_14885T0   180 KTIQRKADMQVNTVQQPLTPGMSRVSEASFESQGTPGPMDNKASRKRYRCDGPGCQKSFT
FVEG_07926T0   180 KAIQRKADLQVDTVQQPLTPGMSPVSEASFESQGTPGPMDNKTARKRYRCDGPNCQKSFT
Necha2_48671   181 KAIQRKGEKELDVAQQPLTPGMSPVSEASLENNGTPAPADSKTSKKRYVCNGPNCRKSFT
FGSG_13123T0     2 KAIQRKAEMQPDAGHQPLTPGMSPVSDASLESHGTPTPMGDKSQRKRYHCNGPNCQKSFT
FOXG_01564T0   180 KAIQRKVDLQVDTVQQPLTPGMSPVSEASFESQGTPGPMDSKTARKRYRCDGPNCQKSFT
FOMG_05581T0   180 KAIQRKVDLQVDTVQQPLTPGMSPVSEASFESQGTPGPMDSKTARKRYRCDGPNCQKSFT
FOZG_05741T0   180 KAIQRKVDLQVDTVQQPLTPGMSPVSEASFESQGTPGPMDSKTARKRYRCDGPNCQKSFT
FOCG_01246T0   180 KAIQRKVDLQVDTVQQPLTPGMSPVSEASFESQGTPGPMDSKTARKRYRCDGPNCQKSFT
FOYG_03407T0   180 KAIQRKVDLQVDTVQQPLTPGMSPVSEASFESQGTPGPMDSKTARKRYRCDGPNCQKSFT
FOWG_02138T0   180 KAIQRKVDLQVDTVQQPLTPGMSPVSEASFESQGTPGPMDSKTARKRYRCDGPNCQKSFT
FOIG_02817T0   180 KAIQRKADLQVDTVQQPLTPGMSPVSEASFESQGTPGPMDSKTARKRYRCDGPNCQKSFT
FOTG_00289T0   178 KAIQRKADLQVDTVQQPLTPGMSPVSEASFESQGTPGPMDSKTARKRYRCDGPNCQKSFT
FOXB_10721T0   178 KAIQRKADLQVDTVQQPLTPGMSPVSEASFESQGTPGPMDSKTARKRYRCDGPNCQKSFT
FOPG_04820T0   178 KAIQRKADLQVDTVQQPLTPGMSPVSEASFESQGTPGPMDSKTARKRYRCDGPNCQKSFT
FOVG_03275T0   178 KAIQRKADLQVDTVQQPLTPGMSPVSEASFESQGTPGPMDSKTARKRYRCDGPNCQKSFT
FOQG_00302T0   178 KAIQRKADLQVDTVQQPLTPGMSPVSEASFESQGTPGPMDSKTARKRYRCDGPNCQKSFT

                       L  H   H       C    C   F     L  H   H       C  C   F
FOWG_17023T0   206 GKTHLDIHRRTHTGIKPYICDFPGCDLTFSQLGNLKTHKRRHTGERPFACEQCDRHFSQR
FOXG_14211T0   206 GKTHLDIHRRTHTGIKPYICDFPGCDLTFSQLGNLKTHKRRHTGERPFACEQCDRHFSQR
FOXB_13262T0   230 QKLHLDIHRRTHTGIKPYNCDFPGCDLSFSQLGNLKTHKRRHTGERPFACDKCDRHFAQR
FOPG_16495T0   230 QKLHLDIHRRTHTGIKPYNCDFPGCDLSFSQLGNLKTHKRRHTGERPFACDKCDRHFAQR
FOWG_16941T0   239 QQAHLDIHRRTHTGIKPYNCDFPGCDLTFSQLGNLKTHRRRHTGERPFACDKCDRHFAQS
FOVG_18094T0   240 QKVHLDIHRRTHTGIKPYNCDFPGCDLTFSQLGNLKTHRRRHTGERPFACDKCDRHFAQR
FOTG_14885T0   240 QKAHLNIHRRTHTGVKPYNCDFLGCDLSFSQLGNLKTHKRRHTGERPFACDKCGRNFAQR
FVEG_07926T0   240 QKTHLDIHRRTHTGIKPYNCDFPGCDLTFSQLGNLKTHRRRHTGERPFACDKCDRHFAQR
Necha2_48671   241 QKTHLDIHRRTHTGDKPYTCEFPGCKLTFSQLGNLKTHMRRHTGERPYSCGKCGRKFAQR
FGSG_13123T0    62 QKTHLDIHRRTHSGEKPYSCDFPGCELTFSQLGNLKTHRRRHTGERPFSCDKCDRQFAQR
FOXG_01564T0   240 QKTHLDIHRRTHTGIKPYNCDFPGCDLTFSQLGNLKTHRRRHTGERPFACDKCDRHFAQR
FOMG_05581T0   240 QKTHLDIHRRTHTGIKPYNCDFPGCDLTFSQLGNLKTHRRRHTGERPFACDKCDRHFAQR
FOZG_05741T0   240 QKTHLDIHRRTHTGIKPYNCDFPGCDLTFSQLGNLKTHRRRHTGERPFACDKCDRHFAQR
FOCG_01246T0   240 QKTHLDIHRRTHTGIKPYNCDFPGCDLTFSQLGNLKTHRRRHTGERPFACDKCDRHFAQR
FOYG_03407T0   240 QKTHLDIHRRTHTGIKPYNCDFPGCDLTFSQLGNLKTHRRRHTGERPFACDKCDRHFAQR
FOWG_02138T0   240 QKTHLDIHRRTHTGIKPYNCDFPGCDLTFSQLGNLKTHRRRHTGERPFACDKCDRHFAQR
FOIG_02817T0   240 QKTHLDIHRRTHTGIKPYNCDFPGCDLTFSQLGNLKTHRRRHTGERPFACDKCDRHFAQR
FOTG_00289T0   238 QKTHLDIHRRTHTGIKPYNCDFPGCDLTFSQLGNLKTHRRRHTGERPFACDKCDRHFAQR
FOXB_10721T0   238 QKTHLDIHRRTHTGIKPYNCDFPGCDLTFSQLGNLKTHRRRHTGERPFACDKCDRHFAQR
FOPG_04820T0   238 QKTHLDIHRRTHTGIKPYNCDFPGCDLTFSQLGNLKTHRRRHTGERPFACDKCDRHFAQR
FOVG_03275T0   238 QKTHLDIHRRTHTGIKPYNCDFPGCDLTFSQLGNLKTHRRRHTGERPFACDKCDRHFAQR
FOQG_00302T0   238 QKTHLDIHRRTHTGIKPYNCDFPGCDLTFSQLGNLKTHRRRHTGERPFACDKCDRHFAQR

                     L  H   H
FOWG_17023T0   266 GNLQAHLESHKGLKPFICILDDCNKTFTLLGNMKTHQNSLHKETLEELTMKFTKIIASGE
FOXG_14211T0   266 GNLQAHLESHKGLKPFICILDDCNKTFTLLGNMKTHQNSLHKETLEELTMKFTKIIASGE
FOXB_13262T0   290 GNLRAHLETHKGLKPFICILDDCNKTFTKLGNMKAHQNHFHKKTLNKLTMKFAQIIALGE
FOPG_16495T0   290 GNLRAHLETHKGLKPFICILDDCNKTFTKLGNMKAHQNHFHKKTLNKLTMKFAQIIALGE
FOWG_16941T0   299 GNLRAHLLTHQGLKPFSCILDDCNKTFTILGNMKAHQNKFHKKTLKKLTTKFAKIIASGK
FOVG_18094T0   300 GNLRAHLQTHQGLKPC--------------------------------------------
FOTG_14885T0   300 GNLQAHLKSHKGLKPFICILDDCNKTFTQLGNMKAHQNKFHKETLKKLTTKFAKIIASGK
FVEG_07926T0   300 GNLRAHLQTHQGLKPFVCILDDCNKTFSQLGNMKTHQNNFHKKTLKKLTMKFAKIIASGE
Necha2_48671   301 GNVRAHEQTHQGLKPFICRLDDCNKTFSQLGNMKTHQNNFHKKTLKNLTMRFAQILNSGE
FGSG_13123T0   122 GNLRAHLQTHQGLKPYVCILDACNKTFSQLGNMKTHQNNFHKKTLKNLTMRFAQILNSGE
FOXG_01564T0   300 GNLRAHLQTHQGLKPFICILDDCNKTFSQLGNMKTHQNNFHKKTLKKLTMKFANIIASGE
FOMG_05581T0   300 GNLRAHLQTHQGLKPFICILDDCNKTFSQLGNMKTHQNNFHKKTLKKLTMKFANIIASGE
FOZG_05741T0   300 GNLRAHLQTHQGLKPFICILDDCNKTFSQLGNMKTHQNNFHKKTLKKLTMKFANIIASGE
FOCG_01246T0   300 GNLRAHLQTHQGLKPFICILDDCNKTFSQLGNMKTHQNNFHKKTLKKLTMKFANIIASGE
FOYG_03407T0   300 GNLRAHLQTHQGLKPFICILDDCNKTFSQLGNMKTHQNNFHKKTLKKLTMKFANIIASGE
FOWG_02138T0   300 GNLRAHLQTHQGLKPFICILDDCNKTFSQLGNMKTHQNNFHKKTLKKLTMKFANIIASGE
FOIG_02817T0   300 GNLRAHLQTHQGLKPFICILDDCNKTFSQLGNMKTHQNNFHKKTLKKLTMKFANIIASGE
FOTG_00289T0   298 GNLRAHLQTHQGLKPFICILDDCNKTFSQLGNMKTHQNNFHKKTLKKLTMKFANIIASGE
FOXB_10721T0   298 GNLRAHLQTHQGLKPFICILDDCNKTFSQLGNMKTHQNNFHKKTLKKLTMKFANIIASGE
FOPG_04820T0   298 GNLRAHLQTHQGLKPFICILDDCNKTFSQLGNMKTHQNNFHKKTLKKLTMKFANIIASGE
FOVG_03275T0   298 GNLRAHLQTHQGLKPFICILDDCNKTFSQLGNMKTHQNNFHKKTLKKLTMKFANIIASGE
FOQG_00302T0   298 GNLRAHLQTHQGLKPFICILDDCNKTFSQLGNMKTHQNNFHKKTLKKLTMKFANIIASGE
```

SUPP FIG 3

TF7: Cys2His2 domain (Pfam: zf-C2H2)

```
                                 C    C    F     L  H   H
FOQG_18533T0  266 GI------A--GSYTCTVHGCNAAPFQTQYLLNSHMNVHSSIRPHYCPVKGCPRSEGGKG
FOXG_19464T0  266 GI------A--GSYTCTVHGCNAAPFQTQYLLNSHMNVHSSIRPHYCPVKGCPRSEGGKG
FOXG_22293T0  266 GI------A--GSYTCTVHGCNAAPFQTQYLLNSHMNVHSSIRPHYCPVKGCPRSEGGKG
FOXG_22413T0  266 GI------A--GSYTCTVHGCNAAPFQTQYLLNSHMNVHSSIRPHYCPVKGCPRSEGGKG
FOXB_08620T0  266 GI------A--GSYTCTVHGCNAAPFQTQYLLNSHMNVHSSIRPHYCPVKGCPRSEGGKG
FOPG_18275T0  266 GI------A--GSYTCTVHGCNAAPFQTQYLLNSHMNVHSSIRPHYCPVKGCPRSEGGKG
FOXG_14275T0  287 GMTHPQPIT--GLYTCTVQGCHVEPFQTQYLLNSHMNVHSSVRPHYCPVKGCPRSEGGKG
FOWG_17588T1  287 GMTHPQPIA--GLYTCTFQGCRVDPFQTKYLLNSHMNVHSSVRLHYCPVKGCPRSEGGKG
FOXG_21311T0  287 GMTHPQPIA--GLYTCTFQGCRVEPFQTKYLLNSHMNVHSSVRLHYCPVKGYPRSEGGKG
FOXG_22662T0  287 GMTHPQPIA--GLYTCTFQGCRVEPFQTKYLLNSHMNVHSSVRLHYCPVKGYPRSEGGKG
FOXG_21046T0  287 GMTHPQPIA--GLYTCTFQGCRVEPFQTKYLLNSHMNVHSSVRLHYCPVKGYPRSEGGKG
FOXG_21006T0  287 GMTHPQPIA--GLYTCTFQGCRVEPFQTKYLLNSHMNVHSSVRLHYCPVKGYPRSEGGKG
FOMG_18197T0  287 GMTHPQPIT--GLYTCTVQGCHVEPFQTQYILNSHMNVHSSVRPHYCPVKGCPRSEGGKG
FOVG_18828T0  287 GMTHPQPIT--GLYTCTVQGCHVEPFQTKYLLNSHMNVHSSVRPHYCPVKGCPRSEGGKG
FOQG_16628T0  287 GMTHPPALI-----ICQLNQS-ITPIKSIKSSNSSF---QSNQITHC-------------
FOQG_19377T0  346 GITHPQPVP--GSYTCTANGCNAAPFRTQYLLNSHMDVHSSVRPHYCPVKECPRSGGGKG
FOTG_18879T0  346 GITHPQPVL--GSYTCTANGCNAAPFRTQYLLNSHMNVHSSVRPHYCPVKECPRSEGGKG
FOXB_03115T0  346 GITHPQPVL--GSYTCTANGCNAAPFRTQYLLNSHMNVHSSVRPHYCPVKECPRSEGGKG
FOQG_19073T0  346 GITHPQPVP--GSYTCTANGCNAAPFRTQYLLNSHMNVHSSVRPHYCPVKECPRSEGGKG
Necha2_47170  340 GITNPQPATGTGAYVCNFAGCTALPFQTQYLLNSHANVHSSARPHYCPVKGCPRSEGGKG
FGSG_05381T0  228 GITHPQHMT--GSYTCTVPGCNAAPFQTQYLLNSHMNVHSSARPHYCPVKGCPRSEGGKG
FVEG_07153T0  357 GITHPQPLS--GSYTCTVTGCNAAPFQTQYLLNSHMNVHSSIRPHYCPVKGCPRSEGGKG
FOXG_20064T0  262 GITHPQPLS--GSYTCTVTGCNAAPFQTQYLLNSHMNVHSSIRPHYCPVKGCPRSEGGKG
FOIG_07901T1  357 GITHPQPLS--GSYTCTVAGCNAAPFQTQYLLNSHMNVHSSIRPHYCPVKGCPRSEGGKG
FOQG_03601T0  357 GITHPQPLS--GSYTCTVTGCNAAPFQTQYLLNSHMNVHSSIRPHYCPVKGCPRSEGGKG
FOWG_04951T1  357 GITHPQPLS--GSYTCTVTGCNAAPFQTQYLLNSHMNVHSSIRPHYCPVKGCPRSEGGKG
FOZG_12836T0  357 GITHPQPLS--GSYTCTVTGCNAAPFQTQYLLNSHMNVHSSIRPHYCPVKGCPRSEGGKG
FOTG_03788T1  357 GITHPQPLS--GSYTCTVTGCNAAPFQTQYLLNSHMNVHSSIRPHYCPVKGCPRSEGGKG
FOVG_10762T1  357 GITHPQPLS--GSYTCTVTGCNAAPFQTQYLLNSHMNVHSSIRPHYCPVKGCPRSEGGKG
FOPG_01942T1  357 GITHPQPLS--GSYTCTVTGCNAAPFQTQYLLNSHMNVHSSIRPHYCPVKGCPRSEGGKG
FOXB_19015T1  357 GITHPQPLS--GSYTCTVTGCNAAPFQTQYLLNSHMNVHSSIRPHYCPVKGCPRSEGGKG
FOCG_11063T0  357 GITHPQPLS--GSYTCTVTGCNAAPFQTQYLLNSHMNVHSSIRPHYCPVKGCPRSEGGKG
FOYG_11053T0  357 GITHPQPLS--GSYTCTVTGCNAAPFQTQYLLNSHMNVHSSIRPHYCPVKGCPRSEGGKG
FOMG_08371T0  357 GITHPQPLS--GSYTCTVTGCNAAPFQTQYLLNSHMNVHSSIRPHYCPVKGCPRSEGGKG
```

SUPP FIG 3
