# Supplementary material for: Transcription Factors Encoded on Core and Accessory Chromosomes of Fusarium oxysporum Induce Expression of Effector Genes
Source: PLoS Genet. 2016 Nov 17;12(11):e1006401. doi: 10.1371/journal.pgen.1006401 (PMC5140021; doi:10.1371/journal.pgen.1006401)
Supplement: S4 Fig — A) The number of genes with one or more transcription factor DNA binding site (TF DBS) in the 1000 bp upstream of the predicted transcriptional start site, for the complete genome, and for a subset of genes (up- or down-regulated during infection). Boxes indicate a significant enrichment (p value <0.01 after Bonferroni correction). The motif found in effector gene promoters earlier (aacTGCCGa and overlapping with the Tf1 DNA binding site has been included. For all categories the total number of genes is given (ALL, green), the number of core genes (CORE, blue) and the number of accessory genes (purple). Accessory genes are subdivided in total (ACC), accessory genes not on the pathogenicity chromosome (no14) and the accessory genes on the pathogenicity chromosome (only_14). B) As A, but for the 1000 bp downstream the ATG. C) As A, but only for the Tf1 DNA binding site (≥3 occurrences), the motif found in the promoters of effector genes, overlapping with the Tf1 DNA binding site (≥1 occurrences) and the Sge1 DNA binding site (≥1 occurrences). The following categories are additional: genes UP or DOWN regulated in the aTF1, cTF1 or SGE1 overexpressors, genes for small secreted proteins, genes with a MIMP in their promoter, genes coding for proteins found in xylem sap of infected plants. D) As C, but for the 1000 bp downstream the ATG. (PDF) [file pgen.1006401.s004.pdf]

A

≥1 TF DBS IN PROMOTER	all genes ALL	CORE	ACC	no 14	only14	gene upregulated in planta ALL	CORE	ACC	no 14	only14	genes downregulated in planta ALL	CORE	ACC	no 14	only14
aTF1 fwd	15351	11747	**3604**	**3271**	332	**726**	**572**	154	104	50	105	97	8	8	0
aTF1 rev	15399	11884	3515	3190	323	700	554	146	96	50	115	105	10	10	0
effector motif fwd	136	119	17	14	2	**20**	**18**	2	1	1	0	0	0	0	0
effector motif rev	141	119	22	19	3	14	11	3	0	3	0	0	0	0	0
aTF2 fwd	13664	10654	3010	2725	284	602	484	118	79	39	88	79	9	9	0
aTF2 rev	14250	**11366**	2884	2589	293	604	480	124	77	47	94	88	6	6	0
cTF4 fwd	3650	2681	**969**	**884**	85	155	119	36	32	4	26	23	3	3	0
cTF4 rev	3610	2666	**944**	**865**	79	156	116	40	30	10	26	24	2	2	0
aTF5 fwd	9017	6840	**2177**	1967	210	403	313	90	65	25	62	53	9	9	0
aTF5 rev	9292	7110	2182	1973	209	393	300	93	64	29	68	59	9	9	0
aTF7 fwd	10982	8497	2485	2277	208	453	363	90	65	25	75	70	5	5	0
aTF7 rev	10460	8063	2397	2180	216	426	346	80	57	23	73	67	6	6	0
SGE1 fwd	2749	2034	**715**	641	74	**178**	**141**	37	16	**21**	19	16	3	3	0
SGE1 rev	2770	2022	**748**	**679**	69	**179**	**142**	37	18	19	16	15	1	1	0
ALL	20932	16174	4758	4321	436	896	708	188	125	63	140	127	13	13	0

B

≥1 TF DBS IN DOWNSTREAM ATG	all genes ALL	CORE	ACC	no 14	only14	gene upregulated in planta ALL	CORE	ACC	no 14	only14	genes downregulated in planta ALL	CORE	ACC	no 14	only14
aTF1 fwd	17693	13556	**4133**	**3766**	365	758	600	158	113	45	115	104	11	11	0
aTF1 rev	17096	13100	**3993**	**3630**	361	741	594	147	103	44	115	105	10	10	0
effector motif fwd	85	62	23	21	2	3	2	1	1	0	0	0	0	0	0
effector motif rev	108	87	21	20	1	6	5	1	1	0	1	1	0	0	0
aTF2 fwd	13768	10747	3018	2742	274	565	455	110	73	37	95	87	8	8	0
aTF2 rev	13692	10639	3051	2766	283	567	439	128	89	39	88	82	6	6	0
cTF4 fwd	3616	2710	906	**836**	70	166	129	37	27	10	27	23	4	4	0
cTF4 rev	4226	3186	1040	952	87	179	154	25	16	9	32	28	4	4	0
aTF5 fwd	9385	7134	**2250**	2042	207	377	291	86	59	27	62	54	8	8	0
aTF5 rev	9609	7360	2248	2049	198	399	314	85	58	27	67	61	6	6	0
aTF7 fwd	11106	8619	2487	2249	237	457	367	90	63	27	69	63	6	6	0
aTF7 rev	9961	7696	2263	2051	212	417	334	83	53	30	63	58	5	5	0
SGE1 fwd	2168	1649	516	472	44	107	81	26	20	6	12	11	1	1	0
SGE1 rev	2255	1714	540	486	54	94	75	19	12	7	20	19	1	1	0
ALL	20932	16174	4758	4321	436	896	708	188	125	63	140	127	13	13	0

C

	TF DBS IN PROMOTER	ALL ALL	CORE	ACC	no 14	only 14	in planta UP ALL	CORE	ACC	no 14	only 14	in planta DOWN ALL	CORE	ACC	no 14	only 14
≥3	aTF1 fwd	3528	2621	**907**	**826**	80	188	154	34	19	15	27	26	1	1	0
≥3	aTF1 rev	3491	2631	860	772	87	**214**	**164**	50	29	21	30	25	5	5	0
≥1	effector motif fwd	136	119	17	14	2	**20**	**18**	2	1	1	0	0	0	0	0
≥1	effector motif rev	141	119	22	19	3	14	11	3	0	3	0	0	0	0	0
≥1	SGE1 fwd	2749	2034	**715**	641	74	**178**	**141**	37	16	**21**	19	16	3	3	0
≥1	SGE1 rev	2770	2022	**748**	**679**	69	**179**	**142**	37	18	19	16	15	1	1	0
	ALL	20932	16174	4758	4321	436	896	708	188	125	63	140	127	13	13	0

	TF DBS IN PROMOTER	SSPs ALL	CORE	ACC	no 14	only 14	with MIMP ALL	CORE	ACC	no 14	only 14	found in XS ALL	CORE	ACC	no 14	only 14
≥3	aTF1 fwd	160	113	47	41	6	22	2	20	9	11	14	8	6	0	6
≥3	aTF1 rev	145	112	33	27	6	24	1	23	8	15	18	9	9	1	8
≥1	effector motif fwd	9	8	1	0	1	1	0	1	0	1	2	1	1	0	1
≥1	effector motif rev	**20**	**17**	3	1	2	1	0	1	0	1	2	1	1	0	1
≥1	SGE1 fwd	**155**	118	37	31	6	**22**	0	**22**	6	**16**	**22**	13	**9**	1	**8**
≥1	SGE1 rev	**166**	117	**49**	41	8	**24**	1	**23**	7	**16**	13	8	5	0	5
	ALL	876	671	204	181	23	73	4	69	28	41	55	36	19	3	16

	TF DBS IN PROMOTER	aTF1 UP ALL	CORE	ACC	no 14	only 14	cTF1 UP ALL	CORE	ACC	no 14	only 14	SGE1 UP ALL	CORE	ACC	no 14	only 14
≥3	aTF1 fwd	31	20	11	0	11	**26**	**14**	12	2	10	30	19	11	3	8
≥3	aTF1 rev	**35**	16	19	3	**16**	**27**	**10**	**17**	1	**16**	31	17	14	2	12
≥1	effector motif fwd	**6**	**5**	1	0	1	**5**	**4**	1	0	1	5	4	1	0	1
≥1	effector motif rev	**6**	**4**	2	0	2	**5**	**3**	2	0	2	2	0	2	0	2
≥1	SGE1 fwd	**34**	**16**	**18**	2	**16**	**20**	8	12	1	11	**34**	20	**14**	1	**13**
≥1	SGE1 rev	**38**	**18**	**20**	4	**16**	**21**	5	**16**	5	11	**39**	23	**16**	**4**	**12**
	ALL	103	53	50	14	36	65	25	40	10	30	117	82	34	5	29

	TF DBS IN PROMOTER	aTF1 DOWN ALL	CORE	ACC	no 14	only 14	cTF1 DOWN ALL	CORE	ACC	no 14	only 14	SGE1 DOWN ALL	CORE	ACC	no 14	only 14
≥3	aTF1 fwd	64	9	55	52	3	77	12	65	63	2	68	7	61	58	3
≥3	aTF1 rev	43	10	33	29	4	59	13	46	44	2	43	8	35	33	2
≥1	effector motif fwd	1	0	1	0	1	1	0	1	1	0	1	0	1	0	1
≥1	effector motif rev	1	0	1	1	0	5	1	4	4	0	0	0	0	0	0
≥1	SGE1 fwd	25	4	21	21	0	42	7	35	34	1	29	5	24	23	1
≥1	SGE1 rev	27	2	25	25	0	37	3	34	33	1	32	4	28	27	1
	ALL	273	51	222	212	10	347	57	290	283	7	278	43	235	226	9

D

	TF DBS 1000 bp DOWNSTREAM ATG	ALL ALL	CORE	ACC	no 14	only 14	in planta UP ALL	CORE	ACC	no 14	only 14	in planta DOWN ALL	CORE	ACC	no 14	only 14
≥3	aTF1 fwd	6664	4999	**1664**	**1514**	150	297	236	61	47	14	35	34	1	1	0
≥3	aTF1 rev	5678	4191	**1486**	**1340**	144	269	209	60	45	15	42	41	1	1	0
≥1	effector motif fwd	85	62	23	21	2	3	2	1	1	0	0	0	0	0	0
≥1	effector motif rev	108	87	21	20	1	6	5	1	1	0	1	1	0	0	0
≥1	SGE1 fwd	2168	1649	516	472	44	107	81	26	20	6	12	11	1	1	0
≥1	SGE1 rev	2255	1714	540	486	54	94	75	19	12	7	20	19	1	1	0
	ALL	20932	16174	4758	4321	436	896	708	188	125	63	140	127	13	13	0

	TF DBS 1000 bp DOWNSTREAM ATG	SSPs ALL	CORE	ACC	no 14	only 14	with MIMP ALL	CORE	ACC	no 14	only 14	found in XS ALL	CORE	ACC	no 14	only 14
≥3	aTF1 fwd	274	194	80	72	8	22	2	20	14	6	25	19	6	2	4
≥3	aTF1 rev	263	194	69	62	7	22	0	22	13	9	18	17	1	0	1
≥1	effector motif fwd	8	4	4	3	1	0	0	0	0	0	1	1	0	0	0
≥1	effector motif rev	4	4	0	0	0	0	0	0	0	0	0	0	0	0	0
≥1	SGE1 fwd	117	85	32	29	3	12	2	10	7	3	7	6	1	0	1
≥1	SGE1 rev	124	92	32	28	4	11	0	11	2	9	4	3	1	0	1
	ALL	876	671	204	181	23	73	4	69	28	41	55	36	19	3	16

	TF DBS 1000 bp DOWNSTREAM ATG	aTF1 UP ALL	CORE	ACC	no 14	only 14	cTF1 UP ALL	CORE	ACC	no 14	only 14	SGE1 UP ALL	CORE	ACC	no 14	only 14
≥3	aTF1 fwd	29	17	12	6	6	19	9	10	4	6	36	28	8	2	6
≥3	aTF1 rev	27	17	10	3	7	16	8	8	3	5	26	21	5	1	4
≥1	effector motif fwd	1	0	1	1	0	1	0	1	1	0	0	0	0	0	0
≥1	effector motif rev	1	1	0	0	0	0	0	0	0	0	1	1	0	0	0
≥1	SGE1 fwd	17	9	8	4	4	8	3	5	2	3	17	13	4	1	3
≥1	SGE1 rev	19	10	9	4	5	11	5	6	2	4	20	16	4	0	4
	ALL	103	53	50	14	36	65	25	40	10	30	117	82	34	5	29

	TF DBS 1000 bp DOWNSTREAM ATG	aTF1 DOWN ALL	CORE	ACC	no 14	only 14	cTF1 DOWN ALL	CORE	ACC	no 14	only 14	SGE1 DOWN ALL	CORE	ACC	no 14	only 14
≥3	aTF1 fwd	82	13	69	67	2	110	18	92	90	2	92	12	80	78	2
≥3	aTF1 rev	81	11	70	66	4	110	18	92	90	2	83	9	74	71	3
≥1	effector motif fwd	1	0	1	1	0	1	0	1	1	0	1	0	1	1	0
≥1	effector motif rev	0	0	0	0	0	0	0	0	0	0	0	0	0	0	0
≥1	SGE1 fwd	29	3	26	25	1	38	5	33	32	1	27	3	24	22	2
≥1	SGE1 rev	26	6	20	20	0	34	8	26	26	0	27	5	22	22	0
	ALL	273	51	222	212	10	347	57	290	283	7	278	43	235	226	9

SUPP FIG 4
