# Supplementary material for: Transcription Factors Encoded on Core and Accessory Chromosomes of Fusarium oxysporum Induce Expression of Effector Genes
Source: PLoS Genet. 2016 Nov 17;12(11):e1006401. doi: 10.1371/journal.pgen.1006401 (PMC5140021; doi:10.1371/journal.pgen.1006401)
Supplement: S5 Fig — A) Estimated percentage, by visual inspection, of GFP-fluorescent spores in independent transformants of different transcription factor overexpression constructs. x: this transformant does not exist (misnumbered), *: of the background strain (Psix1GFP—WT) the same transformant was tested multiple times, each number represents therefore a replicate instead of a different transformant. B) Relative expression of different transcription factor genes in WT and their respective overexpressors as determined by Q-RT-PCR. Relative expression is calculated as TF expression/expression of EF1α. Minimally three independent transformants were tested per transcription factor. Error bars indicate the standard deviation of two technical replicates. C) Growth rate of the different transcription factor overexpressors. Minimally three independent transformants were tested per transcription factor, on both rich medium (potato dextrose agar, PDA, top panel) and complete minimal medium (Czapek Dox Agar, CDA, bottom panel). Stars indicate significant differences compared to the background strain (T-test, p<0.01). Error bars indicate the standard deviation of minimally two independent replicates. D) Bioassay on susceptible tomato plants. Minimally three independent transformants were tested per transcription factor. Control treatments were: mock, WT (this is the WT used to make the background strain, Fol007), Psix1GFP (the background strain, WT + reporter construct Psix1GFP), Psix1GFP + RFP (the background strain plus a constitutively expressed RFP construct). Top panel: average plant weight (gram) 21 dpi. Error bars indicate standard deviation. Stars indicate a significant difference from the background strain (Psix1GFP, T-test, p<0.01). Bottom panel: disease index (0–4 arbitrary units) 21 dpi. (PDF) [file pgen.1006401.s005.pdf]

Estimated percentage of GFP fluorescent spores in independant transformants of different transcription factor overexpression constructs

TF-construct	A	B	C	D	E	F	G	H	I	J	K	L
aTF1-short	5	5	10	12	2	10	10	15	1	1	1	1
aTF1-long	100	0	1	7	100	100	0	0	1	80	40	1
aTF2	1	1	1	1	1	1	1	1	1	1	1	1
aTF3	3	3	2	2	0	1	3	2	1	1	1	1
aTF4	1	1	1	1	1	0	1	1	0	0	0	
aTF5	1	1	1	1	0	2	2	1	1	1	3	1
aTF6	0	0	2	2	5	1	0	1	2	1	1	
aTF7	8	6	10	5	1	10	15	1	3	10	10	5
cTF1	60	25	70	100	100	5	100	2	30	70	100	30
cTF7	15	1	1	1	1	0	1	3	1	1	1	2
SGE1	3	3	3	3	3	5	3	3	3	3	1	20
Psix1GFP*	1	8	1	1	1	1	1					

(transformant number, continued)

TF-construct	M	N	O	P	Q	R	S	T	U	V	W
aTF1-short	20	x	5	7	20	3					
aTF1-long	1	x	1	100	0						
aTF2	1	1	1								
aTF3	2	0	0	1	1	1	1	2	1		
aTF4											
aTF5	3	1	1	1	1						
aTF6											
aTF7	3	15	1	15	15	10	10	7	3	1	0
cTF1	1	25	5	1	0	1	20				
cTF7	1	1	2	1	10	2					
SGE1	70	1									
Psix1GFP*											

x: misnumbered, transformant #N does not exist for this TF overexpression construct
*: of the background strain the same transformant was tested multiple times,
each number represents therefore a replicate instead of a different transformant.

SUPP FIG 5A

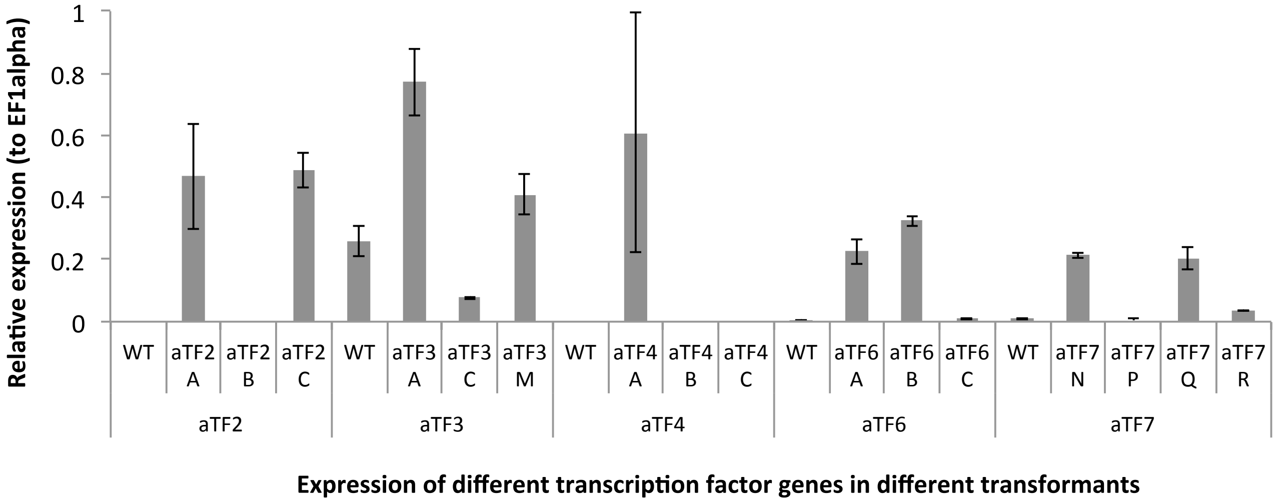

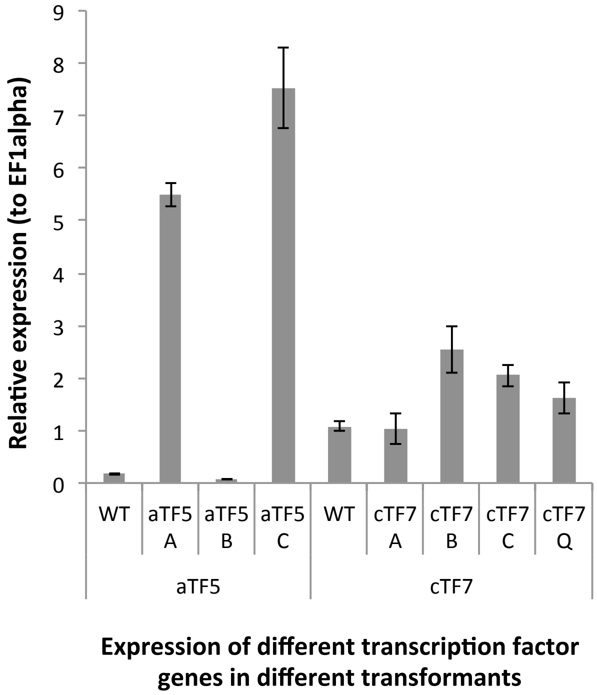

SUPP FIGURE 5B

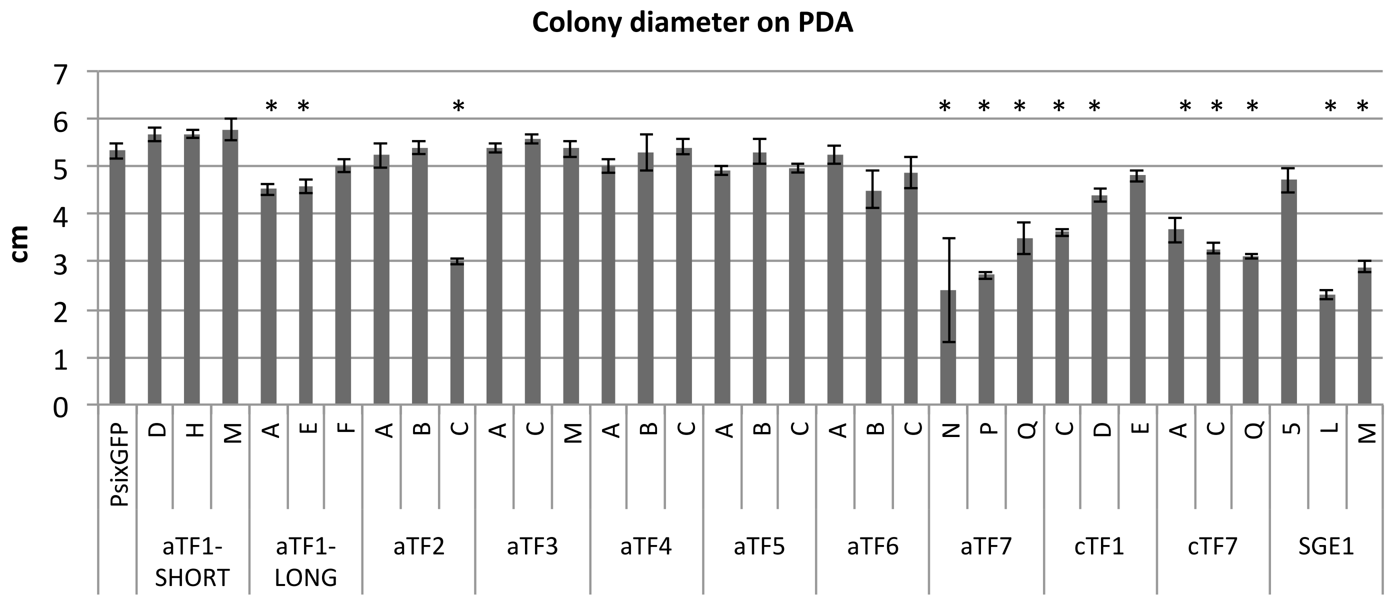

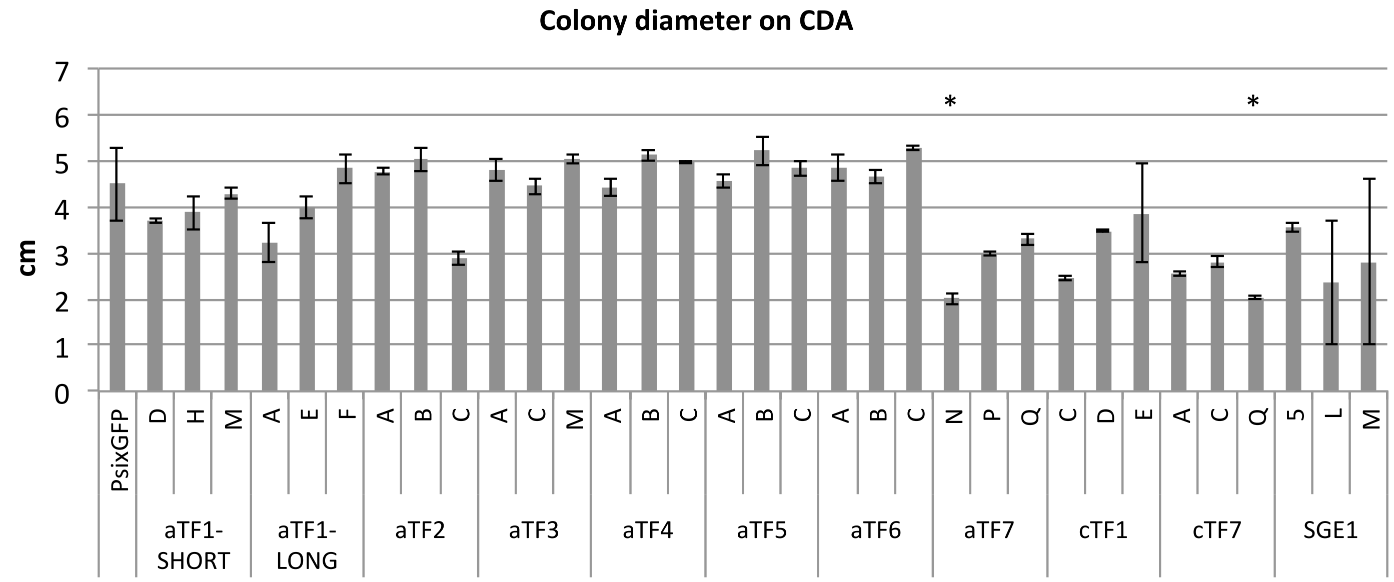

* Significantly different (p<0.01, T-test) to background strain (PsixGFP)

SUPP FIGURE 5C

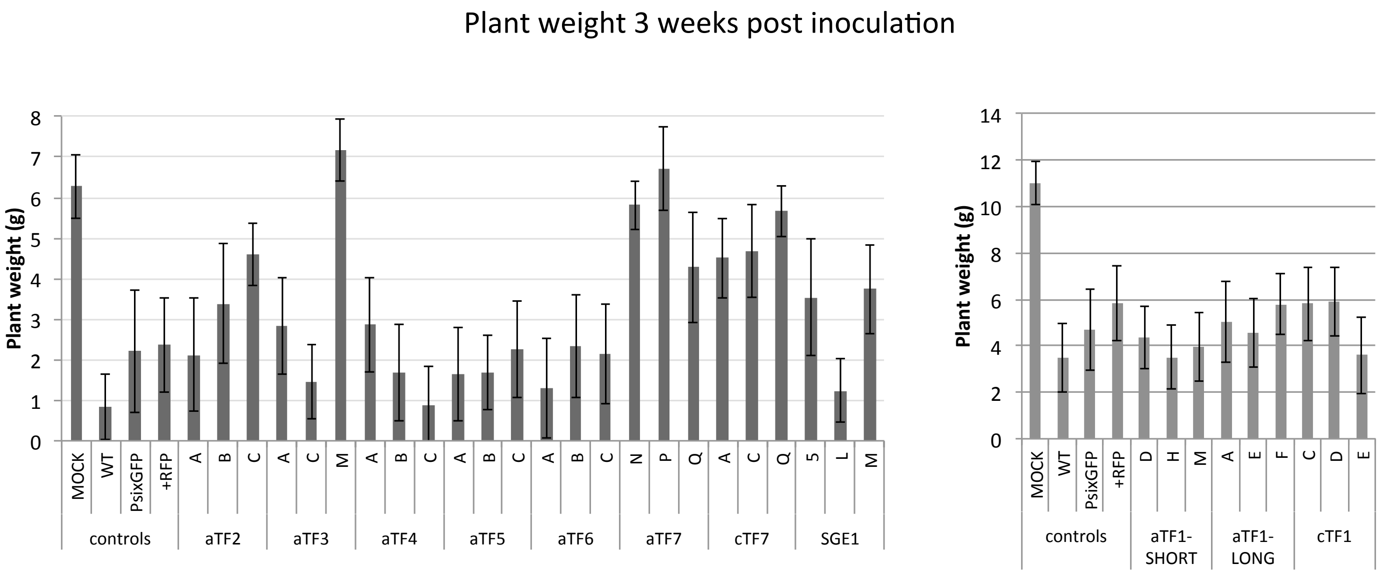

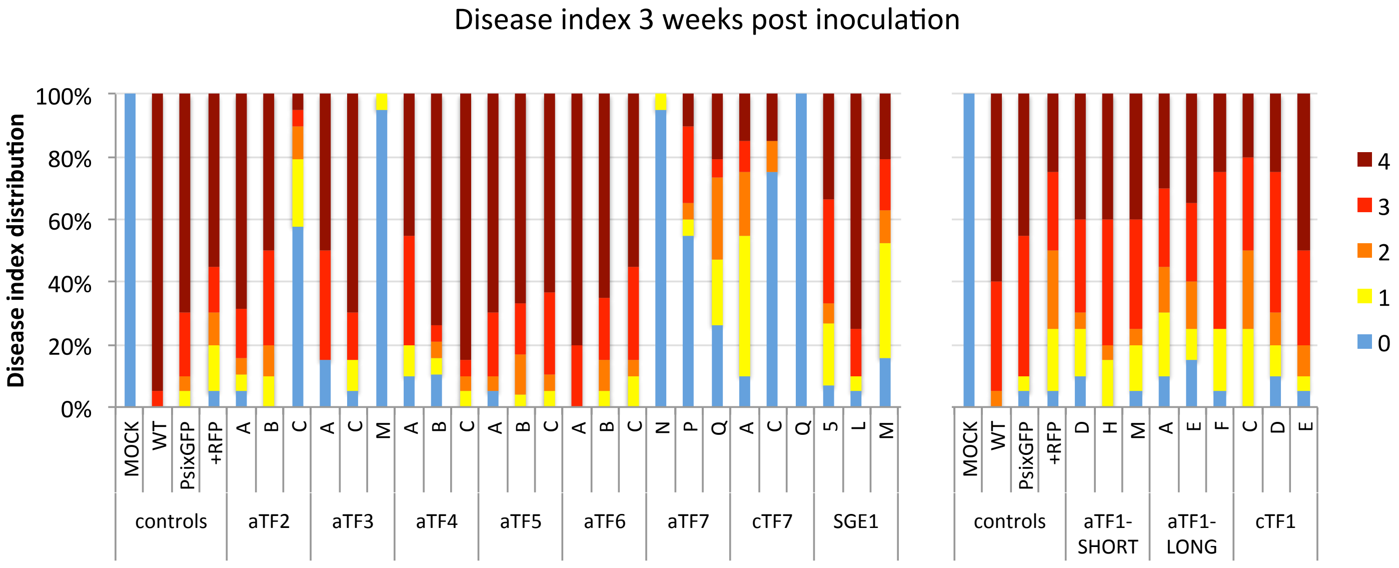

SUPP FIGURE 5D
