# Supplementary material for: Transcription Factors Encoded on Core and Accessory Chromosomes of Fusarium oxysporum Induce Expression of Effector Genes
Source: PLoS Genet. 2016 Nov 17;12(11):e1006401. doi: 10.1371/journal.pgen.1006401 (PMC5140021; doi:10.1371/journal.pgen.1006401)
Supplement: S6 Fig — A) Top three Venn diagrams show the overlap of sets of differentially expressed genes between two independent transformants of each transcription factor. The overlapping genes are depicted in red and these are the genes considered for all further analyses. Lower six Venn diagrams: overlap between target genes in the different overexpressors, up-regulated (left three diagrams) or down-regulated (right three diagrams). B&C) Number of up- or down-regulated genes in different subcategories. Significant enrichment or depletion in each subcategory was tested with a hypergeometric test, P value < 0.01 after Bonferroni correction. Orange cells: enrichment, blue cells: depletion. Columns: whole genome (all genes), planta UP (genes up-regulated during infection), aTF1 UP, cTF1 UP, SGE1 UP (genes up-regulated in the aTF1, cTF1 or SGE1 overexpressors, a1c1S1 UP (genes up-regulated in all three overexpressors), planta DOWN (genes down-regulated during infection), aTF1 DOWN, cTF1 DOWN, SGE1 DOWN (genes down-regulated in the aTF1, cTF1 or SGE1 overexpressors, a1c1S1 DOWN (genes down-regulated in all three overexpressors), MIMP (genes with a miniature impala transposable element (MIMP) in their promoter), XS proteins (genes of which the protein product was found in the xylem sap of infected tomato plants [34]). Rows: all (all genes), core (all genes located on the core genome), pathogenicity chr. (all genes on the pathogenicity chromosome), other accessory chr. (all accessory genes, except those on the pathogenicity chromosome), in planta UP all (all genes up-regulated during infection), UP core (genes located on core chromosomes and up-regulated during infection), UP pathogenicity chr. (genes located on the pathogenicity chromosome and up-regulated during infection), UP accessory chr. (genes on the accessory regions, except those on the pathogenicity chromosome, and up-regulated during infection), in planta DOWN (all genes down-regulated during infection), SIX genes (all SIX genes [file pgen.1006401.s006.pdf]

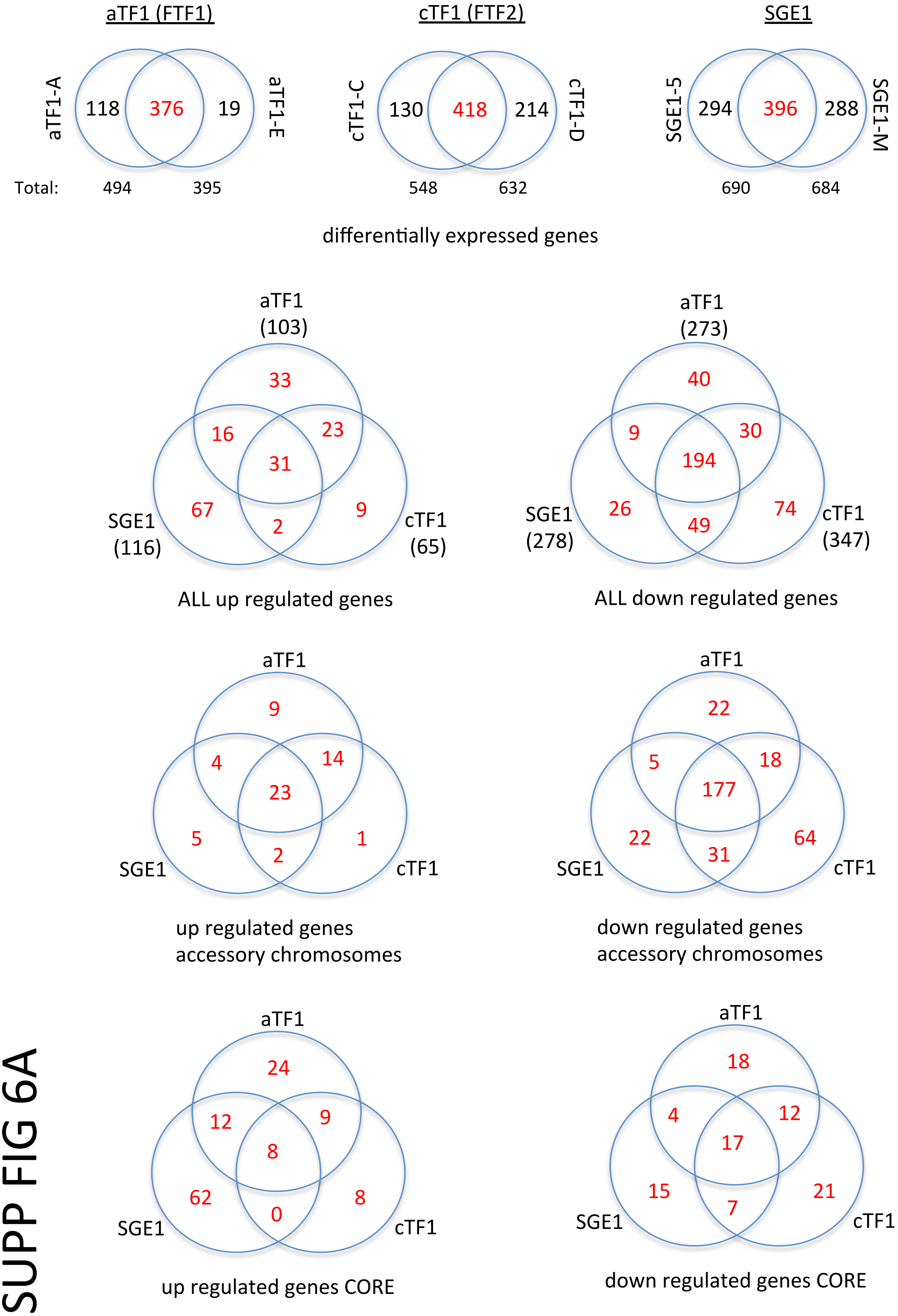
aTF1 (FTF1)
aTF1-A
118
376
19
aTF1-E
Total:
494
395
cTF1 (FTF2)
cTF1-C
130
418
214
cTF1-D
548
632
SGE1
SGE1-5
294
396
288
SGE1-M
690
684
differentially expressed genes
aTF1 (103)
33
16
23
31
SGE1 (116)
67
2
9
cTF1 (65)
ALL up regulated genes
aTF1 (273)
40
9
30
194
SGE1 (278)
26
49
74
cTF1 (347)
ALL down regulated genes
aTF1
9
4
14
23
SGE1
5
2
1
cTF1
up regulated genes accessory chromosomes
aTF1
22
5
18
177
SGE1
22
31
64
cTF1
down regulated genes accessory chromosomes
aTF1
24
12
9
8
SGE1
62
0
8
cTF1
up regulated genes CORE
aTF1
18
4
12
17
SGE1
15
7
21
cTF1
down regulated genes CORE

SUPP FIG 6A

B

NUMBER OF GENES		whole genome	planta UP	aTF1 UP	cTF1 UP	SGE1 UP	a1c1S1 UP	planta DOWN	aTF1 DOWN	cTF1 DOWN	SGE1 DOWN	a1c1S1 DOWN
LOCATION	all	20934	1412	103	65	116	31	443	273	347	278	194
	core	16176	1129	53	25	82	8	394	51	57	43	17
	pathogenicity chr.	436	81	36	30	29	22	1	10	7	9	5
	other accessory chr.	4322	202	14	10	5	1	48	212	283	226	172
IN PLANTA	*in planta* UP all	1412	1412	75	50	50	27	0	15	15	19	4
	UP core	1129	1129	25	13	20	nd	nd	nd	nd	nd	nd
	UP pathogenicity chr.	81	81	36	29	29	nd	nd	nd	nd	nd	nd
	UP other accessory chr.	202	202	14	8	1	nd	nd	nd	nd	nd	nd
	in planta DOWN	443	0	2	0	4	0	443	5	6	2	1
XS PROTEINS	SIX genes	18	14	14	14	11	11	0	0	0	0	0
	XS proteins	55	40	24	16	21	13	1	0	0	0	0
	XS proteins w/o SIX proteins	41	26	10	2	10	2	1	0	0	0	0
MIMP	mimp	73	40	27	23	19	17	0	0	0	0	0
	mimp on accessory chr.	69	40	27	23	19	17	0	0	0	0	0
	mimp onpathogenicity chr.	41	30	25	22	19	17	0	0	0	0	0
	mimp on core	4	0	0	0	0	0	0	0	0	0	0
	mimp w/o SIXgenes	55	26	13	9	8	6	0	0	0	0	0
	mimp on path. chr. w/o SIX	28	17	12	9	8	7	0	0	0	0	0

C

NUMBER OF GENES		MIMP	XS PROTEINS
LOCATION	all	73	55
	core	4	36
	pathogenicity chr.	41	16
	other accessory chr.	28	3
IN PLANTA	*in planta* UP all	40	40
	UP core	nd	nd
	UP pathogenicity chr.	nd	nd
	UP other accessory chr.	nd	nd
	in planta DOWN	0	1
XS PROTEINS	SIX genes	18	14
	XS proteins	16	55
	XS proteins w/o SIX proteins	2	41
MIMP	mimp	73	16
	mimp on accessory chr.	69	16
	mimp onpathogenicity chr.	41	14
	mimp on core	4	0
	mimp w/o SIXgenes	55	2
	mimp on path. chr. w/o SIX	28	1

D

			UP							DOWN						
		ALL	aTF1A	aTF1E	cTF1C	cTF1D	SGE1 5	SGE1M	in planta	aTF1A	aTF1E	cTF1C	cTF1D	SGE1 5	SGE1M	in planta
compare to:	ALL genes	20934	129	112	111	165	252	324	1412	366	284	438	467	439	360	443
	accessory	4758	54	51	46	109	93	62	283	291	228	348	334	322	264	49
	TE	3888	37	27	18	50	59	34	212	242	281	308	253	246	192	79

			UP							DOWN						
		ALL	aTF1A	aTF1E	cTF1C	cTF1D	SGE1 5	SGE1M	in planta	aTF1A	aTF1E	cTF1C	cTF1D	SGE1 5	SGE1M	in planta
compare to:	accessory	4758	54	51	46	109	93	62	283	291	228	348	334	322	264	49
	TE	3888	37	27	18	50	59	34	212	242	281	308	253	246	192	79

SUPP FIG 6
