# Supplementary material for: Correction: Transcription Factors Encoded on Core and Accessory Chromosomes of Fusarium oxysporum Induce Expression of Effector Genes
Source: PLoS Genet. 2016 Dec 29;12(12):e1006527. doi: 10.1371/journal.pgen.1006527 (PMC5199062; doi:10.1371/journal.pgen.1006527)
Supplement: S2 File — (PDF) [file pgen.1006527.s002.pdf]

PLOS | GENETICS

RESEARCH ARTICLE

Transcription Factors Encoded on Core and Accessory Chromosomes of *Fusarium oxysporum* Induce Expression of Effector Genes

H. Charlotte van der Does[1], Like Fokkens[1], Ally Yang[2], Sarah M. Schmidt[1¤], Léon Langereis[1], Joanna M. Lukasiewicz[1], Timothy R. Hughes[2], Martijn Rep[1]*

1 Molecular Plant Pathology, University of Amsterdam, The Netherlands, **2** Banting and Best Department of Medical Research, University of Toronto, Toronto, ON, Canada

¤ Current address: The Sainsbury Laboratory, Norwich Research Park, Norwich
* m.rep@uva.nl

CrossMark
click for updates

OPEN ACCESS

Citation: van der Does HC, Fokkens L, Yang A, Schmidt SM, Langereis L, Lukasiewicz JM, et al. (2016) Transcription Factors Encoded on Core and Accessory Chromosomes of *Fusarium oxysporum* Induce Expression of Effector Genes. PLoS Genet 12(11): e1006401. doi:10.1371/journal.pgen.1006401

Editor: Eva H. Stukenbrock, Max-Planck-Institut fur Evolutionsbiologie, GERMANY

Received: February 17, 2016

Accepted: October 4, 2016

Published: November 17, 2016

Copyright: © 2016 van der Does et al. This is an open access article distributed under the terms of the Creative Commons Attribution License, which permits unrestricted use, distribution, and reproduction in any medium, provided the original author and source are credited.

Data Availability Statement: All relevant data are within the paper and its Supporting Information files.

Funding: This work was supported by the Netherlands Organization for Scientific Research (nwo.nl) through Veni grant 016.121.067 to HCvdD. The funder had no role in study design, data collection and analysis, decision to publish, or preparation of the manuscript.

Abstract

Proteins secreted by pathogens during host colonization largely determine the outcome of pathogen-host interactions and are commonly called 'effectors'. In fungal plant pathogens, coordinated transcriptional up-regulation of effector genes is a key feature of pathogenesis and effectors are often encoded in genomic regions with distinct repeat content, histone code and rate of evolution. In the tomato pathogen *Fusarium oxysporum* f. sp. *lycopersici* (Fol), effector genes reside on one of four accessory chromosomes, known as the 'pathogenicity' chromosome, which can be exchanged between strains through horizontal transfer. The three other accessory chromosomes in the Fol reference strain may also be important for virulence towards tomato. Expression of effector genes in Fol is highly up-regulated upon infection and requires Sge1, a transcription factor encoded on the core genome. Interestingly, the pathogenicity chromosome itself contains 13 predicted transcription factor genes and for all except one, there is a homolog on the core genome. We determined DNA binding specificity for nine transcription factors using oligonucleotide arrays. The binding sites for homologous transcription factors were highly similar, suggesting that extensive neofunctionalization of DNA binding specificity has not occurred. Several DNA binding sites are enriched on accessory chromosomes, and expression of *FTF1*, its core homolog *FTF2* and *SGE1* from a constitutive promoter can induce expression of effector genes. The DNA binding sites of only these three transcription factors are enriched among genes up-regulated during infection. We further show that Ftf1, Ftf2 and Sge1 can activate transcription from their binding sites in yeast. RNAseq analysis revealed that in strains with constitutive expression of *FTF1*, *FTF2* or *SGE1*, expression of a similar set of plant-responsive genes on the pathogenicity chromosome is induced, including most effector genes. We conclude that the Fol pathogenicity chromosome may be partially transcriptionally autonomous, but there are also extensive transcriptional connections between core and accessory chromosomes.

Competing Interests: The authors have declared that no competing interests exist.

Author Summary

Eukaryotic genomes are organised. Genomic regions may differ in spatial organisation, chromatin condensation, rate of evolution, GC content, gene expression and transposon density. Many plant pathogenic fungi maintain genomic subcompartments containing specialized genes to facilitate host colonisation. These genes, for example effector genes, show concerted transcriptional up-regulation during infection. An extreme case is the tomato pathogen *Fusarium oxysporum* f. sp. *lycopersici*, which carries accessory chromosomes, of which one encodes all effector genes and can be transferred horizontally between strains. We investigated the transcriptional connections between this accessory chromosome and the core genome, particularly with respect to effector gene expression. Several of the transcription factors encoded on this accessory chromosome bind to motifs enriched on accessory chromosomes, suggesting the accessory chromosomes may be partially transcriptionally independent. Only one of these, Ftf1, can induce the expression of effector genes and binds to a motif that is enriched in their promoters. Also Sge1 –a conserved regulator of fungal lifestyle switches and required for infection–can activate the expression of effector genes. Both transcription factors induce a largely overlapping set of genes, including many of the host-induced genes on the accessory chromosome including the effector genes. This demonstrates the existence of extensive transcriptional connections between accessory and core chromosomes.

Introduction

Plant pathogenic fungi are genetically adapted to infect their host plant, but are also in a constant arms race with that host to stay virulent. For this, pathogens need to allow accelerated evolution of pathogenicity-related genes, without affecting the function of housekeeping genes. One possibility is to spatially separate these different functional groups of genes into subgenomic compartments with different rates of evolution. One of the fastest evolving determinants of pathogenicity is the effector repertoire. Definitions vary, but a typical effector is a small, secreted protein that affects the interaction between the pathogen and its host. In many plant pathogenic fungi effector genes indeed reside in specific genomic regions, generally distinguished by one or more of the following characteristics: lineage-specific or accessory, rich in transposable elements, a different GC content and/or codon bias from the rest of the genome, depleted for housekeeping genes and associated with particular chromatin modifications [1–3]. Accumulating evidence suggests that these genomic environments evolve more rapidly than the rest of the genome and facilitate adaptation [2,4,5]. In addition, in several fungi these types of regions—or parts thereof—have been shown or suggested to transfer horizontally between different strains or even species [6–11].

Another hallmark of effector genes is a plant specific expression pattern [12–14]. How coordinated expression of effector genes is regulated and how this is related to the specific genomic environment of these genes is poorly understood. For some effector genes it has been shown that their genomic environment is key for regulated expression, through histone modifications [1,15]. On the other hand, a small number of transcription factors required for effector gene expression has been identified. In *Ustilago maydis* the heterodimer bE/bW, the forkhead transcription factor Fox1, and the zinc finger transcription factors Rbf1 and Mzr1 are involved in transcriptional regulation of pathogenicity-related genes and/or effector genes [16–19]. In *Leptosphaeria maculans* and *Stagnospora nodorum*, homologs of StuA, a bHLH (basic helix-loop-

helix) type of transcription factor, regulate expression of several effector genes [20,21]. Most is known about the role of Wor1 orthologs in effector gene expression. Wor1 is a conserved fungal transcription factor from *Candida albicans*, with a WOPR type of DNA binding domain [22]. In plant pathogenic fungi from the genus *Fusarium* (putative) effector genes and/or secondary metabolite gene clusters are regulated by an ortholog of this transcription factor: *F. oxysporum* f. sp. *lycopersici* (Sge1), *F. graminearum* (Fgp1), *F. verticillioides* (FvSge1) and *F. fujikuroi* (FfSge1) [23–26]. Also in the plant pathogenic fungi *Botrytis cinerea* (Reg1), *Verticillium dahliae* (VdSge1), *Cladosporium fulvum* (CfWor1), *Zymoseptoria tritici* (ZtWor1), *Ustilago maydis* (UmRos1) and *Magnaporthe oryzae* (MoGti1) deletion of the gene for this transcription factor (partially) perturbs expression of effector genes [27–32]. Mutant strains deleted for this gene are mostly non-pathogenic (except Δffsge1), although for *CfWOR1* this may be a secondary effect of a developmental phenotype. In *C. albicans*, Wor1 was originally discovered as a 'master regulator' of the morphological switch from white to opaque cells. Also in *Saccharomyces cerevisiae* and *Histoplasma capsulatum* the Wor1 orthologs (Mit1 and Ryp1, respectively) regulate a morphological transition, which, both in *C. albicans* and *H. capsulatum*, is associated with differences in virulence towards humans [33]. This led to the idea that Wor1 orthologs in plant pathogenic fungi are also master regulators of a lifestyle switch, from saprotrophic to pathogenic.

In *Fusarium oxysporum* f. sp. *lycopersici* (Fol), the causal agent of Fusarium wilt in tomato, effector genes (called *SIX* genes for '*Secreted In Xylem*') reside on an accessory chromosome that can be transferred horizontally between strains. Upon receipt of this accessory chromosome of Fol, a non-pathogenic strain can acquire pathogenicity towards tomato [7]. This means that effector gene expression must be ensured in different genomic environments (i.e. in the original strain and in the recipient strain). Two different but not mutually exclusive strategies to ensure effector gene expression are: i) to rely on conserved transcription factors encoded on the core genome or ii) to encode the transcription factors necessary for effector gene expression on the accessory chromosome itself. As mentioned above, Fol effector gene expression requires the presence of the core-encoded conserved transcription factor Sge1 [26]. However, also on the accessory chromosome transcription factors are encoded, 13 in total [34]. One of these transcription factor genes, *FTF1*, is associated with highly virulent strains of *F. oxysporum* f. sp. *phaseoli* and is up-regulated during infection [35,36]. In addition, *FTF1* is present in three variants on the Fol pathogenicity chromosome and all three genes are located close to single or small groups of effector genes [34].

Although the pathogenicity chromosome of Fol is transcriptionally connected to the core genome via Sge1, the presence of numerous transcription factor genes on the chromosome itself suggests that this accessory chromosome might be transcriptionally semi-autonomous. To see whether this may be the case, we investigated the role of the transcription factors encoded on the pathogenicity chromosome of Fol in effector gene expression.

Results

The pathogenicity chromosome of Fol encodes nine transcription factor gene families, of which four comprise multiple genes on accessory chromosomes

To see if the pathogenicity chromosome of Fol (chromosome 14 in reference strain Fol4287) may be transcriptionally autonomous, we inventoried the transcription factors it encodes. We found 13 predicted transcription factor genes that cluster into nine families. The transcription factor gene families were numbered *TF1* to *TF9* and include one homolog of *EBR1* (*TF8*) and three homologs of *FTF1* (*TF1*) (**Fig 1, S1 Data: tab 'TF table'**) [35,37]. Most gene families encode proteins containing zinc finger DNA binding domains; four are Cys2His2 zinc finger

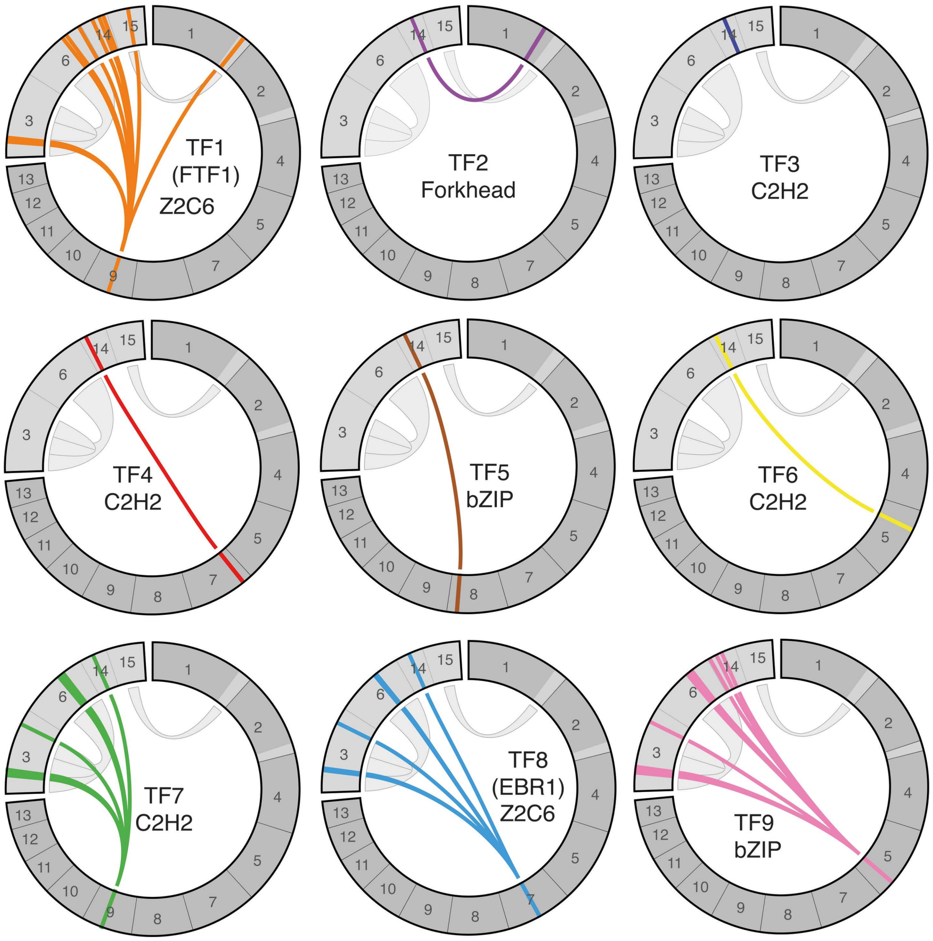

Fig 1. Transcription factor genes on the pathogenicity chromosome have homologs on the core genome and on other accessory chromosomes. Schematic representation of the chromosomes of Fol and the position of transcription factor genes on the pathogenicity chromosome (chromosome 14) and their homologs on other chromosomes. All accessory regions are depicted in light grey and core chromosomes in dark grey. Note that small accessory regions are attached to core chromosomes 1 and 2. Grey ribbons indicate duplicated genomic regions. Transcription factor genes are indicated with colored bars, each transcription factor gene family is represented in a separate circos plot.

doi:10.1371/journal.pgen.1006401.g001

DNA binding domains (Tf3, Tf4, Tf6 and Tf7) and two are Zn(2)Cys(6) zinc finger DNA binding domains (Tf1 and Tf8). Additionally, there are two gene families encoding transcription factors with a basic leucine zipper (bZIP) DNA binding domain (Tf5 and Tf9) and one gene family encoding forkhead transcription factors (Tf2). All transcription factor genes on the pathogenicity chromosome have a homolog on the core genome, except *TF3* (Fig 1). Four of the transcription factor gene families have also expanded on other accessory chromosomes of Fol4287 (*TF1*, *TF7*, *TF8* and *TF9*).

The *F*. *oxysporum* species complex encompasses many different *formae speciales* (*ff.spp*.), each with a specific plant host and specific accessory genomic material. All transcription factor gene families (except *TF3*) have one core- and one or more accessory-encoded homologs in other *ff*. *spp*. of *F*. *oxysporum* investigated. (Fig 2, S1 Fig, S1 Data:tab **'TF table'**). From here on, we refer to accessory homologs as aTF and to core homologs as cTF. The other *Fusarium* species analysed (*F*. *solani*, *F*. *graminearum* and *F*. *verticillioides*) each have only one homolog of most of the transcription factors; the expansion we see on the accessory regions in *F*.

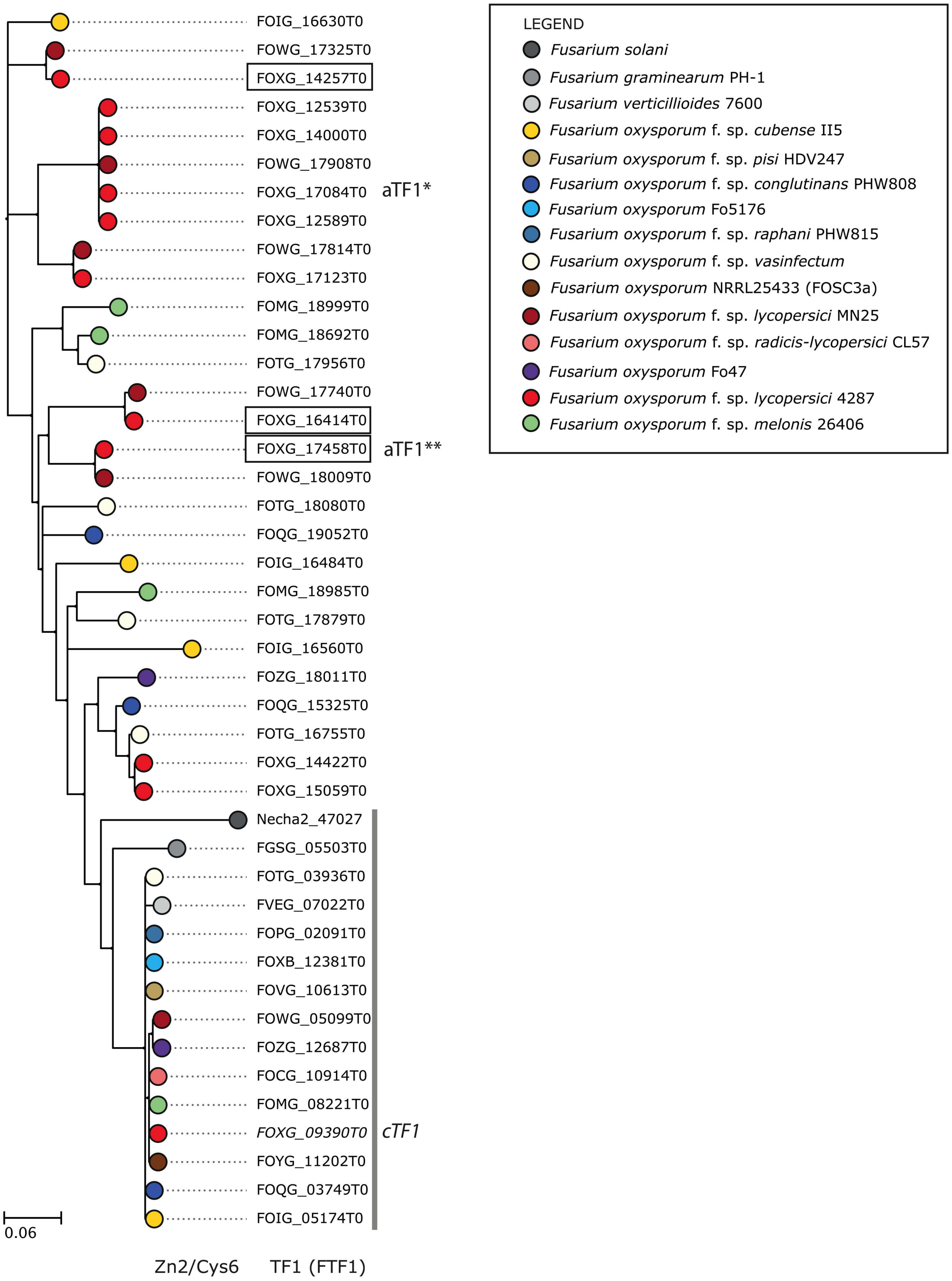

Fig 2. Transcription factors encoded on accessory chromosomes of *F. oxysporum* have diverged more than core-encoded transcription factors between *Fusarium* species. Phylogenetic tree of the Tf1 (Ftf1) family based on protein sequence alignments, including homologs in Fol, other *F. oxysporum ff. spp.* and other *Fusarium* species. The aTf1 homologs encoded on the pathogenicity chromosome in Fol are boxed, and the core homolog (cTf1) is written in italics. Cloned genes are indicated with *aTF1* or *cTF1*. *aTF1**: short version of *aTF1*, *aTF1***: long version of *aTF1*. A grey bar indicates the clade with Fol, Fv, Fg and Fs core-encoded homologs. Phylogenetic trees of the other transcription factor families are presented in S1 Fig.

doi:10.1371/journal.pgen.1006401.g002

oxysporum has not occurred. Regardless of the *forma specialis*, core-encoded transcription factors form one clade (**Fig 2**, **S1 Fig**, indicated with a grey bar) and show little sequence divergence. In general, the *F. verticillioides* homologs are closest to this clade, consistent with the species phylogeny. Accessory chromosome-encoded homologs show more divergence, both within and between strains, and are also more diverse in sequence than the core encoded homologs in *F. oxysporum* and *F. verticillioides*. This suggests that either i) the expansion and divergence of the transcription factor genes on the accessory chromosomes is older than the separation between *F. oxysporum* and *F. verticillioides*, similar to what was suggested for accessory genes in general [7], or ii) the rate of evolution is accelerated in the accessory regions compared to the core genome, or iii) a combination of the two.

DNA binding specificity is conserved between homologous transcription factors

As transcription factor gene families have expanded and diverged on the accessory chromosomes, we wanted to investigate whether some of these genes may have neofunctionalized. To see if homologous core- and accessory-encoded transcription factors regulate different target genes, we set out to determine the DNA binding sites of each transcription factor encoded on the pathogenicity chromosome and its core-encoded homolog.

Transcription factor coding sequences were cloned from cDNA, *in vitro* translated as a GST-fusion and hybridised with two different oligo arrays (called HK & ME) [38]. Binding enrichments were inferred for each possible 8-mer. Of 13 transcription factors the cDNA could be amplified and cloned. For aTf1, aTf8 and aTf9, cloning of any of the homologs on the pathogenicity chromosome was unsuccessful. Possibly this was due to both low transcript levels and the presence of homologous transcripts; only hybrid PCR products were amplified. However, a partial cDNA encoding the predicted DNA binding domain from another aTf1 homolog was obtained (FOXG_17084 on accessory chromosome 6). Tf1 homologs separate in two groups based on the length of the coding sequence. The first group (~3200 bp, we refer to this as the longer coding sequence) include the core homolog, two homologs on the pathogenicity chromosome and two identical genes on other accessory chromosomes. The remaining *aTF1* genes are shorter (~2800 bp), because they have a more downstream startcodon and a more upstream stopcodon (**S2 Fig**) [35]. All Tf1 homologs have highly similar DNA binding domains (**S3 Fig**). The cloned *aTF1* cDNA (FOXG_17084) has a short coding sequence.

For nine of the cloned transcription factors a reliable DNA binding site could be inferred from one or both arrays (**Fig 3A**, **S1 Data**: **tab 'DNA binding assay'**). In all cases both arrays yielded similar top 8-mers. For the remaining four transcription factors no significant enrichment was found. The DNA binding sites of homologous transcription factors were the same or very similar in all four families for which both homologs yielded a DNA binding site, indicating that no diversification in recognition specificity has occurred. The DNA binding site of aTf2 and its homolog cTf2 overlap with the consensus DNA binding site of forkhead transcription factors (RYMAAYA) [39]. The aTF5/cTF5 DNA binding site is almost palindromic, which is in accordance with the dimeric structure of leucine zippers. Tf1 has a Gal4-like Zn(2)Cys(6) DNA binding domain, which in Gal4 binds as a dimer to the CGGN$_{11}$CCG consensus sequence [40]. The DNA binding site of aTf1 and cTf1 (TRCCG) overlaps with half of this consensus. Interestingly, the aTf1 DNA binding site overlaps with a motif found earlier to be enriched in the promoters of effector genes: AACTGCCGA [34]. Note that the top 8-mer for both aTf1 and cTf1 is perfectly contained in this motif (**Fig 3A**, **S1 Data**: **tab 'DNA binding assay'**).

We attempted to determine the DNA binding site of Sge1 in a similar way, but no significant 8-mer enrichments were detected. Sge1 has an unusual fungi-specific WOPR type DNA

A

	Accessory (path. chr.)	Core	
aTF1	AACTGCCG<br>AACTGCCG	AACTGCCG<br>AATTGCCG	cTF1
aTF2	ATCAAACA<br>ATCAAACA	GACAAACA<br>AGCAAACA	cTF2
aTF3	no s.b.		
aTF4	no s.b.	CAGCCCTA<br>CAGCCCTA	cTF4
aTF5	ACGTGTCC<br>ACACGTGT	ACGTGTCC<br>ACACGTGT	cTF5
aTF6	no s.b.	no s.b.	cTF6
aTF7	GAGCCTCA<br>GAGCCGCA	GAGCCGCA<br>GAGCCGCA	cTF7
aTF8	nd	nd	cTF8
aTF9	nd	nd	cTF9
		no s.b.	SGE1

Motif

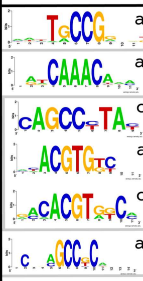

B

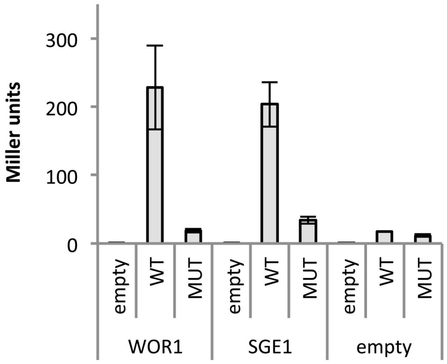

WT TCAAATTAAGGTTT
MUT TCAAATTCAGGACT

C

Number of genes with ≥1 TF DBS in the 1000 bp upstream the transcriptional startsite

TF	motif		whole genome				*in planta* regulated genes			
							UP		DOWN	
			all	core	accessory	path.chr.	all	path.chr.	all	path.chr.
aTF1 (FTF1)		fwd	15351	11747	**3604**	332	**726**	50	105	0
	T[G,A]CCG	rev	15399	11884	3515	323	700	50	115	0
effector motif		fwd	136	119	17	2	**20**	1	0	0
	AACTGCCGA	rev	141	119	22	3	14	3	0	0
aTF2		fwd	13664	10654	3010	284	602	39	88	0
	CAAAC	rev	14250	**11366**	2884	293	604	47	94	0
cTF4		fwd	3650	2681	**969**	85	155	4	26	0
	AGCC[A,G,C,T]TA	rev	3610	2666	**944**	79	156	10	26	0
aTF5		fwd	9017	6840	**2177**	210	403	25	62	0
	CACGT	rev	9292	7110	2182	209	393	29	68	0
aTF7		fwd	10982	8497	2485	208	453	25	75	0
	G[A,C,G]GGCT	rev	10460	8063	2397	216	426	23	73	0
SGE1		fwd	2749	2034	**715**	74	**178**	**21**	19	0
	TTA[A,G][A,G][G,C]TT	rev	2770	2022	**748**	69	**179**	19	16	0
	**all genes**		20932	16174	4758	436	896	63	140	0

Fig 3. DNA binding motifs are not diversified within transcription factor families, but some motifs are enriched on accessory chromosomes. A) DNA binding motifs determined by DNA binding arrays for several accessory and core transcription factor pairs. Accessory transcription factors were cloned from the pathogenicity chromosome in all cases except *aTF1*; this transcription factor was cloned from another accessory chromosome. Of transcription factors with a reliable DNA binding site on one or two arrays, the top 8-mers for both arrays are given (in color: significant enriched binding [E-score > 0.45], in grey, E-score < 0.45). For each transcription factor: upper 8-mer: HK array, lower 8-mer: ME array. For transcription factors with significantly enriched binding to ten 8-mers or more, a motif was constructed of all 8-mers with significant binding (right hand side of the figure). **B**) Sge1 can transcriptionally activate an UAS-less *CYC1* promoter via the presence of the Wor1 DNA binding site. Activation is lost when the Wor1 DNA binding site is absent or mutated (lower panel). Activation assays were performed in duplicate, error bars represent standard deviation. **C)** The number of genes with one or more transcription factor DNA binding site (TF DBS) in the promoter, for the complete genome, for a subgenome (core, accessory or pathogenicity chromosome), and for a subset of genes (up- or down-regulated during infection). Boxes indicate a significant enrichment (P value < 0.01 after Bonferroni correction). The motif found in effector promoters earlier (AACTGCCGA) and overlapping with the Tf1 DNA binding site has been included.

doi:10.1371/journal.pgen.1006401.g003

binding domain. The DNA binding site and protein crystal structure of several homologs of Sge1 have been resolved [33,41–43]. The amino acids interacting with DNA are highly conserved among Wor1/Sge1 homologs from different fungi [23,27,29,30,41,43,44]. Consistent

with this, the DNA binding sites of Sge1 orthologs in *Saccharomyces cerevisiae* (Mit1) and the filamentous fungus *Histoplasma capsulatum* (Ryp1) are the same as for Wor1 [33].

To test whether Sge1 can also bind to the Wor1-DNA binding site, we have adopted an *in vivo* transcriptional activation assay developed previously [42]. In short, Wor1 or Sge1 is produced constitutively in yeast together with a reporter construct consisting of the Wor1-DNA binding site and an UAS-less *CYC1* promoter fused to the *lacZ* gene. The two *SGE1* homologs in yeast (YEL007 or Mit1 and YHR177) are deleted from the yeast strain to avoid potential cross-activation. We found that Sge1 can induce the reporter gene to the same level as Wor1, and this ability is lost when the Wor1 DNA-binding site is mutated (**Fig 3B**). This confirms that Sge1 binds the same DNA sequence as its orthologs and is a transcriptional activator.

Accessory chromosomes are enriched for DNA binding sites of some transcription factors encoded on the pathogenicity chromosome

Determination of the DNA binding site of several transcription factors showed that DNA binding specificity has not or hardly diverged between accessory-encoded and core-encoded transcription factors. However, if accessory-encoded transcription factor homologs fulfill an important role in transcriptional regulation of the accessory chromosomes, target genes of these transcription factors could be enriched there. To test this, the number of genes with minimally one, two or three binding sites in 1 kb upstream of the annotated transcriptional start site was counted (Fusarium Comparative Sequencing Project, Broad Institute of Harvard and MIT (http://www.broadinstitute.org/), annotation 3) (**S1 Data: tab 'promoter DBS'**). Because some of the sequence motifs contain relatively little information we found many occurrences throughout the genome. Nevertheless, for several DNA binding sites (of aTf1, cTf4 and aTf5) significant enrichment (hypergeometric test, P value < 0.01 after Bonferroni correction) was found on accessory chromosomes, and for the aTf2 DNA binding site enrichment was found on the core genome (**Fig 3C, S1 Data: tab 'promoter DBS'**). Specific enrichment of DNA binding sites on the pathogenicity chromosome, however, was not detected. In an effort to reduce noise levels for the smaller sequence motifs we also looked at multiple occurrences, and indeed the observed enrichments were also present under these criteria. Interestingly, also the Sge1 DNA binding site is enriched on the accessory chromosomes. To test whether the observed enrichments are specific for promoter regions, we performed the same test for the 1000 bp downstream the ATG of each gene. We found no DNA binding site enrichments in the coding regions, except for the aTf1 DNA binding site, which was found enriched both in the promoter and in the coding regions of accessory genes (**S4A and S4B Fig, S1 Data: tab 'ORF DBS'**).

Since the pathogenicity chromosome and the effectors encoded on it are important for the infection of tomato plants, enrichment of DNA binding sites among genes that are up-regulated during infection and those that are down-regulated was also tested [45]. Only the aTf1 and the Sge1 DNA binding sites were significantly enriched among up-regulated genes, and none of the DNA binding sites was enriched among genes down-regulated during infection (**Fig 3C, S1 Data**).

Taken together, this statistical analysis suggests that several transcription factor gene families on the pathogenicity chromosome may be involved in regulating targets on accessory chromosomes. In addition, aTf1 and/or cTf1 and Sge1 in particular may target genes involved in pathogenicity.

Expression of *aTF1*, *cTF1* or *SGE1* from a constitutive promoter induces *SIX1* expression

The effectors encoded on the pathogenicity chromosome are important determinants for pathogenicity and are under strict transcriptional regulation [26,46–50]. To determine whether

transcription factors encoded on the pathogenicity chromosome can induce effector gene expression, strains ectopically expressing these transcription factors from a constitutive promoter—from hereon called 'overexpressors'–were generated and tested for their ability to induce effector gene expression. For seven out of the nine transcription factor families encoded on the pathogenicity chromosome we were able to make constructs with the constitutive *FEM1* promoter [51] to drive expression (cloning of *aTF8* and *aTF9* was unsuccessful). Three *aTF1* homologs are present on the pathogenicity chromosome and one of these was selected for overexpression (FOXG_17458, long coding sequence). Also one 'short' *aTF1* gene from another accessory region was chosen for overexpression (FOXG_17084), the same gene that was used for the DNA binding site determination.

To facilitate screening for the induction of effector gene expression, transcription factor overexpression constructs were transformed into a strain carrying a reporter gene. In this Fol strain, the reporter (*GFP*) replaces the ORF of the effector gene *SIX1* –a representative plant-induced effector gene–so that it is expressed *in locus* from the *SIX1* promoter [46]. For each transcription factor, 11 to 23 independent transformants were inspected for increase of *GFP* expression using fluorescence microscopy (**S5A Fig**). GFP signals varied between transformants with the same construct. The three to ten transformants that appeared to respond most strongly to each transcription factor were used to quantify GFP levels spectrophotometrically (**Fig 4**). Most accessory transcription factors tested did not affect *SIX1* expression when expressed from the *FEM1* promoter. In contrast, the long version of *aTF1* very strongly induced *SIX1* expression, and the shorter version conferred some induction. Also *SGE1*, tested in the same way, can induce *SIX1* expression, albeit to a lesser extent than *aTF1*. To confirm overexpression of the transcription factor genes in the transformants that showed no *GFP* induction, transcript levels were determined with quantitative RT-PCR. In all cases an increase of transcription factor gene expression compared to the background strain was observed in at least one out of three transformants tested (**S5B Fig**).

Some of the strains expressing *SGE1* or the long version of *aTF1* from the *FEM1* promoter showed slight growth retardation, and strains overexpressing *aTF7* (FOXG_14275) consistently grow slower on both rich and minimal medium (**S5C Fig**). The same transformants

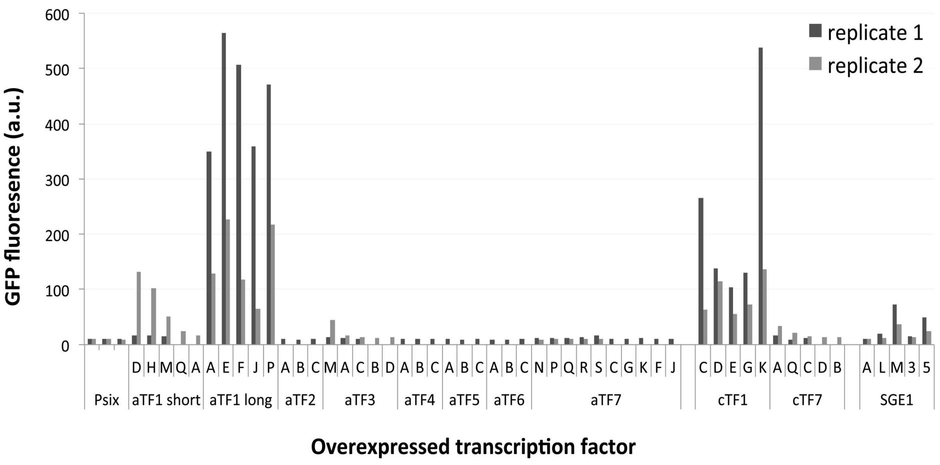

Fig 4. Expression of *aTF1* (*FTF1*), *cTF1* (*FTF2*) or *SGE1* from a constitutive promoter activates the *SIX1* promoter. Quantitative measurement of GFP fluorescence in strains expressing transcription factor genes from the *FEM1* promoter. For each transcription factor fluorescence of 2*10^6 spores was measured in several independent transformants, as indicated with letters or numbers. Psix1 is the background strain (Fol007 with the *Psix1GFP* reporter construct).

doi:10.1371/journal.pgen.1006401.g004

were tested for their ability to infect tomato plants. Only *aTF7* overexpression altered this ability; *aTF7*-overexpressors were less virulent, which may be explained by their retarded growth (S5D Fig).

To see whether the phenotypes caused by *aTF1* or *aTF7* overexpression could also be induced by overexpression of their core homologs, strains expressing *cTF1* (FOXG_09390, long gene model) or *cTF7* (FOXG_17774) from the *FEM1* promoter were generated and tested as described above (S5 Fig, Fig 4). *cTF1* can activate the *SIX1* promoter almost to the levels of the long version of *aTF1*, and *cTF7* overexpressors have a similar growth retardation and reduced virulence phenotype as those overexpressing *aTF7*. This suggests a similar function for core and accessory homologs at least in these two cases.

aTf1 and cTf1 are transcriptional activators

Since expression of *aTF1* and *cTF1* from the *FEM1* promoter was shown to potently induce the *SIX1* promoter, and the *SIX1* promoter contains several aTf1 DNA binding sites, we proceeded to test the results of the DNA binding array in an independent system. For this we used the *aTF1* homolog (FOXG_17458) used for overexpression and *cTF1* in the *in vivo* transcriptional activation assay described above for Sge1. In this case, a short fragment was cloned from the promoter of the *SIX1* effector gene. This sequence includes two Tf1-DNA binding motifs that overlap with the previously identified motif AACTGCCGA and are separated by 17 basepairs. We also constructed a version with mutated Tf1 DNA-binding sites (two basepair substitutions; Fig 5, lower panel). Both aTf1 and cTf1 are able to activate transcription from the construct with the wild type *SIX1* promoter fragment, but not the mutated fragment (Fig 5). This assay was performed in the yeast strain lacking *SGE1* homologs (YEL007 or Mit1 and YHR177), showing that DNA binding and transcriptional activation by aTf1 and cTf1 does not require (a homolog of) Sge1.

Transcriptome analysis of *aTF1*, *cTF1* or *SGE1* overexpressors

We found that only *SGE1*, *aTF1* and its core homolog *cTF1* bind to motifs enriched in the promoters of plant-induced genes and genes on accessory chromosomes. Also, expression of any of these three transcription factor genes from the *FEM1* promoter induces effector *(SIX1)* gene expression. Of both *SGE1* and *cTF1* a role in pathogenicity has been established previously: the Fol *Δsge1* mutant is no longer pathogenic and a *Δctf1 (Δftf2)* mutant is reduced in pathogenicity [26,45]. Expression of all three transcription factor genes is increased upon infection of tomato plants [26,35,36]. To investigate which genes are regulated by these transcription factors, we compared the transcriptomes of *SGE1*, *aTF1* and c*TF1* overexpressors with the transcriptome of the background strain. Overexpressors were preferred over gene deletion strains in this setup because effector genes are only expressed during colonization of the plant, and the plant signal that induces this up-regulation is unknown. Since the deletion mutants are not pathogenic or reduced in pathogenicity, expression cannot reliably be studied *in planta* with gene deletion strains either. Given that expression levels of *SGE1*, *aTF1* and *cTF1* increase during infection, we assume that strains overexpressing either of these transcription factor genes partially mimic the *in planta* state.

RNA was isolated from two-day old cultures growing in minimal medium. For each transcription factor two independent overexpressors were used, and for each strain three independent biological replicates were sampled. Per sample between 2.8*10^7 and 4.8*10^7 reads were paired-end sequenced (Illumina). Reads were mapped to the reference genome (Fol4287) and differentially expressed genes were called for each pairwise comparison with the background

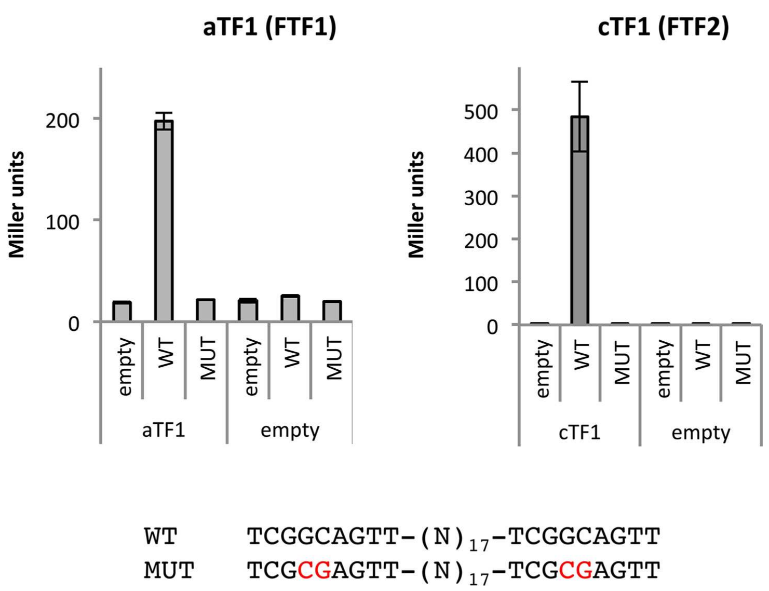

Fig 5. aTf1 and cTf1 (Ftf1 and Ftf2) are transcriptional activators. aTf1 and cTf1 can transcriptionally activate an UAS-less *CYC1* promoter via the presence of two Tf1 DNA binding sites. Activation is lost when the Tf1 DNA binding site is absent or mutated (lower panel). Activation assays were performed in duplicate, error bars represent the standard deviation.

doi:10.1371/journal.pgen.1006401.g005

strain, using DEseq software [52]. The different pairs yielded between 396 and 691 differentially expressed genes (S6A Fig).

To compare the fold induction of expression of the overexpressed transcription factor genes to their induction during infection, we isolated RNA from Fol-infected tomato plants and from axenic cultures. Both transcriptomes of three biological replicates were sequenced and sequencing reads were mapped to the same annotation of the reference genome as above, using the same parameters. As can be seen in Fig 6, expression of each of the three transcription factor genes is increased in the respective overexpressors as well as during infection. The increase in expression is significant ($p<0.05$) for all three transcription factors in their respective overexpressors, but during infection only for *aTF1*. Q-RT-PCR confirmed a significant increase of *aTF1* expression in *aTF1* overexpressors and during infection, but could not confirm significant differences for *cTF1* and *SGE1* transcript abundance (S7 Fig).

Our inability to measure a (significant) increase of *cTF1* and *SGE1* transcripts above their basal expression level by Q-RT PCR was unexpected. *SIX1* (*GFP*) induction in the independent *cTF1* and *SGE1* overexpressors may be caused by an only modest increase in transcript levels, as suggested by our RNAseq analysis. For the *aTF1* overexpressors, expression of both *SIX1* (*GFP*) and another effector gene (*SIX3*) does correlate well with *aTF1* levels (S8 Fig). Also, only in transformants expressing *aTF1*, *cTF1* or *SGE1* from the *FEM1* promoter did we ever observe high GFP levels in the *Psix1*:*GFP* strain (Fig 4, S5A Fig). Still, we wished to exclude the possibility that the transcriptome changes in the *aTF1*, *cTF1* and *SGE1* overexpressors could be due to spontaneous changes unrelated to the transcription factor. We therefore clustered gene expression data of significantly differentially expressed genes for all six overexpressors (S8 Fig). Both *aTF1* and both *cTF1* overexpressors cluster together, showing that genome wide changes in gene expression in these strains are related to the overexpressed transcription factor. The *SGE1* overexpressors, however, do not form a single clade, so we could not link the transcriptional changes in these strains to *SGE1* with this approach.

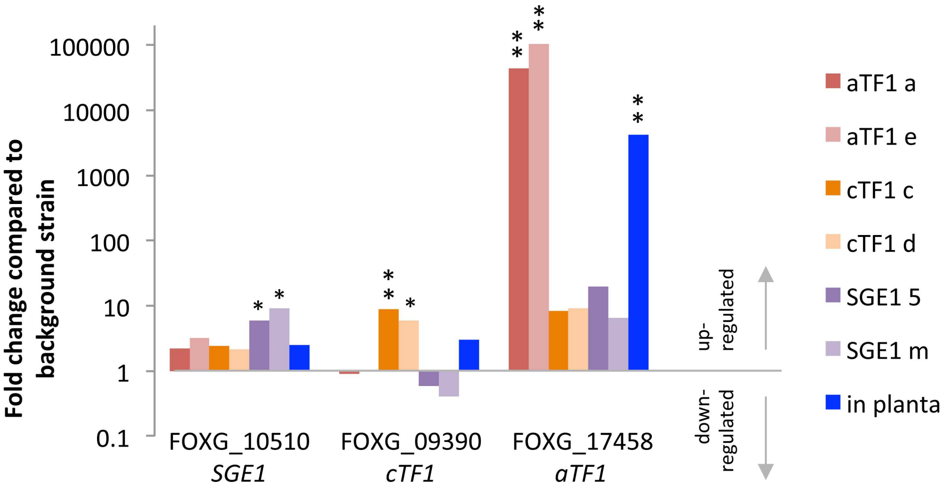

Fig 6. Fold induction of *aTF1* (*FTF1*), *cTF1* (*FTF2*) and *SGE1* expression in overexpressors and during infection. Fold induction of *SGE1*, *aTF1* and *cTF1* transcript abundance during Fol4287-infection (compared to axenic growth of Fol4287) and in their respective overexpressors compared to the background strain (Fol007 Psix1GFP). Red hued bars: *aTF1* overexpressors, orange hued bars: *cTF1* overexpressors, purple hued bars: *SGE1* overexpressors. Blue bars: comparison between Fol4287 *in planta* and Fol4287 axenic cultures. The wild type strains Fol4287 and Fol007 are very similar [7,50]. One star (*): p<0.05, two stars (**): p<0.01. Note that to calculate fold change, all values of zero were replaced by 0.1 (half of the lowest value in the dataset). This includes the *aTF1* levels in *cTF1* and *SGE1* overexpressors.

doi:10.1371/journal.pgen.1006401.g006

Upstream regions of genes up-regulated in strains overexpressing *aTF1*, *cTF1* or *SGE1* are enriched for the respective DNA binding sites

To further test the connection between the selected transcription factors and the changes in the transcriptome, we determined whether the DNA binding site of each transcription factor is enriched in the upstream regions of the genes that are differentially expressed in the *aTF1*, *cTF1* or *SGE1* overexpressors. For this we only considered those genes that were significantly up- or down-regulated in both overexpressors of each transcription factor to be consistent transcription factor-specific effects and this selection was used in all further analysis. Under these criteria, each transcription factor up-regulates 65 to 116 genes, and down-regulates 273 to 347 genes, when overexpressed (S6B Fig). We have analysed single occurrence of the Sge1 DNA binding site, triple occurrence of the (relatively short) Tf1 DNA binding site, and single occurrence of the longer motif found in effector gene promoters (that overlaps with the Tf1 DNA binding site) for enrichment in 1kb promoter regions or coding regions (Fig 7, S4C &S4D Fig, S1 **Data: tab 'promoter DBS' and 'ORF DBS'**). Both the short and the long Tf1 DNA binding sites are significantly and specifically enriched in the promoters of genes up-regulated in both the *aTF1* and *cTF1* overexpressors, but not in the *SGE1* overexpressor. The Sge1 DNA binding site, on the other hand, is specifically enriched in the promoters of *SGE1*- but also *aTF1*- and *cTF1*-up-regulated genes. This links the presence of DNA binding sites to changes in gene expression for all three transcription factors. For none of the down-regulated genes a significant enrichment of DNA binding sites was found, neither for down-regulated genes on the pathogenicity chromosome nor for down-regulated genes on the core chromosomes. This is consistent with the observation that all three transcription factors act as transcriptional activators.

Finally, the enrichment of the Tf1 and Sge1 DNA binding site among three other groups of genes was tested (Fig 7). The three groups are: i) genes coding for small secreted proteins; ii) genes with a (partial) miniature impala (MIMP), a non-autonomous transposable element, in

Number of genes with ≥1-3 TF DBS in the 1000 bp upstream the transcriptional startsite

category		all genes		aTF1 ≥3 DBS	effector motif ≥1 DBS	SGE1 ≥1 DBS
	all genes	20392	fwd	3528	136	2749
			rev	3491	141	2770
UP regulated	in planta	896	fwd	188	**20**	**178**
			rev	**214**	14	**179**
	in aTF1 OE	103	fwd	31	**6**	**34**
			rev	**35**	**6**	**38**
	in cTF1 OE	65	fwd	**26**	**5**	**20**
			rev	**27**	**5**	**21**
	in SGE1 OE	117	fwd	30	5	**34**
			rev	31	2	**39**
DOWN regulated	in planta	140	fwd	27	0	19
			rev	30	0	16
	in aTF1 OE	273	fwd	64	1	25
			rev	43	1	27
	in cTF1 OE	347	fwd	77	1	42
			rev	59	5	37
	in SGE1 OE	278	fwd	68	1	29
			rev	43	0	32
	small secreted proteins	876	fwd	160	9	**155**
			rev	145	**20**	**166**
	MIMP in the promoter	73	fwd	22	1	**22**
			rev	24	1	**24**
	found in xylem sap	55	fwd	14	2	**22**
			rev	18	2	13

Fig 7. Upstream regions of genes up-regulated in strains overexpressing *aTF1* (*FTF1*), *cTF1* (*FTF2*) or *SGE1* are enriched for the respective DNA binding sites. The number of genes with ≥1 or ≥3 transcription factor DNA binding sites (TF DBS) in the promoter region, for the complete genome, or for a subset of genes. DNA binding sites are i) the Tf1 DNA binding site (≥3 occurrences), ii) the motif found in the promoters of effector genes, overlapping with the Tf1 DNA binding site (≥1 occurrences) and iii) the Sge1 DNA binding site (≥1 occurrences). Subsets of genes are: up- or down-regulated during infection (*in planta* UP and *in planta* DOWN), up- or down-regulated in the *aTF1*, *cTF1* or *SGE1* overexpressor (*aTF1* UP, c*TF1* UP, *SGE1* UP, *aTF1* DOWN, *cTF1* DOWN and *SGE1* DOWN), genes that code for small secreted proteins, genes with a MIMP (miniature impala) in the promoter and genes that encode proteins that have been found in xylem sap of infected plants [34]. Boxes indicate a significant enrichment (P <0.01 after Bonferroni correction).

doi:10.1371/journal.pgen.1006401.g007

the upstream region (up to 2 kb from the ATG—all Fol effector genes are associated with a MIMP, but also other, mainly accessory genes, of which around half is also up-regulated during infection (S6B Fig, [34])); iii) genes of which the protein has been detected in xylem sap of infected plants [34]. The Tf1 DNA binding site is not enriched among these categories, except the long effector motif among small secreted proteins. Remarkably, the Sge1 DNA binding site is enriched in all these categories (Fig 7, lower panel), suggesting that Sge1 may target pathogenicity-associated genes more specifically than aTf1 or cTf1.

Expression of *aTF1*, *cTF1* or *SGE1* from the *FEM1* promoter induces expression of many plant-responsive genes on the pathogenicity chromosome

We have shown that the genome-wide transcriptional changes in the *aTF1* and *cTF1* overexpressors are correlated with expression of the respective transcription factor from the *FEM1*

promoter, while the Sge1 binding site is enriched in upstream regions of genes upregulated in the *SGE1* overexpressors and of pathogenicity-associated genes. We now compared the gene sets differentially expressed in the *SGE1*, *aTF1* and *cTF1* overexpressors and during infection (Fig 8A **and** S6B Fig). Strikingly, the majority of the plant-responsive genes on the pathogenicity chromosome is also up-regulated in all three overexpressors, including almost all *SIX* effector genes (indicated in purple text in Fig 8B). The *SIX* genes that are not induced are *SIX13* in all overexpressors and *SIX2* in the *SGE1* overexpressors. On the other accessory regions (chromosome 3, 6, 15 plus small regions on chromosome 1 and 2) the overlap between plant responsive genes and Sge1-, aTf1- or cTf1-responsive genes is much smaller. Here a large group of genes is down-regulated in all transformants but not during infection. Of this group many genes are generally weakly expressed. We noticed that lower expression levels of these genes–as observed in the overexpressors–was also found in wild type strains (S9 Fig). This suggests that the apparent down-regulation of expression from this region may not be due to increased abundance of one of the three transcription factors, but rather to general variability in gene expression in this region between transformants. Another transcription factor-unrelated change in expression was observed for a part of the pathogenicity chromosome. In Fig 8B, the top rows of the enlargement of the pathogenicity chromosome show a group of genes up-regulated in one of the *cTF1* overexpressors and one of the *SGE1* overexpressors. Closer examination showed that these genes are part of a region on supercontig 22 that is generally higher expressed in these two transformants (S10 Fig). Given the regional nature and the lack of correlation to a particular transcription factor, we suspect these differences may be either caused by changes in chromatin state, or by 'spontaneous' duplication of these regions. Spontaneous duplications in accessory regions have been reported previously in *F. oxysporum* [53].

The majority of all plant-induced genes is located on the core chromosomes, but only a small portion of those is differentially regulated in the *aTF1*, *cTF1* or *SGE1* overexpressors. Remarkably, whereas these three transcription factors seem to target the same set of genes on the pathogenicity chromosome, on the core genome the overlap between the genes of which expression is altered by the different transcription factors is much smaller, and each mostly induces a specific set of genes, especially Sge1 (S6A Fig). Still, the genes up-regulated by the transcription factor genes on core chromosomes are enriched for plant-responsive genes (hypergeometric test, P value < 0.05 after Bonferroni correction).

To see which functional categories of genes apart from effector genes are targeted by aTf1, cTf1 and Sge1, a FunCat analysis was performed [54] (S1 Data**: tab 'FungiFun'**). Of the genes up-regulated in *SGE1* overexpressors, genes in the categories heme binding (including genes coding for cytochrome P450 proteins), catalase reactions and electron transport (including ATPases and oxidoreductases) are overrepresented. Among the predicted functions of the genes induced in the *cTF1* overexpressors there is enrichment in secondary metabolism and C-compound and carbohydrate metabolism (including polysaccharide metabolism and protein glycosylation). The set of genes induced by aTf1 is also enriched for genes in secondary metabolism and C-compound and carbohydrate metabolism (including polysaccharide and chitin metabolism as well as pectate lyases).

In the *SGE1* overexpressors a peroxisome biogenesis factor (*PEX11*) is up-regulated. Peroxisome function is required for pathogenicity in Fol [55]. Both aTf1 and cTf1 up-regulate expression of the shorter *aTF1* gene on the pathogenicity chromosome (FOXG_16414), although not to the same level as during infection. Apart from this *aTF1* homolog, expression of only one other transcription factor gene is significantly altered in any of the overexpressors: aTf1 up-regulates FOXG_04965. Strikingly, this transcription factor is required for pathogenicity [45].

The set of down-regulated genes on the pathogenicity chromosome and the core chromosomes was not significantly enriched for certain categories for aTf1 and cTf1 regulated genes. Sge1-repressed genes were enriched for genes in polysaccharide metabolism and amino

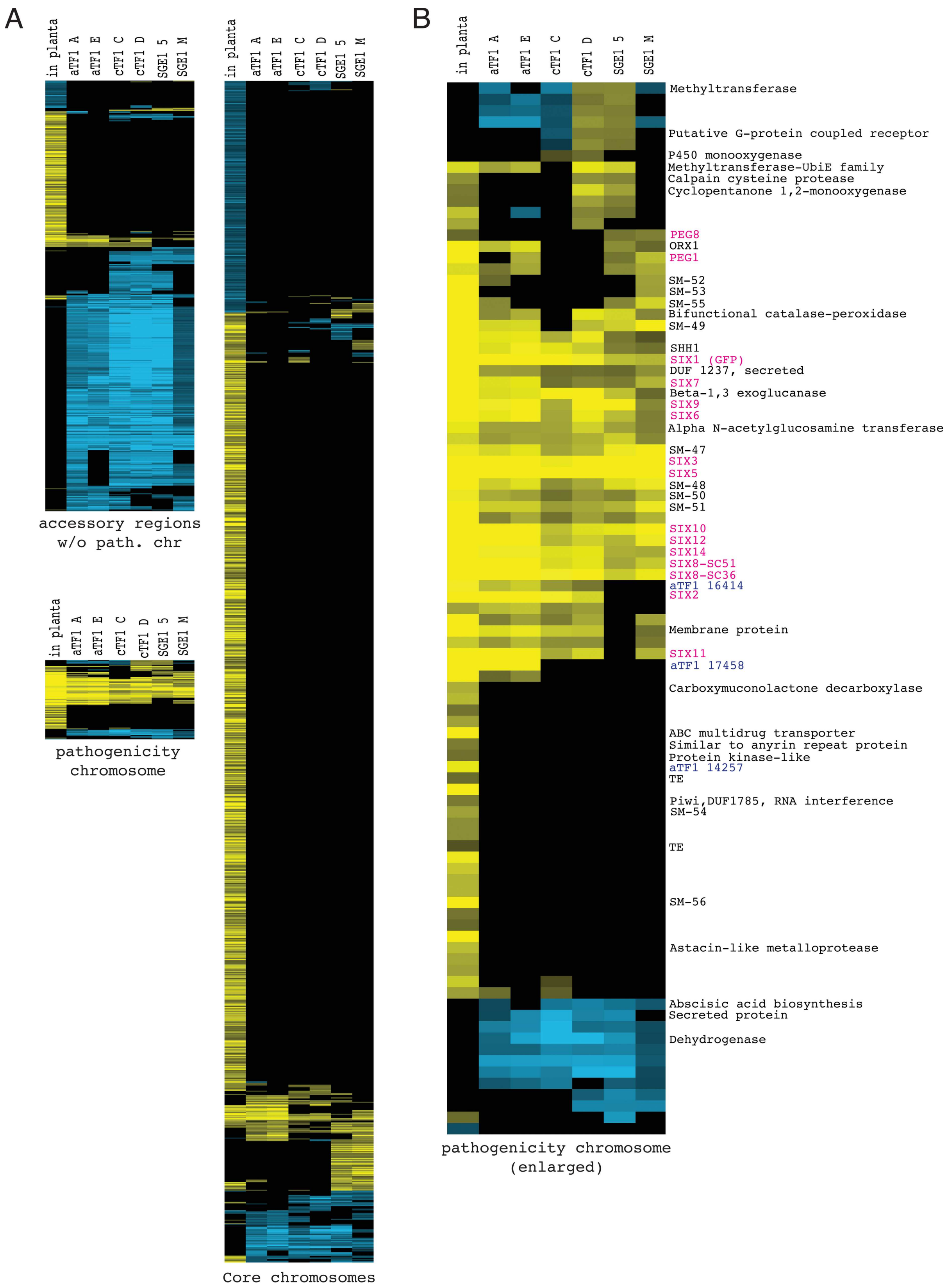
A
in planta
aTF1 A
aTF1 E
cTF1 C
cTF1 D
SGE1 5
SGE1 M
accessory regions w/o path. chr
pathogenicity chromosome
Core chromosomes
B
Methyltransferase
Putative G-protein coupled receptor
P450 monooxygenase
Methyltransferase-UbiE family
Calpain cysteine protease
Cyclopentanone 1,2-monooxygenase
PEG8
ORX1
PEG1
SM-52
SM-53
SM-55
Bifunctional catalase-peroxidase
SM-49
SHH1
SIX1 (GFP)
DUF 1237, secreted
SIX7
Beta-1,3 exoglucanase
SIX9
SIX6
Alpha N-acetylglucosamine transferase
SM-47
SIX3
SIX5
SM-48
SM-50
SM-51
SIX10
SIX12
SIX14
SIX8-SC51
SIX8-SC36
aTF1 16414
SIX2
Membrane protein
SIX11
aTF1 17458
Carboxymuconolactone decarboxylase
ABC multidrug transporter
Similar to anyrin repeat protein
Protein kinase-like
aTF1 14257
TE
Piwi,DUF1785, RNA interference
SM-54
TE
SM-56
Astacin-like metalloprotease
Abscisic acid biosynthesis
Secreted protein
Dehydrogenase
pathogenicity chromosome (enlarged)

Fig 8. Expression of *aTF1* (*FTF1*), *cTF1* (*FTF2*) or *SGE1* from the *FEM1* promoter induces many plant-responsive genes on the pathogenicity chromosome. A) Heatmap of differentially expressed genes during infection or in *aTF1*, *cTF1* or *SGE1* overexpressors for the pathogenicity chromosome, other accessory regions and core genome. Displayed is the log2 fold change for each differentially expressed gene (rows), with yellow indicating up-regulation compared to the control (hues cover the log2 fold range from 0 to 5). Blue indicates down-regulation compared to the control (hues cover the log2 fold range from 0 to -5). The log2 fold change values of genes that are not significantly different from the control were set to zero. Each condition is a separate column, with two independent transformants (thus 2 columns) per transcription factor. The order of the rows reflects clustering of similar expression patterns. **B**) Enlarged heatmap of the pathogenicity chromosome, adorned with gene names: (putative) effector genes are shown in pink, transcription factor genes in blue (aTF1 17458 = FOXG_17458, aTF1 14275 = FOXG_14257, both the longer version of a*TF1*, aTF1 16414 = FOXG_16414, shorter version of *aTF1*), other genes are shown in black. SM-47 to SM-56: putative secondary metabolite gene cluster, FOXG_17447 to FOXG_17456.

doi:10.1371/journal.pgen.1006401.g008

saccharide metabolism. Taken together, all three transcription factors influence effector gene expression and secondary metabolism and target genes predicted to affect both the fungal and the plant cell wall (**S1 Data: tab 'FungiFun'**).

Expression of *aTF1*, *cTF1* or *SGE1* from the *FEM1* promoter does not generally increase transcription of transposons

Although very different transcription factors, aTf1/cTf1 and Sge1 induce expression of a large, overlapping set of genes on the pathogenicity chromosome. Together with the observation that some regions may be prone to activation that is not linked to a specific transcription factor gene, this made us wonder whether the pathogenicity chromosome is transcriptionally activated as a whole, rather than gene by gene. The accessory chromosomes are very rich in transposable elements, and we decided to investigate expression of transposable elements as a proxy for chromosome-wide transcriptional activation. Besides, we were interested to see whether general up-regulation of genes on the pathogenicity chromosome (for instance during infection) could jeopardize genome integrity by induction of transposon activity.

One problem, however, is that in our RNAseq analysis the number of transcripts derived from transposable elements was probably highly underestimated, because: i) many transposons are not annotated, ii) reads of identical transposons are distributed over all copies, obscuring activation of individual copies, and iii) reads mapping to more than ten different genomic locations were excluded. To circumvent this, a fasta file was generated where the sequence of each repetitive element, plus all previously annotated transposable elements [34] is present only once. To this file, all sequence reads from the overexpressors as well as reads from infected plant material were mapped (**S11 Fig and S6D Fig**). We have found no evidence for a general increase of transposable element transcription in the *aTF1*, *cTF1* or *SGE1* overexpressors or during plant infection.

Induction of effector gene expression mediated by *aTF1* overexpression is Sge1 dependent

We have shown that expression of Sge1, aTf1 or cTf1 from the *FEM1* promoter is correlated with induced expression of a large overlapping set of genes, including effector genes. Whereas aTf1 and cTf1 are homologs and have the same DNA binding specificity, Sge1 is a very different transcription factor, with a strongly conserved, fungal specific DNA binding domain. This raises the question what causes this overlap in transcriptional activation. Expression of for example *SIX1* and *SIX3* is induced in *aTF1*, *cTF1* and *SGE1* overexpressors. However, whereas *SIX3* has both Tf1 and Sge1 DNA binding sites present in the promoter, for *SIX1* only Tf1 DNA binding sites are present. To test whether the presence of Sge1 is required for *aTF1* overexpression-mediated induction of *SIX1* and *SIX3*, we deleted *SGE1* in an *aTF1* overexpressing strain. Without the presence of *SGE1*, the expression of the effector genes *SIX1* and *SIX3* was

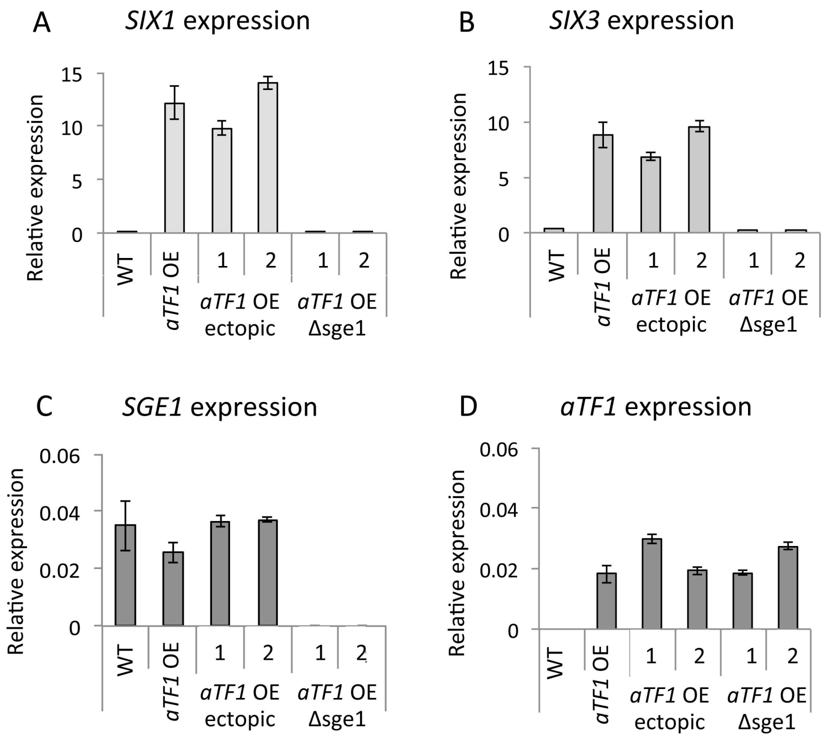

Fig 9. Induction of effector gene expression mediated by *aTF1* (*FTF1*) overexpression is Sge1 dependent. Relative expression as determined by Q-RT-PCR of *SIX1* (**A**), *SIX3* (**B**), *SGE1* (**C**) and *aTF1* (**D**) in WT, *aTF1* overexpressor (aTF1 OE), *aTF1* overexpressor transformed with a *SGE1* deletion construct, integrated ectopically (*aTF1* ectopic, two independent transformants), and *aTF1* overexpressor with *SGE1* deleted (aTF1 OE Δsge1, two independent transformants). Relative expression is calculated as gene expression/expression of *EF1α*. Error bars indicate standard deviation.

doi:10.1371/journal.pgen.1006401.g009

reduced to low (wild type) levels, while overexpression of *aTF1* itself remained unchanged (Fig 9). This shows that Sge1 is required for *aTF1* overexpression-mediated activation of effector gene expression, but not necessarily via an Sge1 DNA binding site. Also, deletion of *SGE1* resulted in loss of pathogenicity in both WT and in *aTF1* overexpressing strains (S12 Fig).

In summary, we have demonstrated that some pathogenicity chromosome-encoded transcription factors have regulating potential on the accessory chromosomes themselves, and that *aTF1* and the core-encoded *cTF1* and *SGE1* can induce effector gene expression and expression of other plant-responsive genes upon constitutive expression, possibly via direct binding of the transcription factors to these promoters and subsequent transcriptional activation.

Discussion

This study aimed to gain insight in the transcriptional connections between core chromosomes and the accessory 'pathogenicity' chromosome in Fol, especially with respect to effector gene expression. We show that expression of the transcription factor genes *aTF1* (*FTF1*, located on the pathogenicity chromosome), *cTF1* (*FTF2*) or *SGE1* (both located on the core) from the constitutive *FEM1* promoter induces expression of many plant responsive genes located on the pathogenicity chromosome, particularly effector genes. We conclude that the pathogenicity chromosome is transcriptionally partially self-regulating, but not isolated from the core genome.

How do Sge1 and aTf1 work together?

The large overlap in the sets of target genes of aTf1/cTf1 and Sge1 on the pathogenicity chromosome suggests these transcription factors somehow work together. This is substantiated by the observation that *aTF1* overexpression-mediated induction of effector gene expression requires Sge1. Overexpression of *SGE1* orthologs in *F. graminearum*, *F. verticillioides* and *F. fujikuroi* induces the expression of secondary metabolite genes [23–26]. Apart from *SGE1*, expression of secondary metabolite gene clusters depends on specialized transcription factors often physically associated with the cluster [56]. Reminiscent of this, *SIX* effector genes sometimes occur in mini clusters of two or three *SIX* genes, accompanied by a *aTF1* homolog. Genes that are located between or physically close to clustered *SIX* genes (*ORX1*, *SHH1*) are co-induced, both during infection and in the overexpressors.

Transcriptional regulation of secondary metabolite clusters partially takes place on the chromatin level [57,58]. Some observations hint at chromatin-mediated regulation of expression of genes on the pathogenicity chromosome of Fol as well. First, there is the transcription factor unrelated up-regulation of parts of SC22 on the pathogenicity chromosome (in one *cTF1* and one *SGE1* overexpressor, this study). Second, the *BLE* resistance gene behind the constitutive *GPD* promoter located near the *SIX1* locus is co-up-regulated in some of the overexpressors (**S1 Data, tabs: 'total mapped reads' and 'RPKM'**. Finally, physical interactors of the *Saccharomyces cerevisiae* Sge1 ortholog Mit1 include histones and histone acetyl transferase complexes and are enriched for the GO terms chromosome organization, chromatin assembly or disassembly and chromatin organization (yeastgenome.org). This raises the question whether the large overlap of genes on the pathogenicity chromosome affected by expression of a*TF1/cTF1* and *SGE1* from the *FEM1* promoter may be caused by a shared influence on chromatin structure. It will be interesting to see if facultative heterochromatin functions as a layer of effector gene expression regulation also in *F. oxysporum*.

Another explanation for the overlap between the *aTF1*, *cTF1* and *SGE1* targets on the pathogenicity chromosome is that Sge1 induces effector gene expression via *aTF1*/*cTF1* or *vice versa*. Despite the fact that *SGE1* has potential aTf1 binding sites in its promoter and one *aTF1* (FOXG_17458) has potential Sge1 DNA binding sites in its promoter, neither of the genes is constitutively up-regulated by overexpression of the other. Moreover, Sge1 is still required for effector gene expression when *aTF1* is overexpressed. Also, there are clear differences between the different overexpressors, for example *SIX11* and *SIX2* are induced in *aTF1*- and *cTF1*-overexpressors, but not in *SGE1*-overexpressors. *SIX11* and *SIX2* indeed have no Sge1 DNA binding site in their promoter. *SGE1* is, however, required for the induced expression of *SIX2* upon exposure of Fol to tomato cell cultures [26].

Alternatively, Sge1 and aTf1/cTf1 may be simultaneously required at promoters for transcription activation. Sge1 and cTf1 both have basal (non-induced) expression levels, and overexpression of either might cause recruitment of the other (perhaps by direct interaction or through chromatin modification) leading to transcription activation. However, we have shown that Sge1 as well as Tf1 can bind DNA and activate transcription independent of each other. A similar situation has been described for the Sge1 homolog Ryp1 and two velvet-like transcription factors in *H. capsulatum* [59]. A subset of the genes induced by overexpression of Ryp1 is also induced by overexpression of either of the two velvet-like transcription factors. These shared target genes have DNA binding sites for each transcription factor in their promoter, and the three transcription factors physically interact.

To unravel the question of Sge1/Tf1 co-operation, it will be necessary to determine physical interactions, both between them and with promoters, under basal conditions and upon overexpression, in combination with analysis of chromatin structure at the same promoters.

Are accessory transcription factor homologs redundant?

Next to (nearly) identical DNA binding sites, several observations support an overlap in function between homologous transcription factors encoded on accessory and core genomes. *aTF7*- and *cTF7*-overexpressing strains show a similar growth retardation and reduction of virulence, and aTf1 and cTf1 both are potent inducers of effector gene expression. In addition, a deletion mutant of *cTF1* is less virulent, but can still colonize the plant [45,60]. The partial loss of virulence could be a result of redundancy between *cTF1* and *aTF1*. Deletion of either of two of the shorter *aTF1* homologs (FOXG_16414 and FOXG_17123) does not affect pathogenicity [45], but recently silencing affecting both *aTF1* and *cTF1* was shown to reduce virulence in both f. sp. *lycopersici* and f. sp. *phaseoli* [60]. Also host-induced gene silencing of *aTF1*, potentially silencing the entire gene family, has been reported in *F. oxysporum* f. sp. *cubense* on banana, which led to complete absence of disease symptoms [61].

If there indeed is such a large functional overlap between core and accessory homologs, then why does Fol contain transcription factor genes on the pathogenicity chromosome at all? And why—in some cases–even multiple homologs? One possibility is that the presence of the transcription factor (and other) genes on accessory chromosomes did not result from selective pressure on a particular functional advantage but is simply provoked by certain features of accessory chromosomes, such as high transposable element density or chromatin structure. A different, but not mutually exclusive, view is a selective advantage of gene duplications because of a dosage effect. In *F. oxysporum* f. sp. *phaseoli*, a correlation between virulence and the number of *aTF1* homologs has been reported [35].

It should be noted, however, that although very similar, functions of core and accessory-encoded homologous transcription factors may not be completely redundant. For example, overexpression of *aTF1* (FOXG_17458) induces expression of a transcription factor required for pathogenicity, whereas overexpression of *cTF1* does not. Also, the short *aTF1* (FOXG_17084) homolog can bind DNA with the same specificity, but is far less efficient in inducing *SIX1* expression. At present, it cannot be excluded that the short homolog might even function as a negative regulator.

Is the transcriptional regulation of the pathogenicity chromosome relevant for horizontal transfer?

It is currently unclear over how large a phylogenetic distance the pathogenicity chromosome can be transferred. Up to now, transfer as only been experimentally demonstrated within the *F. oxysporum* species complex [7]. In such a case the *cTF1* and *SGE1* homolog in the recipient strain will be nearly identical to the chromosome donor strain, only accessory-encoded aTf1 homologs on regions other than the pathogenicity chromosome will be absent or different in the recipient. Interesting in this respect is a previous observation: when the pathogenicity chromosome is transferred to the non-pathogenic strain Fo-47, this strain gains pathogenicity, but is not very aggressive. Virulence is higher in those strains that gained not only the pathogenicity chromosome, but also a second small chromosome, corresponding to the duplicated region of chromosome 3/6 in the reference strain Fol4287 [7]. This region contains few if any effector genes, but many transcription factor genes, including several homologs of *aTF1*, *aTF4*, *aTF6*, *aTF7*, *aTF8 (EBR2)* and *aTF9*.

Genome analyses also suggest at least one ancient transfer event from a *Fusarium* species at a phylogenetic distance from Fol somewhere between *F. verticillioides* and *F. graminearum* [7]. If such a transfer were to occur again, the *cTF1* homolog in the recipient would still be very similar to the c*TF1* homolog in the donor strain, at least more similar than *cTF1* is to *aTF1*. No additional accessory *aTF1* homologs would be present in the recipient. We speculate that the

transcription factors on accessory chromosomes do contribute to virulence, but many of their roles could be also partially fulfilled by their core-encoded homologs within the genus *Fusarium*.

For *SGE1* homologs, significant functional differences have been reported between closely related species, like different *Fusarium spp*, but also between *C. albicans* and *S. cerevisiae* [23–25,33]. As described above, the DNA binding domain at the N-terminus is very conserved, and so is–as far as it has been tested–the DNA binding site [33], whereas the C-terminal domain is very variable [24,25]. For the yeasts *C. albicans* and *S. cerevisiae*, differences in target genes are caused by differences in the presence or absence of cis-acting promoter elements [33]. For *F. graminearum* and Fol, however, differences in target genes are caused by differences in the C-terminal domain, and transcomplementation can partially restore pathogenicity only in some cases [24]. Also in *F. fujikuroi* and *F. verticillioides* overexpression of their respective *SGE1* homologs regulates a different set of genes [23,25].

Since the differences between the Sge1 homologs between different *Fusarium* species are so substantial that Sge1-mediated transcription regulation is extensively rewired, this may be a limiting factor to expression of effector genes from the Fol pathogenicity chromosome in another *Fusarium* species. Interestingly, transcomplementation of the Δ*sge1* mutant of Fol with *CfWOR1* from *C. fulvum* resulted in restoration of effector gene expression but not in restoration of pathogenicity [28].

SGE1 overexpressors and the Δ*sge1* mutant enable identification of different potential target genes.

Previously, Jonkers and co-workers have looked at differentially expressed genes in a Fol *SGE1* deletion mutant compared to WT using a microarray [24]. This set (1213 genes) is very different from the differentially expressed genes in the *SGE1* overexpressors that we found (168 genes); only 15 genes are present in both sets. This shows that the different approaches are complementary for identification of target genes. The genes differentially expressed between WT and deletion strain are predominantly present on the core genome. Also, the Sge1 DNA binding site is not significantly enriched among up- or down-regulated genes in the deletion mutant (104 [out of 394] down-regulated genes with a Sge1 DNA binding site and 171 [out of 820] up-regulated genes, on a total of 4870 [out of 20935] genes with a Sge1 DNA binding site), in contrast to the *SGE1* overexpressor. This may be because several transcription factor genes are differentially expressed in the deletion mutant, potentially regulating secondary targets. Also, the comparison of WT and *SGE1* deletion mutant was of necessity conducted under axenic growth conditions which excludes finding targets that are not or very weakly expressed under those conditions, including most genes on the accessory genome.

What is the role of the other transcription factors encoded on the pathogenicity chromosome?

We have shown that aTf1 can activate effector gene expression. However, many more transcription factors are encoded on the pathogenicity chromosome. It is tempting to speculate that these transcription factors may control expression of some of the other plant-responsive genes. The core-encoded homologs of some of the other accessory transcription factors have been implicated in pathogenicity. *cTF8 (EBR1)* is required for full pathogenicity of Fol and *F. graminearum*, and orthologs of *cTF4* and *cTF9* are required for pathogenicity of *F. graminearum* (FGSG_10057, FGSG_10517 and FGSG_06651 respectively) [37,62]. Of these three transcription factors, we have only obtained a DNA binding site for cTf4, which is, like the DNA binding sites of aTf2, aTf5 and aTf7, not enriched among genes up- or down-regulated

during infection. It is of course possible that there is not always a significant correlation between the presence of the DNA binding site and changes in expression during infection. This may occur when loss of pathogenicity is an indirect effect (via a second, downstream transcription factor), when only a particular combination of transcription factors regulates plant responsive genes, or when too many apparent binding sites are not functional, precluding detection of a significant association between binding site and gene regulation.

Of two transcription factors (cTf4 and aTf5), DNA binding sites are enriched on the accessory chromosomes, suggesting they may also act on genes of the accessory chromosomes. In *F. graminearum*, global gene regulatory network modelling revealed that species-specific genes are most often controlled by species-specific regulators, whereas genes conserved between species are controlled by conserved regulators [63]. Possibly, such a compartmentalized network structure of gene regulation may also apply to *F. oxysporum*.

Other putative functions of the transcription factors encoded on the pathogenicity chromosome could be downregulation of effector gene expression and returning to or maintaining a repressed, saprotrophic state. They may also be promoting horizontal transfer. It would be interesting to see if the pathogenicity chromosome can be lost, and if so, in which ways this may affect the phenotype of Fol and expression of core genes.

Materials and Methods

Cloning

In order to determine the DNA binding sites of the different transcription factors, the ORFs were cloned from cDNA. For this, RNA was isolated from axenic cultures and from susceptible infected tomato plants, between 1–2 weeks after inoculation. cDNA was generated as described below. For some transcription factors alternative start codons were tried, and the longest obtained PCR product was selected for cloning. For many transcription factors, 35 PCR cycles was insufficient to amplify clonable amounts of DNA, therefore, a re-amplification was done. Primers were designed in such a way that the subsequent product could be cloned with AscI, BamHI or SbfI, in frame with a N-terminal GST-tag in an *Escherichia coli* T7 expression vector pTH6838. The cloned ORF was checked by sequencing.

To express the transcription factor genes in Fol, the binary vector pRW2h was modified, yielding a plasmid with a right border (facilitating *Agrobacterium tumefaciens* mediated transformation), the *FEM1* promoter, a multiple cloning site including XbaI, AscI, StuI, SbfI, BglII and ApaI, followed by the *SIX1* terminator, the *HPH* resistance cassette and the left border. The transcription factor ORF was then cut out of pTH6838 with the same enzymes used to clone it in, and cloned into pRW2h_Pfem_MCS_Tsix1, again with the same enzymes. For those transcription factors of which the gDNA ORF was cloned, a PCR was done on Fol007 gDNA, isolated as described in [64] using the same primers initially designed for cloning of the cDNA. The PCR product was digested and cloned directly into pRW2h_Pfem_MCS_Tsix1. The cloned transcription factor ORFs were checked by sequencing.

Fol transformation

Fol was transformed via *Agrobacterium* mediated transformation, as described previously [65]. Transformants were monospored by pipetting 10–20 μl of sterile water on the emerging colony, and spreading this on a fresh PDA plate supplemented with cefotaxime and Hygromycine. After two days of growth at 25 degree Celsius, single colonies were picked and transferred to fresh plates. From these plates glycerol stocks were made and these are the transformants we worked with. Transformants were only selected on antibiotic resistance, and should contain one (or in rare cases more than one) random ectopic insertion of the T-DNA construct [26].

Microconidia production, harvest of mycelium, harvest of infected plants

Cultures for RNA isolation were grown as described below, except for experiments described in S8A Fig and Fig 9. These RNA isolations were done as described under 'Deletion of *SGE1* in *aTF1* overexpressor background' later in this section. For all other cases a small cube with mycelium from a PDA plate was used to inoculate a preculture of 50 ml liquid minimal medium (1%KNO_3, 3% sucrose 0.17% YNB w/o amino acids or NH_4) and grown for 3 to 5 days at 25°C, shaking 150–175 rpm. From this preculture microconidia were isolated by filtering the culture over miracloth and pelleting the microconidia in the filtrate at 2000 rpm for 10–15 min.

To harvest mycelium for RNAseq analysis of transcription factor overexpressors or for quantification of transcription factor transcripts by Q-RT-PCR, microconidia were suspended in a small volume of minimal medium, counted, and used to inoculate 100 ml liquid minimal medium with 2.5 * 10^8 microconidia. This culture was grown for 2 days at 25°C, shaking 150–175 rpm before mycelium was harvested by filtering the culture over a double layer of miracloth. The mycelium in the filter was washed once with 50–100 ml sterile water, scraped from the miracloth and snap frozen in liquid nitrogen. Of each condition, three independent biological replicates were sampled.

To harvest material for RNAseq analysis of infected plants, Fol4287-infected tomato roots were harvested nine days post inoculation. Infections were performed as described below (for the bioassays). Fol4287 mycelium from axenic cultures was harvested from five day old cultures inoculated from plate in 100 ml liquid minimal medium (1%KNO_3, 3% sucrose 0.17% YNB w/o amino acids or NH_4), 25°C, 150–175 rpm.

RNA isolation, cDNA synthesis & RNAseq

RNA was isolated as described earlier, using a Trizol extraction on mycelium ground in liquid nitrogen, followed by DNase treatment and purification over RNeasy RNA purification columns, according to the instructions of the manufacturer (Qiagen).

Synthesis of cDNA was performed using 1 μg of RNA, poly dT primers, Promega RNasin (ribonuclease inhibitor) and Gibco Superscript II RNase H− Reverse transcriptase, according to instructions of Gibco.

Of 2 μg of total RNA of each biological replicate, polyadenylated RNA was amplified and ligated to adapters to make a library suitable for multiplex illumina paired-end sequencing. Each sample was barcoded and sequenced in 8 different lanes. After de-multiplexing, total reads of the different lanes were combined. This rendered one file of reads per biological replicate.

Quantitative RT-PCR

Quantitative PCR was performed with a model 7500 Real Time PCR system (Applied Biosystems) and Solis BioDyne 5x HOT FIREPol Eva Green qPCR Mix Plus (ROX). Primers used for Q-RT-PCR where designed to amplify fragments of approximately 100 bp and tested for primer efficiency and melting curve (**S1 Data**). 1 μL of cDNA was used per sample, two technical replicates were performed for each sample. Transcription elongation factor 1α (EF1α) gene expression was used as a reference, and RNA that was not transcribed into cDNA as a gDNA contamination control. The following formula was used to calculate the amount of DNA: [DNA] = (1/E^Ct_sample) -(1/E^Ct_control), with E = primer efficiency, Ct_sample = Ct value of the test sample, using WT or TF overexpressor cDNA as a template and Ct_control = Ct value of no cDNA control sample (to check for gDNA contamination), with the same primer pair. The comparison with *EF1α* was made as follows: DNA_TF/DNA_EF1α. Standard deviations of the two

technical replicates per sample were calculated with the following formula: Standard deviation = $\sqrt{}$((stdev DNA_TF /average DNA_TF)^2 + (stdev DNA_EF1α /average DNA_EF1α)^2))* (DNA_TF /DNA_EF1 α).

Whole genome read mapping

The Illumina reads (125 bp paired end, insert size around 200–500 bp) were mapped to the annotated genome of Fol4287 (Fusarium Comparative Sequencing Project, Broad Institute of Harvard and MIT (http://www.broadinstitute.org/), annotation 3) using CLC Genomics Workbench version 6.5.1 module (CLC bio, Aarhus, Denmark). Reads were imported as: illumina (pipeline 1.8 and later), paired-end reads, insert size 100–600 bp, remove failed reads. Imported reads were trimmed to remove any remaining adapter sequences or low quality reads. Quality scores and ambiguous nucleotides were trimmed with standard settings (limit 0.05, ambiguities 2). Adapters were trimmed by checking for the presence of the Truseq Universal adapter (minus strand) and the presence of the Truseq index adapter (plus strand) with the following parameters: mismatch = 2, gapcost = 3, cutoff ns, cutoff at end 6, action: remove adapter.

The following gene models were manually added to the annotation:

Supercontig_2.36 135864–136180 minus strand FOXG_SIX14; Supercontig_2.51 62412–62758 minus strand FOXG_SIX12; Supercontig_2.51 65216–65878 plus strand FOXG_SIX7; Supercontig_2.22 806692–807024 minus strand FOXG_SIX11; Supercontig_2.22 44647–44970 minus strand FOXG_PEG4 (Putative Effector Gene 4); Supercontig_2.36 468654–469317 minus strand FOXG_SIX8-36; Supercontig_2.51 6999–7662 minus strand FOXG_SIX8-51.

Reads were mapped to the annotated genome with parameters: Organism type = eukaryote. Exon discovery according to standard settings: relative expression level = min 0.2, min reads = 10, min length = 50 bp. Additional downstream bases = 0. Additional upstream bases = 0. Minimum length fraction = 0.9. Minimum similarity fraction = 0.95. Minimum number of reads = 10. Map only intact pairs, count paired reads as one. Unspecific match limit = 10. Expression value = Total number of mapped reads and reads per kb per million mapped reads (RPKM).

Whole genome differentially expressed gene calling

Differentially expressed genes were called for pairwise comparisons of the total number of mapped reads (three replicates), using the Bioconductor DEseq software [52]. After normalization for library size (estimateSizeFactors) and variance estimation (estimateDispersion) with parameters "method = blind, sharingMode = fit-only", Nbinominal testing and the Benjamin-Hochberg multiple testing adjustment procedure were used [52]. For the transcription factor gene overexpressing transformants (all compared pairwise to the Fol007 Psix1GFP samples), all genes that had an adjusted p value <0.1 for both overexpressors were considered significant. For the samples from infected plants (compared to the Fol 4287 control samples) all genes with an adjusted p value < 0.05 were considered significant. The output file gives the mean normalized mapped reads per sample (mean of the three replicates), pvalue and adjusted pvalue.

Analysis of differentially expressed genes

For all pairwise comparisons the normalized total mapped reads were collected, and every count of zero reads was replaced by 0.1 (0.1 is roughly half of the lowest number of normalized total mapped reads in all comparisons, this allows calculation of -an approximate- fold change for each gene). Fold change and log2 fold change were calculated. To visualize expression differences in a heatmap, all genes considered differentially expressed in one of the comparisons

were listed and the log2 fold change for each condition was listed in seven subsequent columns. In this list, each value not reaching the significance threshold was replaced by '0' (indicating no fold change). This list was separated into three lists based on subgenome and each of these lists were clustered on gene and condition in Gene Cluster 3.0, uncentered correlation, average linkage. Results were visualized in Java Treeview.

Next to this, the differentially expressed genes were subdivided in lists of up-regulated and down-regulated genes. These lists were used to count the contributions of different subgroups to each category (for example: number of *aTF1* up-regulated genes that is located on the pathogenicity chromosome). Hypergeometrical distribution tests were used to determine significant enrichments or depletions among different categories. The adjusted p value was reached by multiplying the p value with the total number of tests performed.

To determine which genes have a MIMP in their promoter, two kb upstream the ATG of each gene was searched for the presence of complete (both inverted repeats present) or partial MIMPs. Any missing *SIX* gene, of which a MIMP had been demonstrated in the promoter earlier [34], was manually added to the list.

Read mapping and differentially expressed gene calling for transposable elements

The Illumina reads (125 bp paired end, insert size around 200–500 bp) were mapped to the fasta file with all repetitive/transposable elements as described above, except the following parameters: Organism type = prokaryote, unspecific match limit = 30. Reads were normalized as the number of reads per 20*10^6 uniquely mapped reads to the total genome for that particular sample.

For the more detailed analysis the same pairwise comparisons were made as described above. Every count of zero reads was replaced by 0.1, the data was log10 transformed and a T-test was performed on the log10 transformed normalized total mapped reads. All sequences that compared differently ($p<0.05$) were counted.

Bioassay & growth assay

Bioassays were performed as described earlier [66]. Briefly, tomato seedlings of 10 to 11 days were trimmed at the main root and dipped in a Fol microconidia suspension of 0.5*10^7 microconidia/ml for at least 1 minute. The seedlings were potted in soil in individual pots and grown in the greenhouse at 25°C for three weeks. At the time of harvest, the above ground parts were cut off at the cotelydons and scored for fresh weight and disease index. The disease index ranges from 0 (no symptoms), 1 (thickening of hypocotyl, formation of lateral roots), 2 (one brown vessel), 3 (up to ¾ of the vessels show browning, asymmetric development) to 4 (all vessels brown, severe growth retardation, death).

Growth assays were performed by positioning a droplet of spores on the middle of a PDA or CDA plate, growing the fungus at 25°C for 5 days, and measuring the colony diameter.

GFP assay

GFP fluorescence was measured on a Fluostar optima platereader (BMG Labtech). For this dilutions of 10^8, 10^7, 10^6 and 10^5 microconidia per ml were made and 200 μl of each suspension was pipetted in a sterile, flat bottom, black 96 well plate (Greiner). The plate was shortly mixed and measured from the bottom, with 470–10 nm excitation and a 510–10 nm emission filter. The plates were kept o/n at 25°C, and measured again the next day, same settings. No differences were observed between the days (apart from a slight increase in fluorescence due to growth).

DNA-binding arrays

Details of the design and use of PBMs have been described elsewhere [38,67–69]. Here, we used two different universal PBM array designs, designated 'ME' and 'HK', after the initials of their designers, as described in [70]. Briefly, we used 150 ng of plasmid DNA in a 15 μl in vitro transcription and/or translation reaction using a PURExpress In Vitro Protein Synthesis Kit (New England BioLabs) supplemented with RNase inhibitor and 50 μM zinc acetate. After a 2-h incubation at 37°C, 15 μl of the mix was added to 155 μl of protein-binding solution for a final mix of PBS/2% skim milk/0.2 mg per ml BSA/50 μM zinc acetate/0.1% Tween-20. This mixture was added to an array previously blocked with PBS/2% skim milk and washed once with PBS/0.1% Tween-20 and once with PBS/0.01% Triton-X 100. After a 1-h incubation at room temperature, the array was washed once with PBS/0.5% Tween-20/50 mM zinc acetate and once with PBS/0.01% Triton-X 100/50 mM zinc acetate. Cy5-labeled anti-GST antibody was added, diluted in PBS/2% skim milk/50 mM zinc acetate. After a 1-h incubation at room temperature, the array was washed three times with PBS/0.05% Tween-20/50 mM zinc acetate and once with PBS/50 mM zinc acetate. The array was then imaged using an Agilent microarray scanner at 2 μm resolution. Images were scanned at two power settings: 100% photomultiplier tube (PMT) voltage (high), and 10% PMT (low). The two resulting grid images were then manually examined, and the scan with the fewest number of saturated spots was used. Image spot intensities were quantified using ImaGene software (BioDiscovery).

Calculation of spot intensities was done as described in [38]. In summary, bad spots (spots that had scratches, dust flecks or other imperfections) were flagged manually and removed from subsequent analysis. The PBM signal intensity at each spot was normalized by the corresponding amount of dsDNA. To correct for any possible nonuniformities in hybridization, these normalized PBM intensities were then adjusted according to their positions on the microarray. Each spot was considered to be at the center of a block of spots [70]. The difference between the median normalized intensity of the spots within the block and the median normalized intensity of all spots on the microarray was subtracted from the normalized intensity at that particular spot.

Calculation of 8-mer Z- and E-scores was performed as previously described [38,71]. Z-scores are derived by taking the average spot intensity for each probe containing the 8-mer, then subtracting the median value for each 8-mer, and dividing by the standard deviation, thus yielding a distribution with a median of zero and a standard deviation of one. E-scores are a modified version of the AUROC statistic, which consider the relative ranking of probes containing a given 8-mer, and range from −0.5 to +0.5, with $E > 0.45$ taken as highly statistically significant [67].

DNA-binding site inference

DNA binding sites were determined as described in [72]. The oligo-binding array returns for each transcription factor a set of eightmers and corresponding E-scores that indicate the likelihood that the protein binds this eightmer. This set of eightmers contains both the forward and reverse binding eightmers and may represent different binding motifs for a single transcription factor. Hence to infer binding motifs for a transcription factor from a set of eightmers, we need to first cluster eightmers into similar groups, where we expect at least two clusters ('forward' and 'reverse') for each transcription factor, unless the binding motif is a palindrome. First we remove unreliable eightmers (those that have a score < 0.45). We then perform pairwise Smith-Waterman alignments using the water program from the EMBOSS package (with options: -nobrief -gapopen 5.0 -gapextend 2.0) to obtain a sequence

similarity measure (the Smith-Waterman score) for each eightmer-pair. We take 40.0 –the Smith-Waterman score as a pairwise distance and use hierarchical clustering as implemented in scipy (average linkage: UPGMA) to obtain a hierarchical clustering of the eightmers. We split the resulting clustering trees into two clusters and manually checked whether these correspond to 'forward' and 'reverse' strands. We find that this is the case for all transcription factors except FOXG_15625 that probably has a palindromic binding site and FOXG_04904 for which we did not find 'reverse' strand eightmers. In the cases where we could identify 'forward' and 'reverse' strands we added the reverse complement of 'forward' strand sequences to 'reverse' strand sequences, in other cases we simply merged both clusters as they were. We aligned the sequences and obtained a sequence logo (as shown in **Fig 3A**) for these alignments with WebLogo [73].

DNA binding site enrichment analysis

The following sequence motifs were used to search the regions 1000 bp upstream or 1000bp downstream of the annotated transcriptional start site (Fusarium Comparative Sequencing Project, Broad Institute of Harvard and MIT (http://www.broadinstitute.org/), annotation 3); aTF2: CAAAC, cTF4: AGCC[A,G,C,T]TA, aTF5: CACGT, aTF7: G[A,G,C]GGCT, aTF1:T[A,G]CCG, SGE1: TTA[A,G][A,G][G,C]TT, effector motif: AACTGCCGA. For the upstream regions we used fasta files with promoter regions that we downloaded from the Broad Institute. We used custom Python scripts to append promoter regions for *SIX* genes that were not part of the annotation, based on the reported locations in [34]. We used custom Python scripts to make a fasta file with sequences that correspond to the first 1000 bp downstream from the first ATG based on the transcript gtf-file downloaded from the Broad Institute or–for *SIX* genes that were not part of the annotation–based on locations reported in [34]. We counted the number of genes with one or more motifs in the upstream regions, forward and reverse orientation separately. We did the same for two or more motifs and three or more motifs. Significant enrichment of genes with binding sites in the upstream regions in certain subgroups (accessory genes, plant-induced genes, etc.) was tested with a Hypergeometric test, with a P value < 0.01 after Bonferroni correction.

Phylogenetic trees

To define TF families we used blastp to search for homologs in 12 *Fusarium oxysporum* species (Fusarium Comparative Sequencing Project, Broad Institute of Harvard and MIT (http://www.broadinstitute.org/)[74]. We only included hits that have an E-value < 1e-5 and for which the alignment returned by BLAST spans more than 60% of both the query and the subject sequence. We used a custom Python script to cluster all hits into families using single linkage clustering and used Clustal Omega to construct multiple sequence alignments per family [75]. We trimmed the alignments using trimAl (-strictplus) [76], inspected and manually curated them. We used PhyML (with options: -q -b -2 -v e -a e) to infer phylogenies [77]. For large families we pruned the tree such that we keep the last common ancestor of the TFs that lie on chromosome 14 and their nearest neighbour that lies on the core, as root of the pruned tree.

For each of the seven transcription factor families tested for DNA binding, we searched for the occurrence of conserved domains from the Pfam database (version 27.0) using hmmscan from the hmmer package. We manually checked for presence of residues that are conserved in the Pfam seed alignment of the DNA binding domain in the multiple sequence alignments (**S3 Fig**).

Transposable/repetitive element list

We compiled a list of sequences corresponding to putative transposable elements by extracting DNA sequences for elements identified in a thorough analysis of chromosome 14 [34]. We combined these elements by elements identified by running RepeatMasker (with RepBase19.11) and extracting all sequences that were not denoted as a low-complexity region or simple repeat. We filtered out multiple occurrences of identical sequences.

LacZ activation assay

For the transcription activation assay in yeast, plasmids (Ptef1-expression vector [B3909] with and without *WOR1*, Wor1 DNA binding site–WT, mutated or empty–in front of UAS-less CYC1 promoter followed by the LacZ reporter gene [B3946]) and strains (*S. cerevisiae* Sigma 2000 ΔYEL007 /ΔYHR177) were a kind gift from Alexander D. Johnson and are described in [42]. To express *SGE1* in yeast, the *SGE1* ORF was amplified from gDNA and cloned behind the *TEF1* promoter in plasmid B3909 using SpeI and XhoI restriction enzymes. To express *aTF1* or *cTF1* in yeast, for each gene both exons were amplified from gDNA and fused together in an overlap PCR, to create an intronless sequence. This sequence was then cloned behind the *TEF1* promoter in plasmid B3909 using SpeI and SalI restriction enzymes. To clone the Tf1 DNA binding site (WT or mutated), oligo pairs were ordered corresponding to part of the *SIX1* promoter (-335 to –290 relative to the ATG) containing two Tf1 motifs flanked by 5 bp on each end plus sticky ends corresponding to the XhoI restriction site. Oligos were phosphorylated using T4 PNK (Fermentas), ligated into XhoI-digested and phosphatase treated B3946 plasmid. All plasmids were sequenced to verify the insert sequence and orientation. Oligos are listed in **S1 Data, tab: 'primers'**.

Plasmids containing *WOR1*, *SGE1*, *aTF1* or *cTF1* and LacZ reporter plasmids were co-transformed to yeast strain Sigma 2000 ΔYEL007 /ΔYHR177 according to [78]. LacZ activity was assayed as follows. Transformed yeast cells were grown in SD medium lacking Uracil and Histidine. Before cells were harvested, OD600 was measured and cells were pelleted. The cell pellet was resuspended in 150μl Z-buffer with β-mercaptoethanol (60 mM Na_2HPO_4, 40 mM NaH_2PO_4, 10 mM KCl, 1mM $MgSO_4$, 1 mM β-mercaptoethanol, pH 7), 50 μl chloroform and 20 μl 0.1% SDS were added, tubes were vortexed for 15 seconds and 700 μl pre-warmed (30°C) Z-buffer (60 mM Na_2HPO_4, 40 mM NaH_2PO_4, 10 mM KCl, 1mM $MgSO_4$, pH 7) with ONPG (1 mg/ml) was added at t = 0. Tubes were incubated at 30°C until the reaction started to turn yellow. The reaction was stopped with 500 μl 1M Na_2CO_3 and the time recorded, Tubes were centrifuged 2 min. at 14000 rpm and the OD of the supernatant was measured at 420 nm. Miller units were calculated as follows: (A420*1000)/(A600*minutes*ml culture).

Deletion of *SGE1* in *aTF1* overexpressor background

To make *aTF1* overexpressors in the Fol4287 wild type background, the hygromycin resistance cassette (*HPH*) in the plasmid described above (Pfem1aTF1-*HPH* cassette) was exchanged for a phleomycine resistance cassette (*BLE*), using XbaI plus PacI (insert) and SpeI plus PacI (vector) restriction enzymes. The resulting plasmid (Pfem1aTF1-*BLE* cassette) was transformed to Fol4287 and transformants were selected on zeocine. An empty plasmid (pRW1p: containing only the *BLE* cassette [79]) was transformed to Fol4287 in parallel, as a negative control. Ten independent zeocine resistant colonies of each transformation were monospored and checked for *SIX1* and *SIX3* expression. For this, liquid cultures (100 ml 1% KNO_3, 3% sucrose, 0.17% YNB w/o NH_4 and aa.) were inoculated from plate and mycelium was harvested after 5 days at 25°C, 150–175 rpm. RNA isolation, cDNA synthesis and Q-RT-PCR were performed as described above.

SIX1 and *SIX3* levels were normalized to *EF1-α*. Two out of ten *aTF1* transformants showed induction of *SIX1* and *SIX3* expression. One of those was selected for deletion of *SGE1*.

Deletion of *SGE1* in the selected Fol4287 *aTF1* overexpressor was done as described in [26], using the same deletion construct and the same PCR control for deletion of *SGE1*. To check transformants (ectopic and *in locus*) for *SIX1* and *SIX3* expression, liquid cultures (100 ml 1% KNO_3, 3% sucrose, 0.17% YNB w/o NH_4 and aa.) were inoculated from plate and mycelium was harvested after 5 days at 25°C, 150–175 rpm. RNA isolation, cDNA synthesis and Q-RT-PCR were performed as described above. *SIX1*, *SIX3*, *SGE1* and *aTF1* levels were normalized to *EF1-α*. Primers used are listed in **S1 Data, tab: 'primers'**.

Supporting Information

S1 Fig. Transcription factors encoded on accessory chromosomes of *F. oxysporum* have diverged more than core-encoded transcription factors between *Fusarium* species. Phylogenetic trees of the protein sequences of the different transcription factor families, including homologs in Fol, other *F. oxysporum ff. spp*. and other *Fusarium* species. **A)** Family of aTf4 (FOXG_14201), a C2H2 zinc finger transcription factor—this transcription factor has no additional accessory homologs in Fol. **B)** aTf3 (FOXG_17266), a C2H2 zinc finger transcription factor with no homologs on the core genome, and only two homologs in accessory regions in other *formae speciales*. **C)** Family of aTf6 (FOXG_14211), a C2H2 zinc finger transcription factor—this transcription factor has no additional accessory homologs in Fol. **D)** Family of aTf7 (FOXG_14275), a C2H2 zinc finger transcription factor. This transcription factor has seven additional accessory homologs in Fol4287. **E)** Protein family of aTf1 (Ftf1; FOXG_14257, FOXG_17458, FOXG_16414) of Zn(2)Cys(6) zinc finger transcription factors. This transcription factor has seven additional accessory homologs in Fol4287. **F)** Family of aTf8 (FOXG_14277), a Zn(2)Cys(6) zinc finger transcription factor. This transcription factor has five additional accessory homologs in Fol4287. **G)** Family of aTf9 (FOXG_14274, FOXG_14137, FOXG_14220) of bZIP leucine zipper transcription factors. This family has seven additional accessory homologs in Fol4287. **H)** Family of aTf2 (FOXG_17260), a forkhead transcription factor with one homolog on the core genome and one homolog on the accessory chromosomes. Only three other forkhead transcription factor genes are present in the Fol4287 genome. **I)** Family of aTf5 (FOXG_14230), a bZIP leucine zipper transcription factor—this transcription factor has no additional accessory homologs in Fol.
(PDF)

S2 Fig. Predicted translational start sites of *TF1* homologs in Fol. In-frame potential startcodons (ATG) are indicated with red arrows and are bold and underlined, other ATG codons are bold. Red bases indicate single basepair indels. The following genes have a 'long' predicted ORF, according to the Broad annotation (the number refers to the FOXG number of the Broad annotation): 09390, cTF1, core genome; 17458, pathogenicity chromosome, used for overexpression & RNAseq; 14257, pathogenicity chromosome, 14422, accessory chromosome 15; 15059, accessory region on chromosome 1. The remaining genes have a shorter predicted ORF according to the Broad annotation, namely: 16414, pathogenicity chromosome; 17123, accessory chromosome 6, 17084, accessory chromosome 3/6, used for overexpression; 14000, accessory chromosome 3/6; 12589, accessory chromosome 3/6, 12539, accessory chromosome 3/6. The genes with a long predicted ORF start at the ATG indicated with red arrow number 1. All remaining genes have a 17 base pair deletion (compared to the core homolog *cTF1*/>09390), removing this ATG. Their predicted startcodon is the next in-frame ATG (red arrow 2). In addition, all homologs with the 17 bp deletion also have a more upstream stop codon– 300 bp upstream of the stopcodon in *cTF1*. This means that all ORFs starting at the position indicated

by red arrow 2, start 117 bp later and stop 300 bp earlier than the long *TF1* homologs, rendering ORFs of around 2800 bp. These genes we refer to as having a short ORF. The ORFs starting at the position indicated by red arrow 1 are around 3200 bp. These genes we refer to as having a long ORF.
(PDF)

S3 Fig. DNA binding domain alignments of the transcription factors tested for DNA binding specificity. For each family we predicted domain architecture for its members. We here show for each family, from the multiple sequence alignment used to generate its phylogeny (S1 Fig), those regions that correspond to a DNA binding domain. The background color of a residue corresponds to its conservation in the alignment, where darker colors indicate more conservation. Residues that are conserved in the seed alignment from Pfam and are present in at least two sequence are indicated in bold on top of the alignment. Residues that are conserved in the Pfam seed alignment but are not present in at least two sequences are in normal font. Gene names correspond to names and species or *ff. spp*. mentioned in Fig 2 and S1 Fig. Core-encoded transcription factors of *F. oxysporum* (Fol4287), *F. verticillioides*, *F. graminearum* and *F. solani* are indicated in red. Accessory transcription factors from Fol tested for DNA binding specificity are indicated in bold.
(PDF)

S4 Fig. Enrichment of transcription factor DNA binding sites in promoter regions and coding regions. A) The number of genes with one or more transcription factor DNA binding site (TF DBS) in the 1000 bp upstream of the predicted transcriptional start site, for the complete genome, and for a subset of genes (up- or down-regulated during infection). Boxes indicate a significant enrichment (p value <0.01 after Bonferroni correction). The motif found in effector gene promoters earlier (aacTGCCGa and overlapping with the Tf1 DNA binding site has been included. For all categories the total number of genes is given (ALL, green), the number of core genes (CORE, blue) and the number of accessory genes (purple). Accessory genes are subdivided in total (ACC), accessory genes not on the pathogenicity chromosome (no14) and the accessory genes on the pathogenicity chromosome (only_14). **B)** As A, but for the 1000 bp downstream the ATG. **C**) As A, but only for the Tf1 DNA binding site ($\geq$3 occurrences), the motif found in the promoters of effector genes, overlapping with the Tf1 DNA binding site ($\geq$1 occurrences) and the Sge1 DNA binding site ($\geq$1 occurrences). The following categories are additional: genes UP or DOWN regulated in the *aTF1*, *cTF1* or *SGE1* overexpressors, genes for small secreted proteins, genes with a MIMP in their promoter, genes coding for proteins found in xylem sap of infected plants. **D**) As C, but for the 1000 bp downstream the ATG.
(PDF)

S5 Fig. Screening and characterization of transcription factor overexpressors. A) Estimated percentage, by visual inspection, of GFP-fluorescent spores in independent transformants of different transcription factor overexpression constructs. x: this transformant does not exist (misnumbered), *: of the background strain (Psix1GFP—WT) the same transformant was tested multiple times, each number represents therefore a replicate instead of a different transformant. **B)** Relative expression of different transcription factor genes in WT and their respective overexpressors as determined by Q-RT-PCR. Relative expression is calculated as TF expression/expression of *EF1α*. Minimally three independent transformants were tested per transcription factor. Error bars indicate the standard deviation of two technical replicates. **C)** Growth rate of the different transcription factor overexpressors. Minimally three independent transformants were tested per transcription factor, on both rich medium (potato dextrose agar, PDA, top panel) and complete minimal medium (Czapek Dox Agar, CDA, bottom panel).

Stars indicate significant differences compared to the background strain (T-test, $p<0.01$). Error bars indicate the standard deviation of minimally two independent replicates. **D)** Bioassay on susceptible tomato plants. Minimally three independent transformants were tested per transcription factor. Control treatments were: mock, WT (this is the WT used to make the background strain, Fol007), Psix1GFP (the background strain, WT + reporter construct Psix1GFP), Psix1GFP + RFP (the background strain plus a constitutively expressed RFP construct). Top panel: average plant weight (gram) 21 dpi. Error bars indicate standard deviation. Stars indicate a significant difference from the background strain (Psix1GFP, T-test, $p<0.01$). Bottom panel: disease index (0–4 arbitrary units) 21 dpi.
(PDF)

S6 Fig. Overlap between sets of differentially expressed genes. A) Top three Venn diagrams show the overlap of sets of differentially expressed genes between two independent transformants of each transcription factor. The overlapping genes are depicted in red and these are the genes considered for all further analyses. Lower six Venn diagrams: overlap between target genes in the different overexpressors, up-regulated (left three diagrams) or down-regulated (right three diagrams). **B&C)** Number of up- or down-regulated genes in different subcategories. Significant enrichment or depletion in each subcategory was tested with a hypergeometric test, P value < 0.01 after Bonferroni correction. Orange cells: enrichment, blue cells: depletion. Columns: whole genome (all genes), planta UP (genes up-regulated during infection), aTF1 UP, cTF1 UP, SGE1 UP (genes up-regulated in the *aTF1*, *cTF1* or *SGE1* overexpressors, a1c1S1 UP (genes up-regulated in all three overexpressors), planta DOWN (genes down-regulated during infection), aTF1 DOWN, cTF1 DOWN, SGE1 DOWN (genes down-regulated in the *aTF1*, *cTF1* or *SGE1* overexpressors, a1c1S1 DOWN (genes down-regulated in all three overexpressors), MIMP (genes with a miniature impala transposable element (MIMP) in their promoter), XS proteins (genes of which the protein product was found in the xylem sap of infected tomato plants [34]). Rows: all (all genes), core (all genes located on the core genome), pathogenicity chr. (all genes on the pathogenicity chromosome), other accessory chr. (all accessory genes, except those on the pathogenicity chromosome), in planta UP all (all genes up-regulated during infection), UP core (genes located on core chromosomes and up-regulated during infection), UP pathogenicity chr. (genes located on the pathogenicity chromosome and up-regulated during infection), UP accessory chr. (genes on the accessory regions, except those on the pathogenicity chromosome, and up-regulated during infection), in planta DOWN (all genes down-regulated during infection), SIX genes (all *SIX* genes), XS proteins (genes of which the protein product was found in the xylem sap of infected tomato plants [34]), XS proteins w/o SIX proteins (genes coding for xylem sap proteins minus the *SIX* genes), mimp (genes with a MIMP in their promoter), mimp on accessory chr. (genes on the accessory regions with a MIMP in their promoter), mimp on pathogenicity chr. (genes on the pathogenicity chromosome with a MIMP in their promoter), mimp on core (genes on the core chromosomes with a MIMP in their promoter), mimp w/o SIX genes (genes with a MIMP in their promoter, without the *SIX* genes), mimp on path. chr. w/o SIX (genes on the pathogenicity chromosome with a MIMP in their promoter, without the *SIX* genes). **D)** Number of up- or down-regulated transposable element transcripts in different subcategories. All sequencing reads from the overexpressors and reads from infected plant material were mapped to a fasta file in which the sequence of each repetitive element, plus each previously annotated transposable element, is present once. The number of reads was normalized to the number of reads mapped uniquely to the whole genome for each sample. The number of reads mapping to each different sequence was counted and differentially expressed sequences were called. Significant enrichments or depletions were tested with a hypergeometric test, P value < 0.01 after Bonferroni correction.

Orange cells: enrichment, blue cells: depletion. Columns: ALL (all genes or transposable elements), UP (up-regulated genes or transposable elements), DOWN (down-regulated genes or transposable elements), aTF1A & aTF1E (two independent *aTF1* overexpressors), cTF1C & cTF1D (two independent *cTF1* overexpressors), SGE15 &SGE1M (two independent *SGE1* overexpressors), *in planta* (Fol sampled from infected plant material). Accessory; all genes located on the accessory regions, TE: transposable elements. Note that the reads mapping to a single sequence may be derived from many different locations on the genome, so each sequence represents 'net' expression. Because most transposable elements are located on accessory chromosomes, comparisons were made to those regions specifically. The portion of repetitive/ transposon sequences down-regulated in the different transformants is very similar to the portion of accessory genes down-regulated. The number of transposons up-regulated in the different transformants is lower than the portion of genes up-regulated on the accessory chromosomes. Both may be due to inclusion of transposable elements in the large scale gene repression on the accessory chromosomes, given that we look at the net expression for each sequence. Also during infection—tested with a strain that does not show the large accessory gene repression—no increased up-regulation of transposon transcripts is observed.
(PDF)

S7 Fig. Relative expression of *aTF1*, *cTF1* and *SGE1* in WT, their respective overexpressors and during infection. Relative expression was determined by Q-RT-PCR, and calculated as TF expression /expression of *EF1α*. Two independent transformants were tested per transcription factor. Plants were infected with strain Fol007 and roots were harvested for RNA extraction 9 dpi. Error bars indicate the standard error of two (during infection) or three (all other samples) independent biological replicates.
(PDF)

S8 Fig. Correlation between the transcription factors expressed from the *FEM1* promoter and changes in gene expression. A) Relative expression of *aTF1*, *GFP* and *SIX3* compared to expression of the reference gene *EF1α* determined by Q-RT-PCR. Expression was measured in nine different independent *aTF1* overexpressors (a, b, c, e, f, g, j, k and o) and in the background strain (Psix1GFP). *aTF1* is expressed from the endogenous locus and–in the overexpressors–from an additional overexpression construct. *GFP* is expressed by the *SIX1* promoter, from the original locus. *SIX1* and *SIX3* are representative effector genes. Relative expression is calculated as gene expression/expression of *EF1α*. Error bars indicate standard deviation. **B)** clustering of differentially expressed genes ($P_{adjusted} < 0.01$) after expression of *aTF1*, *cTF1* or *SGE1* from the *FEM1* promoter, by log2 fold change values for each differentially expressed gene. The log2 fold change values of genes that are not significantly different from the control are set to zero. Two independent transformants were studied per transcription factor, resulting in six independent conditions. The tree reflects the similarity between the transcriptional changes in each sample.
(PDF)

S9 Fig. Expression of accessory genes in *aTF1*, *cTF1* or *SGE1* overexpressors is reduced to a level that is also observed in some wild type strains. Heatmap of differentially expressed genes during infection or after in *aTF1*, *cTF1* or *SGE1* overexpressors, divided per subgenome (the pathogenicity chromosome, other accessory regions and core genome). Displayed is the log2 fold change for each differentially expressed gene (rows), with yellow indicating up-regulation compared to the control (hues cover the log2 fold range from 0 to 5). Blue indicates down-regulation compared to the control (hues cover the log2 fold range from 0 to -5). The log2 fold change values of genes that are not significantly different from the control are set to

zero. Each condition is a separate column, with two independent transformants (thus 2 columns) per transcription factor. The order of the rows reflects clustering of similar expression patterns. The first seven columns are the same as in Fig 8A, the eighth column is additional (column header highlighted in red). Wild type strain Fol4287 was used for *in planta* samples, and for Fol4287 samples from flask (compared to each other in the first column). Fol007 is a wild type strain very similar to Fol4287 and was used to create the background strain (Fol007 + Psix1GFP reporter construct) for the overexpressors. Overexpressors and their background strain were compared in column two to seven. The eighth column compares the two wild type strains to each other (Fol4287 and Fol007+Psix1GFP). The red bar highlights the group of genes that is expressed lower in Fol 4287 WT and in the overexpressors in Fol 007 + Psix1GFP background, compared to Fol007 + Psix1GFP.
(PDF)

S10 Fig. In two different transformants the same region of the pathogenicity chromosome is up-regulated. Expression (of the background strain, top row, green) or the difference in expression with the background strain (of the overexpressors, six bottom rows, different shades of red) in RPKM or ΔRPKM (log10 scale). On the x-axis, the genes of SC 22, SC 36 and SC 51 are plotted, in the same order as they appear on the supercontig (SC). Only positive numbers are shown for the overexpressors (ΔRPKM)–down-regulated genes are not visible. No distinction has been made between significant and non-significant up-regulation—all differences are shown. The second and third rows show two independent *aTF1* overexpressors (dark red), the fourth and fifth rows show two independent *cTF1* overexpressors (bright red), the sixth and seventh rows show two independent *SGE1* overexpressors (orange). The dashed box indicates the region that is up-regulated in one *cTF1* overexpressor and one *SGE1* overexpressor.
(PDF)

S11 Fig. The overall amount of transposable element-derived transcripts is unaltered in *SGE1*, *aTF1* and *cTF1* overexpressors. All sequencing reads from the overexpressors and reads from infected plant material were mapped to a fasta file where the sequence of each repetitive element, plus all each previously annotated transposable element, is present once. The number of reads was normalized to the number of reads mapped uniquely to the whole genome for each sample. In the graph, the total number of reads assigned to transposable elements per 20 million reads uniquely mapped to the reference genome are shown. Of each transcription factor overexpressor (*aTF1*, *cTF1* or *SGE1*) two independent transformants are shown. No consistent significant effects on overall transposon expression were observed in any of the overexpressors. However, the total amount of transposon-derived reads during infection was reduced.
(PDF)

S12 Fig. Deletion of *SGE1* renders Fol non-pathogenic, even in an *aTF1* overexpressor. Bioassay on susceptible tomato plants. Two independent transformants were tested for both ectopic and *in locus* integration of the *SGE1* deletion construct in the *aTF1* overexpressor (aTF1 OE ectopic and aTF1 *Δsge1*, respectively). Control treatments were: mock, Fol4287 (WT), *Δsge1* (*SGE1* deletion mutant in Fol4287), aTF1 OE (*aTF1* overexpressor in Fol4287). Left panel: average plant weight (gram) 21 dpi. Error bars indicate standard deviation. Right panel: disease index (0–4 arbitrary units) 21 dpi.
(PDF)

S1 Data. Background information. This file contains an overview of the transcription factors mentioned in this study, an overview of the results of the DNA binding assay, an overview of DNA binding site occurrences, overviews of genes up- or down-regulated in *aTF1*, *cTF1* and

SGE1 overexpressors, an overview of genes with a MIMP within 2 kb upstream of the ATG, genes of which the protein product is detected in xylem sap and genes up- or down-regulated *in planta*, a list of the primers used in this study and the mapped read counts + RPKM values for all RNA-seq samples.
(XLSX)

Acknowledgments

The authors acknowledge Ernst-Jan Eggers for pioneering and contributing in generating *SGE1* overexpressors. We thank Alexander Johnson and Matthew Lohse for kindly sharing plasmids and strains, and Peter van Dam for his generous assistance in managing the RNAseq data and performing the MIMP search.

More than grateful we are for the help of Petra M. Houterman, Wim de Leeuw, Huub Hoefsloot and Antje van der Does-Bianchi in the RNAseq experiment, for Mila C. Blekemolen, Nico Tintor, Marijn Knip, Ludek Tikovsky and Harrold Lemereis, for their help in performing the bioassays, for Gertien Smits and her laboratory, who have been very kind and helpful in assisting measurements with their GFP platereader and, finally, to Ben J.C. Cornelissen, Nico Tintor and Harrold A. van den Burg for helpful suggestions and critical reading of the manuscript.

Author Contributions

Conceptualization: HCvdD MR.

Formal analysis: HCvdD LF AY SMS TRH.

Funding acquisition: HCvdD.

Investigation: HCvdD LF AY LL SMS JML.

Project administration: HCvdD MR.

Supervision: TRH MR.

Visualization: HCvdD LF.

Writing – original draft: HCvdD LF.

Writing – review & editing: HCvdD LF AY MR.

References

1. Soyer JL, El Ghalid M, Glaser N, Ollivier B, Linglin J, et al. (2014) Epigenetic control of effector gene expression in the plant pathogenic fungus *Leptosphaeria maculans*. PLoS genetics 10: e1004227. doi: 10.1371/journal.pgen.1004227 PMID: 24603691
2. Lo Presti L, Lanver D, Schweizer G, Tanaka S, Liang L, et al. (2015) Fungal effectors and plant susceptibility. Annu Rev Plant Biol 66: 513–545. doi: 10.1146/annurev-arplant-043014-114623 PMID: 25923844
3. Schotanus K, Soyer JL, Connolly LR, Grandaubert J, Happel P, et al. (2015) Histone modifications rather than the novel regional centromeres of *Zymoseptoria tritici* distinguish core and accessory chromosomes. Epigenetics Chromatin 8: 41. doi: 10.1186/s13072-015-0033-5 PMID: 26430472
4. Croll D, McDonald BA (2012) The accessory genome as a cradle for adaptive evolution in pathogens. PLoS Pathog 8: e1002608. doi: 10.1371/journal.ppat.1002608 PMID: 22570606
5. Grandaubert J, Lowe RG, Soyer JL, Schoch CL, Van de Wouw AP, et al. (2014) Transposable element-assisted evolution and adaptation to host plant within the *Leptosphaeria maculans-Leptosphaeria biglobosa* species complex of fungal pathogens. BMC Genomics 15: 891. doi: 10.1186/1471-2164-15-891 PMID: 25306241

6. Chuma I, Isobe C, Hotta Y, Ibaragi K, Futamata N, et al. (2011) Multiple translocation of the AVR-Pita effector gene among chromosomes of the rice blast fungus Magnaporthe oryzae and related species. PLoS Pathog 7: e1002147. doi: 10.1371/journal.ppat.1002147 PMID: 21829350

7. Ma LJ, van der Does HC, Borkovich KA, Coleman JJ, Daboussi MJ, et al. (2010) Comparative genomics reveals mobile pathogenicity chromosomes in *Fusarium*. Nature 464: 367–373. doi: 10.1038/nature08850 PMID: 20237561

8. Akagi Y, Akamatsu H, Otani H, Kodama M (2009) Horizontal chromosome transfer, a mechanism for the evolution and differentiation of a plant-pathogenic fungus. Eukaryot Cell 8: 1732–1738. doi: 10.1128/EC.00135-09 PMID: 19749175

9. Friesen TL, Stukenbrock EH, Liu Z, Meinhardt S, Ling H, et al. (2006) Emergence of a new disease as a result of interspecific virulence gene transfer. Nat Genet 38: 953–956. doi: 10.1038/ng1839 PMID: 16832356

10. Coleman JJ, Rounsley SD, Rodriguez-Carres M, Kuo A, Wasmann CC, et al. (2009) The genome of *Nectria haematococca*: contribution of supernumerary chromosomes to gene expansion. PLoS genetics 5: e1000618. doi: 10.1371/journal.pgen.1000618 PMID: 19714214

11. Noguchi MT, Yasuda N, Fujita Y (2006) Evidence of Genetic Exchange by Parasexual Recombination and Genetic Analysis of Pathogenicity and Mating Type of Parasexual Recombinants in Rice Blast Fungus, Magnaporthe oryzae. Phytopathology 96: 746–750. doi: 10.1094/PHYTO-96-0746 PMID: 18943148

12. O'Connell RJ, Thon MR, Hacquard S, Amyotte SG, Kleemann J, et al. (2012) Lifestyle transitions in plant pathogenic *Colletotrichum* fungi deciphered by genome and transcriptome analyses. Nature genetics 44: 1060–1065. doi: 10.1038/ng.2372 PMID: 22885923

13. Schilling L, Matei A, Redkar A, Walbot V, Doehlemann G (2014) Virulence of the maize smut *Ustilago maydis* is shaped by organ-specific effectors. Molecular plant pathology 15: 780–789. PMID: 25346968

14. Stergiopoulos I, de Wit PJ (2009) Fungal effector proteins. Annu Rev Phytopathol 47: 233–263. doi: 10.1146/annurev.phyto.112408.132637 PMID: 19400631

15. Torreblanca J, Stumpferl S, Basse CW (2003) Histone deacetylase Hda1 acts as repressor of the *Ustilago maydis* biotrophic marker gene *mig1*. Fungal Genet Biol 38: 22–32. PMID: 12553933

16. Zheng Y, Kief J, Auffarth K, Farfsing JW, Mahlert M, et al. (2008) The *Ustilago maydis* Cys2His2-type zinc finger transcription factor Mzr1 regulates fungal gene expression during the biotrophic growth stage. Mol Microbiol 68: 1450–1470. doi: 10.1111/j.1365-2958.2008.06244.x PMID: 18410495

17. Heimel K, Scherer M, Vranes M, Wahl R, Pothiratana C, et al. (2010) The transcription factor Rbf1 is the master regulator for b-mating type controlled pathogenic development in Ustilago maydis. PLoS Pathog 6: e1001035. doi: 10.1371/journal.ppat.1001035 PMID: 20700446

18. Zahiri A, Heimel K, Wahl R, Rath M, Kamper J (2010) The Ustilago maydis forkhead transcription factor Fox1 is involved in the regulation of genes required for the attenuation of plant defenses during pathogenic development. Mol Plant Microbe Interact 23: 1118–1129. doi: 10.1094/MPMI-23-9-1118 PMID: 20687802

19. Wahl R, Zahiri A, Kamper J (2010) The Ustilago maydis b mating type locus controls hyphal proliferation and expression of secreted virulence factors in planta. Mol Microbiol 75: 208–220. doi: 10.1111/j.1365-2958.2009.06984.x PMID: 19943901

20. IpCho SV, Tan KC, Koh G, Gummer J, Oliver RP, et al. (2010) The transcription factor StuA regulates central carbon metabolism, mycotoxin production, and effector gene expression in the wheat pathogen *Stagonospora nodorum*. Eukaryot Cell 9: 1100–1108. doi: 10.1128/EC.00064-10 PMID: 20495056

21. Soyer JL, Hamiot A, Ollivier B, Balesdent MH, Rouxel T, et al. (2015) The APSES transcription factor LmStuA is required for sporulation, pathogenic development and effector gene expression in Leptosphaeria maculans. Mol Plant Pathol 16: 1000–1005. doi: 10.1111/mpp.12249 PMID: 25727237

22. Huang G, Wang H, Chou S, Nie X, Chen J, et al. (2006) Bistable expression of *WOR1*, a master regulator of white-opaque switching in *Candida albicans*. Proc Natl Acad Sci U S A 103: 12813–12818. doi: 10.1073/pnas.0605270103 PMID: 16905649

23. Michielse CB, Studt L, Janevska S, Sieber CM, Arndt B, et al. (2015) The global regulator FfSge1 is required for expression of secondary metabolite gene clusters but not for pathogenicity in *Fusarium fujikuroi*. Environmental microbiology 17: 2690–2708. doi: 10.1111/1462-2920.12592 PMID: 25115968

24. Jonkers W, Dong Y, Broz K, Kistler HC (2012) The Wor1-like protein Fgp1 regulates pathogenicity, toxin synthesis and reproduction in the phytopathogenic fungus *Fusarium graminearum*. PLoS pathogens 8: e1002724. doi: 10.1371/journal.ppat.1002724 PMID: 22693448

25. Brown DW, Busman M, Proctor RH (2014) *Fusarium verticillioides SGE1* is required for full virulence and regulates expression of protein effector and secondary metabolite biosynthetic genes. Mol Plant Microbe Interact 27: 809–823. doi: 10.1094/MPMI-09-13-0281-R PMID: 24742071

26. Michielse CB, van Wijk R, Reijnen L, Manders EM, Boas S, et al. (2009) The nuclear protein Sge1 of *Fusarium oxysporum* is required for parasitic growth. PLoS Pathog 5: e1000637. doi: 10.1371/journal.ppat.1000637 PMID: 19851506

27. Michielse CB, Becker M, Heller J, Moraga J, Collado IG, et al. (2011) The *Botrytis cinerea* Reg1 protein, a putative transcriptional regulator, is required for pathogenicity, conidiogenesis, and the production of secondary metabolites. Mol Plant Microbe Interact 24: 1074–1085. doi: 10.1094/MPMI-01-11-0007 PMID: 21635139

28. Okmen B, Collemare J, Griffiths S, van der Burgt A, Cox R, et al. (2014) Functional analysis of the conserved transcriptional regulator CfWor1 in *Cladosporium fulvum* reveals diverse roles in the virulence of plant pathogenic fungi. Molecular microbiology 92: 10–27. doi: 10.1111/mmi.12535 PMID: 24521437

29. Santhanam P, Thomma BP (2013) *Verticillium dahliae* Sge1 differentially regulates expression of candidate effector genes. Mol Plant Microbe Interact 26: 249–256. doi: 10.1094/MPMI-08-12-0198-R PMID: 22970788

30. Mirzadi Gohari A, Mehrabi R, Robert O, Ince IA, Boeren S, et al. (2014) Molecular characterization and functional analyses of ZtWor1, a transcriptional regulator of the fungal wheat pathogen *Zymoseptoria tritici*. Mol Plant Pathol 15: 394–405. doi: 10.1111/mpp.12102 PMID: 24341593

31. Chen Y, Zhai S, Zhang H, Zuo R, Wang J, et al. (2014) Shared and distinct functions of two Gti1/Pac2 family proteins in growth, morphogenesis and pathogenicity of *Magnaporthe oryzae*. Environmental microbiology 16: 788–801. doi: 10.1111/1462-2920.12204 PMID: 23895552

32. Tollot M, Assmann D, Becker C, Altmuller J, Dutheil JY, et al. (2016) The WOPR Protein Ros1 Is a Master Regulator of Sporogenesis and Late Effector Gene Expression in the Maize Pathogen Ustilago maydis. PLoS Pathog 12: e1005697. doi: 10.1371/journal.ppat.1005697 PMID: 27332891

33. Cain CW, Lohse MB, Homann OR, Sil A, Johnson AD (2012) A conserved transcriptional regulator governs fungal morphology in widely diverged species. Genetics 190: 511–521. doi: 10.1534/genetics.111.134080 PMID: 22095082

34. Schmidt SM, Houterman PM, Schreiver I, Ma L, Amyotte S, et al. (2013) MITEs in the promoters of effector genes allow prediction of novel virulence genes in *Fusarium oxysporum*. BMC genomics 14: 119. doi: 10.1186/1471-2164-14-119 PMID: 23432788

35. de Vega-Bartol JJ, Martin-Dominguez R, Ramos B, Garcia-Sanchez MA, Diaz-Minguez JM (2011) New virulence groups in *Fusarium oxysporum* f. sp. *phaseoli*: the expression of the gene coding for the transcription factor ftf1 correlates with virulence. Phytopathology 101: 470–479. doi: 10.1094/PHYTO-09-10-0252 PMID: 21091181

36. Nino-Sanchez J, Tello V, Casado-Del Castillo V, Thon MR, Benito EP, et al. (2015) Gene expression patterns and dynamics of the colonization of common bean (*Phaseolus vulgaris* L.) by highly virulent and weakly virulent strains of *Fusarium oxysporum*. Front Microbiol 6: 234. doi: 10.3389/fmicb.2015.00234 PMID: 25883592

37. Jonkers W, Xayamongkhon H, Haas M, Olivain C, van der Does HC, et al. (2014) *EBR1* genomic expansion and its role in virulence of *Fusarium* species. Environmental microbiology 16: 1982–2003. doi: 10.1111/1462-2920.12331 PMID: 24237614

38. Berger MF, Philippakis AA, Qureshi AM, He FS, Estep PW 3rd, et al. (2006) Compact, universal DNA microarrays to comprehensively determine transcription-factor binding site specificities. Nat Biotechnol 24: 1429–1435. doi: 10.1038/nbt1246 PMID: 16998473

39. Georges AB, Benayoun BA, Caburet S, Veitia RA (2010) Generic binding sites, generic DNA-binding domains: where does specific promoter recognition come from? FASEB J 24: 346–356. doi: 10.1096/fj.09-142117 PMID: 19762556

40. Adams CC, Workman JL (1995) Binding of disparate transcriptional activators to nucleosomal DNA is inherently cooperative. Mol Cell Biol 15: 1405–1421. PMID: 7862134

41. Zhang S, Zhang T, Yan M, Ding J, Chen J (2014) Crystal structure of the WOPR-DNA complex and implications for Wor1 function in white-opaque switching of *Candida albicans*. Cell Res 24: 1108–1120. doi: 10.1038/cr.2014.102 PMID: 25091450

42. Lohse MB, Zordan RE, Cain CW, Johnson AD (2010) Distinct class of DNA-binding domains is exemplified by a master regulator of phenotypic switching in *Candida albicans*. Proc Natl Acad Sci U S A 107: 14105–14110. doi: 10.1073/pnas.1005911107 PMID: 20660774

43. Lohse MB, Rosenberg OS, Cox JS, Stroud RM, Finer-Moore JS, et al. (2014) Structure of a new DNA-binding domain which regulates pathogenesis in a wide variety of fungi. Proc Natl Acad Sci U S A 111: 10404–10410. doi: 10.1073/pnas.1410110111 PMID: 24994900

44. Alkahyyat F, Ni M, Kim SC, Yu JH (2015) The WOPR Domain Protein OsaA Orchestrates Development in Aspergillus nidulans. PLoS One 10: e0137554. doi: 10.1371/journal.pone.0137554 PMID: 26359867

45. Schmidt SM, Brouwer S, Lukasiewicz J, Fokkens L, van Dam P, et al. (submitted) The in planta transcriptomes of strains of Fusarium oxysporum specific for tomato or melon include genes for transcription factors required for full virulence.

46. van der Does HC, Duyvesteijn RG, Goltstein PM, van Schie CC, Manders EM, et al. (2008) Expression of effector gene *SIX1* of *Fusarium oxysporum* requires living plant cells. Fungal genetics and biology: FG & B 45: 1257–1264.

47. Houterman PM, Ma L, van Ooijen G, de Vroomen MJ, Cornelissen BJ, et al. (2009) The effector protein Avr2 of the xylem-colonizing fungus Fusarium oxysporum activates the tomato resistance protein I-2 intracellularly. The Plant journal: for cell and molecular biology 58: 970–978.

48. Gawehns F, Houterman PM, Ichou FA, Michielse CB, Hijdra M, et al. (2014) The Fusarium oxysporum effector Six6 contributes to virulence and suppresses I-2-mediated cell death. Molecular plant-microbe interactions: MPMI 27: 336–348. doi: 10.1094/MPMI-11-13-0330-R PMID: 24313955

49. Gawehns F, Ma L, Bruning O, Houterman PM, Boeren S, et al. (2015) The effector repertoire of Fusarium oxysporum determines the tomato xylem proteome composition following infection. Front Plant Sci 6: 967. doi: 10.3389/fpls.2015.00967 PMID: 26583031

50. van Dam P, Fokkens L, Schmidt SM, Linmans JH, Kistler HC, et al. (2016) Effector profiles distinguish formae speciales of Fusarium oxysporum. Environ Microbiol.

51. Schoffelmeer EA, Vossen JH, van Doorn AA, Cornelissen BJ, Haring MA (2001) *FEM1*, a *Fusarium oxysporum* glycoprotein that is covalently linked to the cell wall matrix and is conserved in filamentous fungi. Mol Genet Genomics 265: 143–152. PMID: 11370861

52. Anders S, Huber W (2010) Differential expression analysis for sequence count data. Genome Biol 11: R106. doi: 10.1186/gb-2010-11-10-r106 PMID: 20979621

53. Vlaardingerbroek I, Beerens B, Schmidt SM, Cornelissen BJ, Rep M (2016) Dispensable chromosomes in Fusarium oxysporum f.sp lycopersici. Mol Plant Pathol.

54. Priebe S, Kreisel C, Horn F, Guthke R, Linde J (2015) FungiFun2: a comprehensive online resource for systematic analysis of gene lists from fungal species. Bioinformatics 31: 445–446. doi: 10.1093/bioinformatics/btu627 PMID: 25294921

55. Michielse CB, van Wijk R, Reijnen L, Cornelissen BJ, Rep M (2009) Insight into the molecular requirements for pathogenicity of *Fusarium oxysporum* f. sp. *lycopersici* through large-scale insertional mutagenesis. Genome biology 10: R4. doi: 10.1186/gb-2009-10-1-r4 PMID: 19134172

56. Yu JH, Keller N (2005) Regulation of secondary metabolism in filamentous fungi. Annu Rev Phytopathol 43: 437–458. doi: 10.1146/annurev.phyto.43.040204.140214 PMID: 16078891

57. Studt L, Schmidt FJ, Jahn L, Sieber CM, Connolly LR, et al. (2013) Two histone deacetylases, FfHda1 and FfHda2, are important for *Fusarium fujikuroi* secondary metabolism and virulence. Applied and environmental microbiology 79: 7719–7734. doi: 10.1128/AEM.01557-13 PMID: 24096420

58. Connolly LR, Smith KM, Freitag M (2013) The *Fusarium graminearum* histone H3 K27 methyltransferase *KMT6* regulates development and expression of secondary metabolite gene clusters. PLoS genetics 9: e1003916. doi: 10.1371/journal.pgen.1003916 PMID: 24204317

59. Webster RH, Sil A (2008) Conserved factors Ryp2 and Ryp3 control cell morphology and infectious spore formation in the fungal pathogen *Histoplasma capsulatum*. Proc Natl Acad Sci U S A 105: 14573–14578. doi: 10.1073/pnas.0806221105 PMID: 18791067

60. Nino-Sanchez J, Casado-Del Castillo V, Tello V, de Vega-Bartol JJ, Ramos B, et al. (2016) The FTF gene family regulates virulence and expression of SIX effectors in Fusarium oxysporum. Mol Plant Pathol.

61. Ghag SB, Shekhawat UK, Ganapathi TR (2014) Host-induced post-transcriptional hairpin RNA-mediated gene silencing of vital fungal genes confers efficient resistance against Fusarium wilt in banana. Plant Biotechnol J 12: 541–553. doi: 10.1111/pbi.12158 PMID: 24476152

62. Son H, Seo YS, Min K, Park AR, Lee J, et al. (2011) A phenome-based functional analysis of transcription factors in the cereal head blight fungus, Fusarium graminearum. PLoS Pathog 7: e1002310. doi: 10.1371/journal.ppat.1002310 PMID: 22028654

63. Guo L, Zhao G, Xu JR, Kistler HC, Gao L, et al. (2016) Compartmentalized gene regulatory network of the pathogenic fungus Fusarium graminearum. New Phytol 211: 527–541. doi: 10.1111/nph.13912 PMID: 26990214

64. van der Does HC, Lievens B, Claes L, Houterman PM, Cornelissen BJ, et al. (2008) The presence of a virulence locus discriminates Fusarium oxysporum isolates causing tomato wilt from other isolates.

Environmental microbiology 10: 1475–1485. doi: 10.1111/j.1462-2920.2007.01561.x PMID: 18312397

65. Takken FL, Van Wijk R, Michielse CB, Houterman PM, Ram AF, et al. (2004) A one-step method to convert vectors into binary vectors suited for *Agrobacterium*-mediated transformation. Curr Genet 45: 242–248. doi: 10.1007/s00294-003-0481-5 PMID: 14745506

66. Rep M, van der Does HC, Meijer M, van Wijk R, Houterman PM, et al. (2004) A small, cysteine-rich protein secreted by *Fusarium oxysporum* during colonization of xylem vessels is required for *I-3*-mediated resistance in tomato. Molecular microbiology 53: 1373–1383. doi: 10.1111/j.1365-2958.2004.04177.x PMID: 15387816

67. Berger MF, Badis G, Gehrke AR, Talukder S, Philippakis AA, et al. (2008) Variation in homeodomain DNA binding revealed by high-resolution analysis of sequence preferences. Cell 133: 1266–1276. doi: 10.1016/j.cell.2008.05.024 PMID: 18585359

68. Philippakis AA, Qureshi AM, Berger MF, Bulyk ML (2008) Design of compact, universal DNA microarrays for protein binding microarray experiments. J Comput Biol 15: 655–665. doi: 10.1089/cmb.2007.0114 PMID: 18651798

69. Lam KN, van Bakel H, Cote A, vdV A., Hughes TR (2011) Sequence specificity is obtained from the majority of modular C2H2 zinc-finger arrays. Nucleic Acids Res 39: 4680–4690. doi: 10.1093/nar/gkq1303 PMID: 21321018

70. Weirauch MT, Cote A, Norel R, Annala M, Zhao Y, et al. (2013) Evaluation of methods for modeling transcription factor sequence specificity. Nat Biotechnol 31: 126–134. doi: 10.1038/nbt.2486 PMID: 23354101

71. Narasimhan K, Lambert SA, Yang AW, Riddell J, Mnaimneh S, et al. (2015) Mapping and analysis of Caenorhabditis elegans transcription factor sequence specificities. Elife 4.

72. Weirauch MT, Yang A, Albu M, Cote AG, Montenegro-Montero A, et al. (2014) Determination and inference of eukaryotic transcription factor sequence specificity. Cell 158: 1431–1443. doi: 10.1016/j.cell.2014.08.009 PMID: 25215497

73. Crooks GE, Hon G, Chandonia JM, Brenner SE (2004) WebLogo: a sequence logo generator. Genome Res 14: 1188–1190. doi: 10.1101/gr.849004 PMID: 15173120

74. Altschul SF, Gish W, Miller W, Myers EW, Lipman DJ (1990) Basic local alignment search tool. J Mol Biol 215: 403–410. doi: 10.1016/S0022-2836(05)80360-2 PMID: 2231712

75. Sievers F, Higgins DG (2014) Clustal Omega, accurate alignment of very large numbers of sequences. Methods Mol Biol 1079: 105–116. doi: 10.1007/978-1-62703-646-7_6 PMID: 24170397

76. Capella-Gutierrez S, Silla-Martinez JM, Gabaldon T (2009) trimAl: a tool for automated alignment trimming in large-scale phylogenetic analyses. Bioinformatics 25: 1972–1973. doi: 10.1093/bioinformatics/btp348 PMID: 19505945

77. Guindon S, Dufayard JF, Lefort V, Anisimova M, Hordijk W, et al. (2010) New algorithms and methods to estimate maximum-likelihood phylogenies: assessing the performance of PhyML 3.0. Syst Biol 59: 307–321. doi: 10.1093/sysbio/syq010 PMID: 20525638

78. Chen DC, Yang BC, Kuo TT (1992) One-step transformation of yeast in stationary phase. Curr Genet 21: 83–84. PMID: 1735128

79. Houterman PM, Cornelissen BJ, Rep M (2008) Suppression of plant resistance gene-based immunity by a fungal effector. PLoS pathogens 4: e1000061. doi: 10.1371/journal.ppat.1000061 PMID: 18464895
